# Morphology and pomological characterization of bael [*Aegle marmelos* (L.) Correa] genotypes for climate change mitigation under north-western Himalayas

**DOI:** 10.3389/fpls.2025.1496769

**Published:** 2025-03-18

**Authors:** Prabhdeep Singh, Akash Sharma, Sushil Kumar Gupta, Romesh Kumar Salgotra, Vishal Gupta, Manish Sharma

**Affiliations:** ^1^ Division of Fruit Science, Faculty of Horticulture and Forestry, Sher-e-Kashmir University of Agricultural Sciences and Technology of Jammu, Jammu and Kashmir, Jammu, India; ^2^ Division of Agroforestry, Faculty of Horticulture and Forestry, Sher-e-Kashmir University of Agricultural Sciences and Technology of Jammu, Jammu and Kashmir, Jammu, India; ^3^ Institute of Biotechnology, Sher-e-Kashmir University of Agricultural Sciences and Technology of Jammu, Jammu and Kashmir, Jammu, India; ^4^ Division of Plant Pathology, Faculty of Agriculture, Sher-e-Kashmir University of Agricultural Sciences and Technology of Jammu, Jammu and Kashmir, Jammu, India; ^5^ Division of Statistics and Computer Sciences, Sher-e-Kashmir University of Agricultural Sciences and Technology of Jammu, Jammu and Kashmir, Jammu, India

**Keywords:** bael, genetic diversity, pomological traits, climate adaptation, principal component analysis, cluster analysis

## Abstract

This study investigated the genetic diversity of 80 wild bael genotypes (*Aegle marmelos*) compared to commercial cultivars NB-5 and NB-9, focusing on 16 pomological traits. With the rising temperature impacting perennial fruit crop production and the urgent need for heat- and drought-resistant varieties, bael emerges as a viable option for rainfed areas due to its xerophytic characteristics and ability to withstand high temperatures. Among the collected, wild bael genotype germplasm, JMU-Bael (Sel-27) demonstrated superior traits, including maximum fruit length (12.05 cm), width (11.72 cm), weight (917.65 g), pulp weight (746.81 g), and pulp percentage (81.38%). Correlation matrices revealed significant associations among pomological traits, particularly positive correlations with fruit weight. Principal component analysis (PCA) indicated substantial genetic diversity, with the first two components explaining 63.98% of the cumulative variation. Cluster analysis grouped genotypes into two main clusters, providing insights into their diversity and potential breeding applications. This comprehensive analysis offers valuable insights into the genetic variability and adaptability of bael genotypes under changing climatic conditions in the plains of north-western Himalayan regions.

## 1 Introduction

Under the changing climatic conditions, variability studies have the potential to mitigate major challenges that affect the production of perennial horticultural crops. Reduction in the production pattern of fruits is likely to be caused by a short growing period, which will negatively impact growth and development particularly due to terminal heat stress and decreased water availability. Horticultural crops that are grown in rainfed areas will be primarily affected because of variability in rainfall (Venkateswarlu and Shanker, 2011). In an era of rapid global climate change, the ranges of endemic species are shifting, profoundly affecting biological communities and threatening biodiversity (Hamann et al., 2021; Elsen et al., 2022). Climate change, through direct impacts like temperature and precipitation alterations and indirect effects such as disturbance dynamics and biotic interaction shifts, exacerbates the risk of species decline and extinction (Haq et al., 2023; Huang et al., 2021; Schmeller et al., 2020). Prioritizing landscapes that support biodiversity is critical for conserving threatened species (Zhang et al., 2022, 2023). Fruit trees, experiencing annual climate variations, serve as indicators of climate change impacts, particularly through their phenology (Aslam et al., 2022; Paul et al., 2011).

The impact of technological advancements, such as new cultivars and production management systems, is evident in increased production and productivity. To quantify the impact of climate change on horticultural crops, this study showed the biodiversity of *Aegle marmelos* (bael), a species currently categorized as near threatened (UCN, 2022).

Bael is native to parts of South Asia, but its population is declining due to habitat destruction, resource extraction, and agricultural practices (Vasava et al., 2018; Waheed et al., 2022). Conservation efforts are essential to mitigate these threats and ensure the survival of such vulnerable species. Bael holds notable value in traditional medicine for its therapeutic properties (Sharma et al., 2022). Bael thrives in adverse climates and on marginal land, tolerating temperatures from 50°C to −7°C and altitudes of up to 1,200 m, making it versatile where other fruits struggle to grow (Pathirana et al., 2020). Bael fruits have a high moisture content of nearly 61% and a high nutritional composition; they contain minerals, fat, fiber, protein, carbohydrates, vitamins, amino acids, and fatty acids (Kaur and Kalia, 2017; Sarkar et al., 2020; Singh et al., 2021). Various bioactive compounds including skimmianine, cineole, citral, citronellol, aegeline, lupeol, marmesinin, marmelosin, luvangetin, psoralen, marmelidefagarine, marmin, umbelliferone, lupeolauroptin, xanthotoxin, scopoletin, tembamide, dictamnine, and marmesin have been isolated from different parts of the bael tree, which have been traditionally utilized in ethnomedicine for various purposes such as astringent, antidiarrheal, antipyretic, antiulcer, antidiabetic, antibacterial, antiviral, antifungal, anticancer, analgesic, radioprotective, antimicrobial, and anti-helminthic properties (Maity et al., 2009; Patkar et al., 2012).

Bael fruits have many uses in functional foods also, as it has much potential for processing into goods such as preserves, powder, jam, wine, slab, and syrup (Singh et al., 2014). In India, many of the products are prepared from bael fruits such as bael sherbet, murabba, or syrups. In other countries such as Indonesia and Thailand, ripe bael fruits and their sliced pieces are consumed as food, and syrups are used in making cake ingredients (Baliga et al., 2011). The processing of bael fruits also produces many waste materials such as seeds, fibers, and peel, which also contain many bioactive and pharmaceutical compounds (Bel-Rhlid et al., 2018; Sonawane et al., 2020).

The biodiversity of bael in India is categorized into two groups based on fruit size and attributes. One type is small, featuring bitter pulp and is commonly utilized in ayurvedic preparations due to its high concentrations of marmelosin and psoralen. The other type is larger, with fewer seeds and less fiber, preferred for desserts and processed goods. The north-western Himalayan region is recognized as a natural habitat for numerous bael species owing to favorable agroclimatic conditions. However, due to insufficient awareness of bael biodiversity, some species are on the brink of extinction. Bael is currently classified as a Rare, Endangered and Threatened (RET) species by the Foundation for Revitalisation of Local Health Traditions (FRLHT). Despite the high esteem of the bael tree as an ethnobotanical plant in India, its potential as a financially viable crop remains largely untapped. It is considered an underutilized fruit due to limited knowledge regarding available genetic resources, varietal characterization, and inadequate cultivation practices among growers.

Farmers are experiencing the challenges of identifying commercial cultivars, as they are unfamiliar with the characteristics of many varieties of bael. To identify distinct characteristics of various bael cultivars, the morphological characteristics are equally important to the fruit characteristics. In the absence of a suitable genotype, desirable growth, flowering, and fruit set have not been accomplished. Identification of suitable genotypes for the region is necessary for promoting the productivity, production, and quality of the fruits under semi-arid conditions. However, enormous variability in bael remains unexploited and awaits proper attention on exploration, collection, and maintenance of germplasms for conserving them from the available genetic diversity of bael in nature.

To measure the effects of climate changes on bael fruit crops, we must have adequate information on their physiological responses and effects on productivity, development, growth, and quality of fruit crops. This means that we must have proper knowledge about the cultivating crop and whether it will be suitable for this region or not. The identification of appropriate genotypes for breeding plays a pivotal role in enhancing bael quality. This study aimed to assess and measure the genetic diversity of bael accessions based on pomological attributes through principal component and cluster analysis, thereby facilitating crop enhancement. Such an analysis is instrumental in formulating an efficient breeding approach for genetic advancement. Additionally, the evaluation of fruit variation was conducted to pinpoint accessions harboring potentially beneficial traits that could be promptly integrated into breeding programs.

## 2 Materials and methods

### 2.1 Plant materials

The study encompassed the biodiversity of 80 wild bael genotypes compared along with two commercially cultivated varieties (NB-5 and NB-9) (**Supplementary Table 1**). The main objective of this investigation was to identify superior bael genotypes under climate change scenarios and to assess the range of diversity in their pomological traits.

### 2.2 Site of experiment

**Figure 1:**
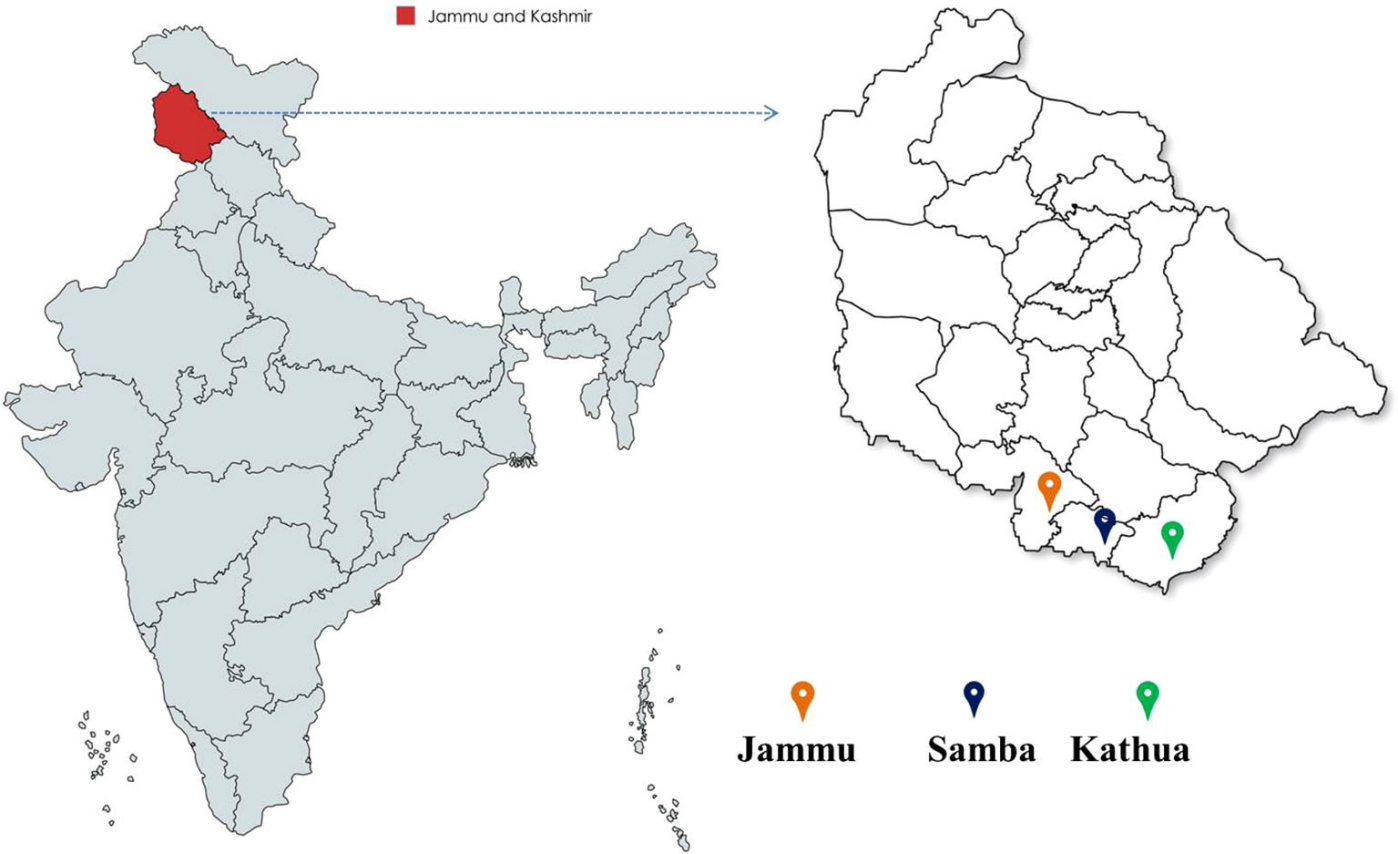
Bael genotypes collected from diverse locations in the Jammu and Kashmir districts of India.

The bael genotypes were selected from the regions of Jammu (32.73°N, 74.87°E, 300 m above sea level), Samba (32.57°N, 75.12°E, 384 m above sea level), and Kathua (32.37°N, 75.52°E, 393 m above sea level) within the Jammu and Kashmir, India (**Figure 1**).

### 2.3 Pomological parameters

For measuring pomological traits, five randomly selected fruits were taken from each replication; they were collected randomly from three different directions of the bael tree, and each direction was considered as one replication. The fruit length, fruit width, fruit skull thickness, inner diameter, seed length, and seed width were measured using a digital vernier caliper. Fruit weight, pulp weight, shell weight/fruit, total seed weight per fruit, and test seed weight per fruit were measured using a digital electronic balance with 0.01-g precision (Debbarma and Hazarika, 2023). The number of seed sacks per fruit, number of seeds per sack, and total number of seeds per fruit were counted manually and expressed in numbers. The pulp percentage (%) was calculated using the following formula: pulp (%) = Pulp weight of the fruit × 100 divided by the total weight of the fruit. The shell percentage (%) was calculated using the following formula: Shell (%) = Shell weight of the fruit × 100 divided by the total weight of the fruit. To identify the superior genotypes, various parameters were evaluated, such as average fruit weight (0.9–1.5 kg), pulp percentage (>80%), skin percentage (<20%), and skin thickness (<2 mm) by consumer preferences (Dhakar et al., 2019). Genotypes having all the above desirable pomological traits were identified as superior.

### 2.4 Morphological parameters

The morphological traits were recorded as per Guidelines for the Conduct of Test for Distinctiveness, Uniformity and Stability of bael (*A. marmelos* Correa) Protection of Plant Varieties and Farmers’ Right Authority (PPV&FRA) Government of India (Patel et al., 2011) and bael descriptor of National Bureau of Plant Genetic Resources (Mahajan et al., 2002).

### 2.5 Statistical analyses

One-way analysis of variance (ANOVA) and least significant difference (LSD) were used to determine variability among bael genotypes using the OriginPro 9.1 software. LSD test was performed at significance levels of *p* < 0.05. Genetic parameters (genotypic coefficient of variation, phenotypic coefficient of variation, heritability, genetic advance, and genetic advance as percentage of mean) and correlation coefficient analysis (phenotypic correlation coefficient and genotypic correlation coefficient) were analyzed using the R software. The relationship among bael genotypes was assessed through principal component analysis (PCA) using the OriginPro 9.1 software. Cluster analysis was conducted utilizing the Euclidean distance coefficient and Ward’s method with the OriginPro 9.1 software. Standardization of distance coefficients was carried out using the Z scale. Furthermore, a scatter plot was generated using the first and second principal components (PC1 and PC2, respectively) in the OriginPro 9.1 software.

## 3 Results

### 3.1 Pomological description

The data on pomological traits of bael genotypes showed a wide range of variations among the collected 80 wild genotypes when compared with the NB-5 and NB-9 cultivars (**Supplementary Table 3**) (**Supplementary Figure 1**). Analysis of variance is a statistical procedure that partitions total variation into different components, allowing for an assessment of specific sources of variation. The mean sum of squares attributed to genotypes revealed a significant difference across all measured traits. This finding indicates substantial genetic variability among bael genotypes, confirming that the genotypes are genetically distinct (**Supplementary Table 2**). The maximum fruit length was recorded in JMU-Bael (Sel-27) (12.05 cm), followed by JMU-Bael (Sel-14) (11.03 cm), while the minimum fruit length (4.60 cm) was found in JMU-Bael (Sel-66). The maximum fruit width (11.72 cm) was recorded in JMU-Bael (Sel-27), followed by JMU-Bael (Sel-74) (10.95 cm), while the minimum fruit width (4.64 cm) was recorded in JMU-Bael (Sel-66). The maximum fruit weight (917.65 g) was observed in JMU-Bael (Sel-27), followed by JMU-Bael (Sel-9) (851.67 g), while the minimum fruit weight (56.33 g) was recorded in JMU-Bael (Sel-66). The maximum pulp weight per fruit was also found in JMU-Bael (Sel-27) (746.81 g), followed by JMU-Bael (Sel-74) (639.12 g), while the minimum pulp weight (34.63 g) was recorded in JMU-Bael (Sel-66). The pulp percentage was the highest in JMU-Bael (Sel-27) (81.38%), followed by JMU-Bael (Sel-58) (78.87%), while the lowest pulp percentage (58.64%) was observed in JMU-Bael (Sel-62). The genotype JMU-Bael (Sel-31) had the maximum shell weight per fruit (225.42 g), followed by NB-9 (225.35 g), while the minimum shell weight per fruit (10.26 g) was found in JMU-Bael (Sel-66). The shell percentage was the highest in JMU-Bael (Sel-51) (30.18%), followed by JMU-Bael (Sel-49) (29.06%), while the lowest shell percentage (16.96%) was recorded in JMU-Bael (Sel-27). The maximum skull thickness (2.99 mm) was observed in JMU-Bael (Sel-52), while the minimum skull thickness (1.80 mm) was found in JMU-Bael (Sel-27). The highest inner diameter (11.54 cm) was recorded in JMU-Bael (Sel-27), while the smallest inner diameter (4.41 cm) was observed in JMU-Bael (Sel-66).

**Figure 2:**
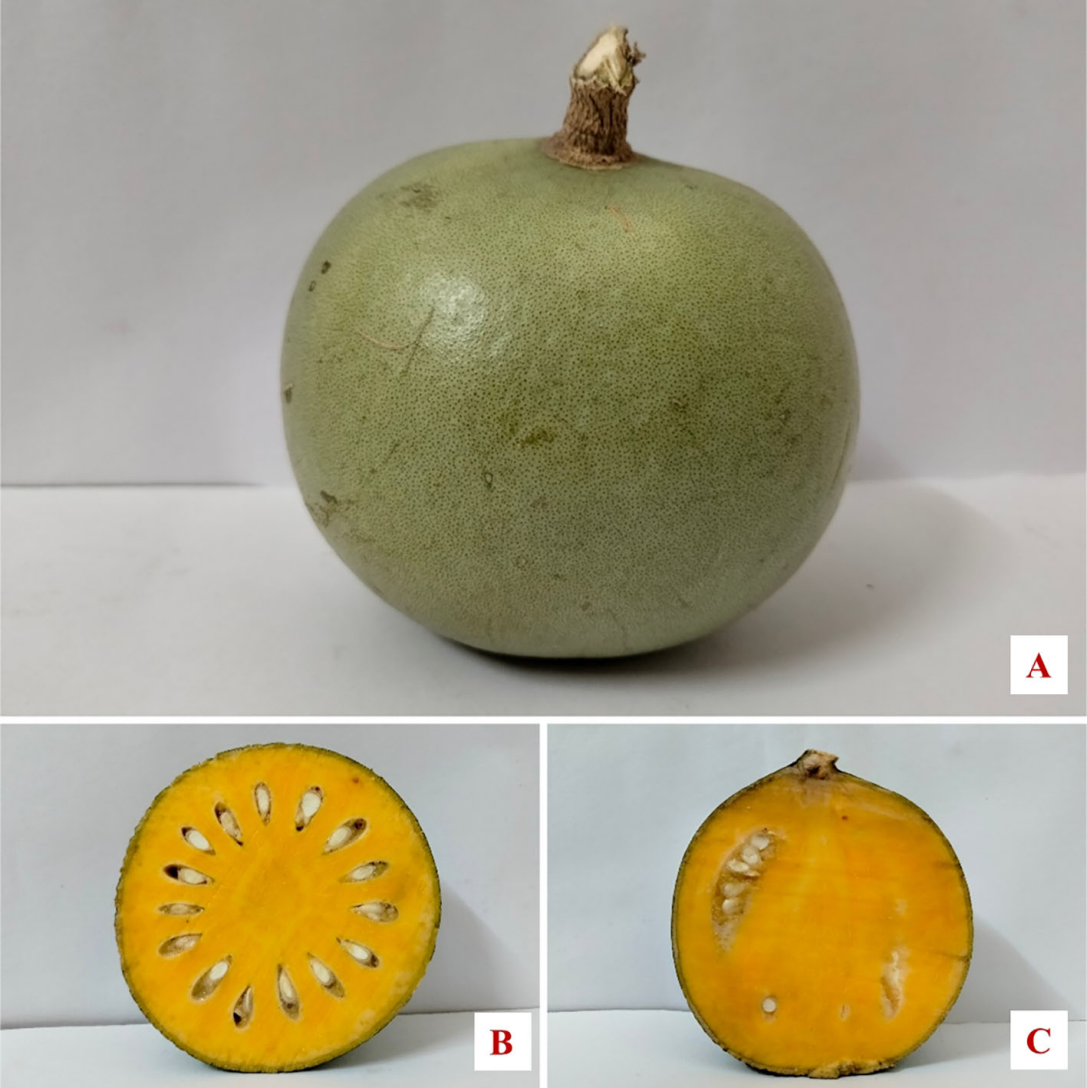
Vivid presentation of superior bael selection JMU-Bael (Sel-27). **(A)** Fruit. **(B)** Horizontal inner core. **(C)** Vertical inner core.

Regarding seed characteristics, the maximum seed length (10.68 mm) was recorded in JMU-Bael (Sel-58), while the minimum seed length (3.70 mm) was found in JMU-Bael (Sel-78). The maximum seed diameter (7.12 mm) was observed in JMU-Bael (Sel-20), and the minimum seed diameter (2.48 mm) in JMU-Bael (Sel-78). The maximum number of seed sacks per fruit (16.67) was recorded in JMU-Bael (Sel-23), followed by JMU-Bael (Sel-7), JMU-Bael (Sel-57), and JMU-Bael (Sel-74) (16.33), while the minimum number of seed sacks per fruit (7.33) was found in JMU-Bael (Sel-46). The maximum number of seeds per seed sack (17.33) was observed in JMU-Bael (Sel-33), while the minimum (2.33) was found in JMU-Bael (Sel-58). The maximum number of seeds per fruit (182.33) was recorded in JMU-Bael (Sel-33), while the minimum (23.33) was observed in JMU-Bael (Sel-46). The maximum seed weight per fruit (46.33 g) was recorded in JMU-Bael (Sel-33), while the minimum (5.13 g) was found in JMU-Bael (Sel-46). The maximum test seed weight (25.65 g) was observed in JMU-Bael (Sel-64), while the minimum test seed weight (14.47 g) was recorded in JMU-Bael (Sel-19). Based on the superior attributes observed in the wild bael genotypes, the pomological analysis revealed that the selection JMU-Bael (Sel-27) exhibited superior characteristics among all the wild bael genotypes and compared with the NB-5 and NB-9 cultivars in terms of pomological traits (**Figure 2**).

### 3.2 Morphological description

The data on morphological traits of bael genotypes showed a wide range of variation among 80 wild bael genotypes compared with the NB-5 and NB-9 cultivars (**Supplementary Table 4**). Based on the fruiting season, the early fruiting season was found in 12 genotypes, mid-season in 43 genotypes, and late season in 25 bael genotypes as compared to NB-5 having a late type and NB-9 having a mid type of fruiting season. Based on the date of fruit maturity, 12 genotypes were observed to be the earliest to mature in the fourth week of March, whereas 25 genotypes fruit maturity in the third week of April, while the average date of fruit maturity among the remaining 43 genotypes was found on the first and second weeks of April as compared to NB-5 having the third week of April and NB-9 having date of fruit maturity in the first week of April. Based on immature fruit color, light green color was recorded in 56 genotypes, green color in 18 genotypes, and dark green immature color of fruit in six genotypes when compared to NB-5 having a green type and NB-9 having a dark-green type of immature fruit color. Based on mature fruit color, the results showed green color in 12 bael genotypes, greenish pale yellow in 57 genotypes, and yellowish green color of mature fruit in 11 genotypes as compared to NB-5 having greenish pale yellow and NB-9 having a green type of mature fruit color.

Based on the stylar end cavity, 77 genotypes had shallow and three genotypes had depressed stylar end cavity as compared to commercially growing bael cultivars NB-5 having a depressed type and NB-9 having a shallow type of stylar end cavity. Based on stem end cavity, a shallow stem end cavity was observed in 74 bael genotypes, and depressed stem end cavity was found in six genotypes as compared to NB-5 and NB-9 having a shallow type of stem end cavity. Based on the fruit surface, a smooth surface was observed in 59 genotypes while a rough surface in 21 genotypes as compared to NB-5 having a smooth type and NB-9 having a rough type of fruit surface. Based on fruit shape, 22 genotypes had a globose fruit shape, four genotypes had ovate, 10 genotypes had elliptical, and 44 genotypes had round fruit shape as compared to NB-5 having a round type and NB-9 having an ovate type of fruit shape. The results on skull color revealed that dull white skull color was observed in two genotypes, creamish yellow skull color was observed in 12 genotypes, greenish-yellow skull color in 47 genotypes, russet-yellow skull color in five genotypes, and greenish skull color in 14 bael genotypes as compared to NB-5 having a creamish yellow type and NB-9 having a greenish type of skull color. Studies conducted on locule arrangement showed scattered locule arrangement in five genotypes, centric locule arrangement in 69 genotypes, and highly centric locule arrangement in six genotypes as compared to bael cultivars NB-5 having a centric type and NB-9 having a highly centric type of locule arrangement. Based on the arrangement of seeds, a straight line was observed in 78 bael genotypes, and seeds were distributed in whole pulp found in two bael genotypes as compared to NB-5 and NB-9 bael cultivars having an arrangement of seeds in a straight line. Studies on seed shape showed that bael genotypes were differentiated round and oblong, whereas 24 genotypes had round seed shape, and the remaining 56 had an oblong shape of seeds as compared to NB-5 having a round shape and NB-9 having an oblong type of seed shape.

### 3.3 Genetic parameters

Environmental coefficient of variation (ECV), genotypic coefficient of variation (GCV), phenotypic coefficient of variation (PCV), heritability in broad sense (h^2^b), genetic advance (GA), and genetic advance in percentage of the mean (GAM) were estimated for 16 pomological traits (**Table 1**). The highest values for h^2^b and GA were recorded in fruit weight, and the ECV was recorded in the number of seeds per sack. Shell weight/fruit (g) recorded higher percentages of GCV, PCV, and GAM. The lowest values for ECV, GCV, and PCV were recorded in pulp percentage; the lowest GA value was recorded in fruit skull thickness; and the lowest GAM value was recorded in pulp percentage. The 12 pomological characteristics gave higher values for the broad-sense heritability (>98%). These findings suggest that these traits are amenable to improvement through selective breeding.

**Table 1:** Estimates of variability parameters for various pomological traits of bael.

Characteristics	Coefficient of variation (%)			Heritability % (Broad Sense)	Genetic advancement	Genetic advance as % of mean
	ECV	GCV	PCV			
Fruit length	1.92	20.99	21.08	99.17	3.56	43.06
Fruit width	1.78	22.18	22.25	99.36	3.65	45.55
Fruit weight	1.30	63.92	63.93	99.96	481.53	131.64
Pulp weight	1.44	66.94	66.96	99.95	358.47	137.86
Pulp	1.21	7.96	8.05	97.74	11.20	16.21
Shell weight/fruit	2.59	67.67	67.72	99.85	120.38	139.30
Shell	1.32	13.45	13.52	99.05	6.45	27.58
Fruit skull thickness	2.96	10.37	10.79	92.42	0.54	20.55
Inner diameter	1.81	22.93	23.00	99.38	3.64	47.10
Seed length	2.15	19.88	19.99	98.84	2.80	40.71
Seed diameter	2.08	19.79	19.90	98.91	2.13	40.55
Number of seed sacks per fruit	4.60	18.31	18.88	94.06	4.37	36.59
Number of seeds per sack	6.16	37.83	38.33	97.42	6.53	76.92
Number of seeds per fruit	2.50	40.99	41.07	99.63	81.51	84.28
Total seed weight per fruit	4.12	48.02	48.19	99.27	19.07	98.55
Test seed weight per fruit	5.42	11.55	12.75	81.97	4.25	21.54

ECV, environmental coefficient of variation; GCV, genotypic coefficient of variation; PCV, phenotypic coefficient of variation.

### 3.4 Correlation matrices

The fruit length exhibited highly strong correlations with a multitude of traits including fruit width, fruit weight, pulp weight, pulp percentage, shell weight, inner diameter, seed length, seed diameter, number of seed sacks per fruit, number of seeds per fruit, and total seed weight per fruit. Similarly, fruit width demonstrated strong associations with several traits, including fruit weight, pulp weight, pulp percentage, shell weight per fruit, inner diameter, seed length, seed diameter, number of seed sacks per fruit, number of seeds per sack, number of seeds per fruit, and total seed weight per fruit. Fruit weight also displayed strong correlations with multiple traits, such as pulp weight, pulp percentage, shell weight per fruit, inner diameter, seed length, seed diameter, number of seed sacks per fruit, number of seeds per sack, number of seeds per fruit, and total seed weight per fruit. Conversely, pulp percentage showed highly strong negative correlations with certain traits such as shell percentage, fruit skull thickness, and number of seeds per sack (**Tables 2**, **3**).

**Table 2:** Genotypic correlation coefficient among various pomological traits of bael.

Characteristics	Fruit length	Fruit width	Fruit weight	Pulp weight	Pulp percentage	Shell weight/ fruit	Shell percentage	Fruit skull thickness	Inner diameter	Seed length	Seed diameter	Number of seed sacks per fruit	Number of seeds per sack	Number of seeds per fruit	Total seed weight per fruit	Test seed weight per fruit
Fruit length	1.000	0.810**	0.832**	0.838**	0.650**	0.794**	0.113	0.052	0.809**	0.519**	0.585**	0.235*	0.164	0.267*	0.242*	0.004
Fruit width		1.000	0.908**	0.910**	0.631**	0.857**	0.056	0.039	0.999**	0.461**	0.546**	0.426**	0.276*	0.417**	0.414**	0.127
Fruit weight			1.000	0.995**	0.581**	0.970**	0.121	0.019	0.908**	0.469**	0.555**	0.413**	0.253*	0.408**	0.411**	0.136
Pulp weight				1.000	0.638**	0.946**	0.053	−0.037	0.911**	0.474**	0.559**	0.420**	0.204	0.361**	0.361**	0.099
Pulp percentage					1.000	0.443**	−0.446**	−0.427**	0.638**	0.328**	0.415**	0.307**	−0.267*	−0.126	−0.131	−0.132
Shell weight/fruit						1.000	0.315**	0.175	0.855**	0.431**	0.510**	0.348**	0.265*	0.403**	0.412**	0.165
Shell percentage							1.000	0.802**	0.044	0.069	−0.010	−0.157	0.108	0.067	0.061	0.052
Fruit skull thickness								1.000	0.023	0.002	0.034	−0.080	0.124	0.099	0.060	−0.024
Inner diameter									1.000	0.462**	0.546**	0.428**	0.275*	0.416**	0.414**	0.128
Seed length										1.000	0.659**	0.310**	0.161	0.241*	0.210	−0.050
Seed diameter											1.000	0.312**	0.212	0.303**	0.282*	0.038
Number of seed sacks per fruit												1.000	−0.030	0.391**	0.348**	−0.019
Number of seeds per sack													1.000	0.889**	0.874**	0.336**
Number of seeds per fruit														1.000	0.969**	0.327**
Total seed weight per fruit															1.000	0.536**
Test seed weight per fruit																1.000

** and * indicate levels of significance at 5% and 1%, respectively.

**Table 3:** Phenotypic correlation coefficient among various pomological traits of bael.

Characteristics	Fruit length	Fruit width	Fruit weight	Pulp weight	Pulp percentage	Shell weight/ fruit	Shell percentage	Fruit skull thickness	Inner diameter	Seed length	Seed diameter	Number of seed sacks per fruit	Number of seeds per sack	Number of seeds per fruit	Total seed weight per fruit	Test seed weight per fruit
Fruit length	1.000	0.810**	0.831**	0.837**	0.647**	0.793**	0.112	0.053	0.809**	0.517**	0.583**	0.233**	0.164*	0.266**	0.242**	0.004
Fruit width		1.000	0.908**	0.909**	0.628**	0.856**	0.056	0.041	0.999**	0.460**	0.545**	0.422**	0.275**	0.416**	0.413**	0.123
Fruit weight			1.000	0.995**	0.578**	0.970**	0.120	0.019	0.908**	0.468**	0.554**	0.409**	0.252**	0.408**	0.411**	0.131
Pulp weight				1.000	0.636**	0.945**	0.053	−0.036	0.910**	0.473**	0.558**	0.415**	0.203**	0.361**	0.360**	0.095
Pulp percentage					1.000	0.441**	−0.445**	−0.419**	0.635**	0.326**	0.413**	0.302**	−0.265**	−0.126	−0.133*	−0.141*
Shell weight/fruit						1.000	0.315**	0.173**	0.854**	0.430**	0.509**	0.344**	0.263**	0.403**	0.411**	0.159*
Shell percentage							1.000	0.791**	0.044	0.069	−0.010	−0.155*	0.108	0.067	0.060	0.050
Fruit skull thickness								1.000	0.025	0.003	0.038	−0.077	0.122	0.097	0.059	−0.022
Inner diameter									1.000	0.461**	0.545**	0.423**	0.273**	0.415**	0.413**	0.124
Seed length										1.000	0.657**	0.307**	0.161*	0.241**	0.210**	−0.049
Seed diameter											1.000	0.310**	0.210**	0.302**	0.281**	0.037
Number of seed sacks per fruit												1.000	−0.030	0.386**	0.344**	−0.016
Number of seeds per sack													1.000	0.887**	0.869**	0.315**
Number of seeds per fruit														1.000	0.967**	0.312**
Total seed weight per fruit															1.000	0.526**
Test seed weight per fruit																1.000

** and * indicate levels of significance at 5% and 1%, respectively.

### 3.5 Principal component analysis

**Figure 3:**
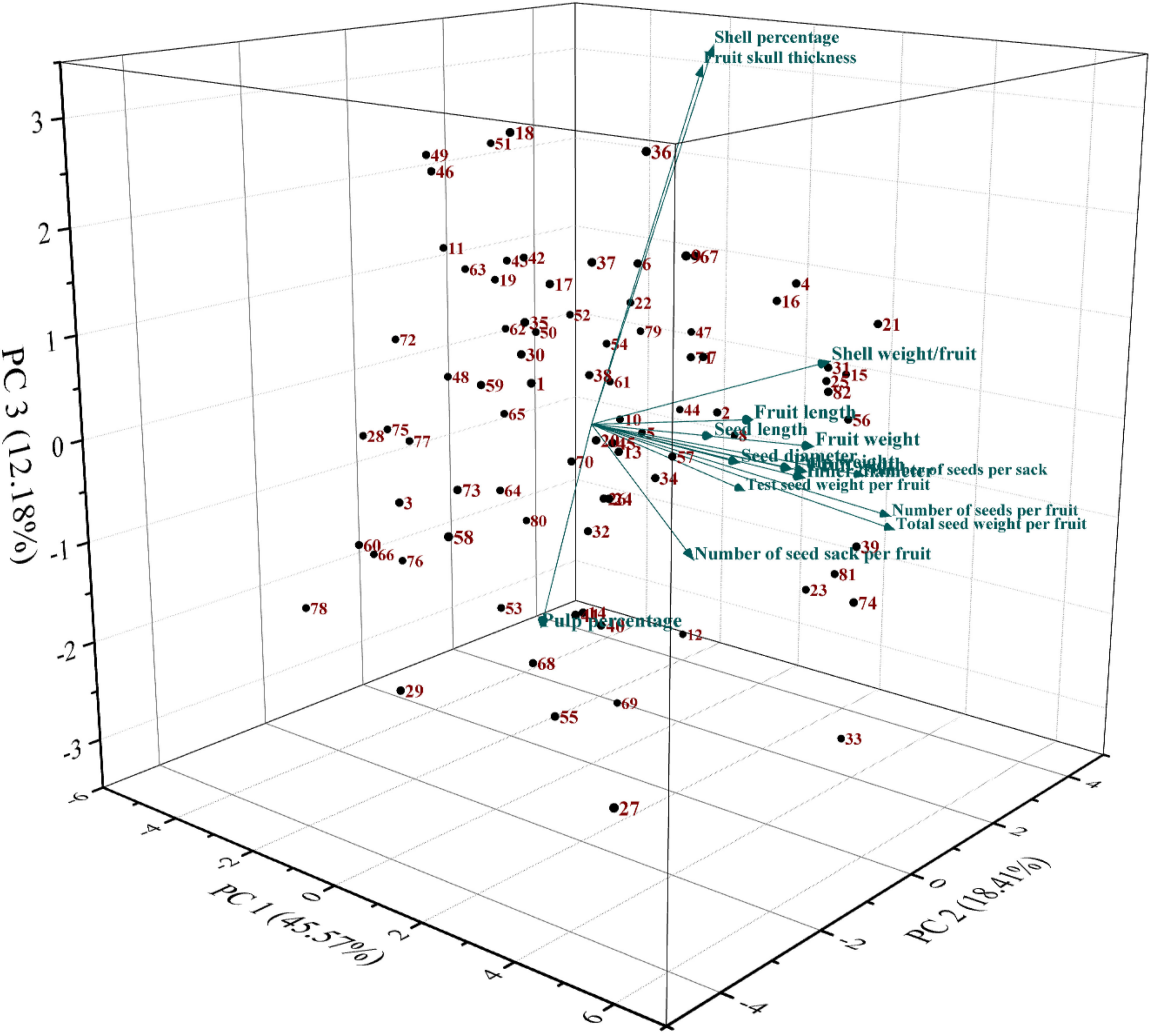
Three-dimensional PCA plot of the studied bael genotypes based on PC1/PC2. PCA, principal component analysis.

PCA was conducted to assess the genetic diversity present among bael genotypes based on their pomological traits. The PCA involved analyzing eigenvalues, variabilities, cumulative variabilities, and eigenvectors and generating a scree plot. Higher eigenvalues indicate greater genetic variabilities among the bael genotypes; however, the variation in cumulative variability showed an inverse relationship. PC1 and PC2, the first two principal components, collectively accounted for 63.98% of the cumulative variation. PC1, with an eigenvalue of 7.29, explained 45.57% of the total variation, while PC2, PC3, PC4, and PC5 contributed 18.41%, 12.18%, 6.76%, and 5.47% variations, respectively. PC1 exhibited significant factor loadings for fruit-related traits such as fruit weight, pulp weight, fruit width, inner diameter, and shell weight per fruit, explaining 45.57% of the total variance. Conversely, PC2 showed significant factor loadings for traits like the number of seeds per sack, total seed weight per fruit, and number of seeds per fruit, contributing 18.41% of the total variance. Traits deemed important for PC1 included fruit length, fruit width, fruit weight, pulp weight, inner diameter, and shell weight per fruit. Utilizing this analysis, individuals with higher scores from comprehensive evaluations were selected. A scatter plot based on PC1 and PC2 was constructed to visualize the diversity in pomological traits among the bael genotypes (**Figure 3**).

### 3.6 Cluster analysis

**Figure 4:**
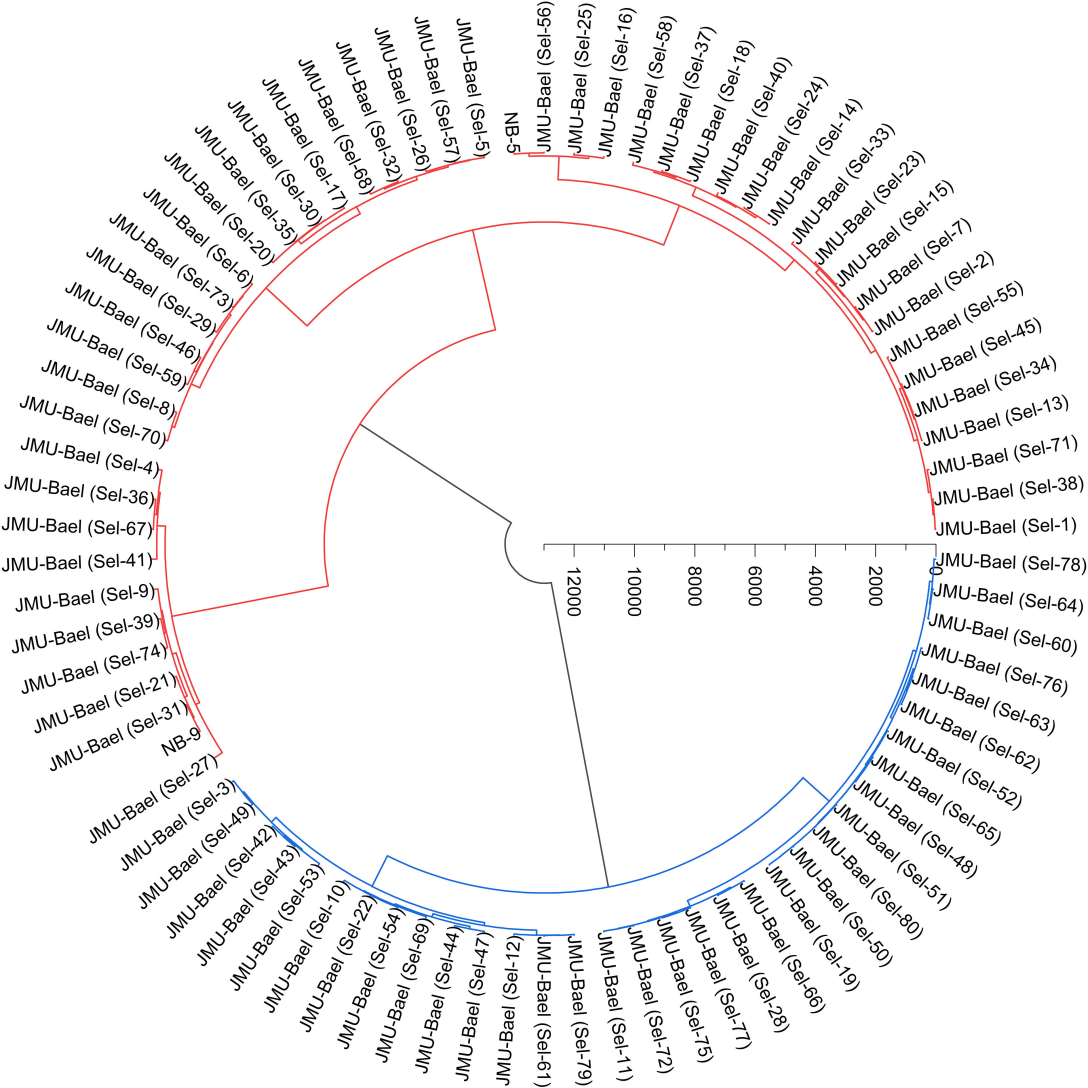
Ward’s cluster analysis of the studied bael genotypes based on pomological traits using Euclidean distances. The results show that the populations were divided into two categories, which are indicated by red and blue colors.

In this study, Ward’s method was employed to conduct cluster analysis on 16 pomological traits of 80 wild bael genotypes along with two commercial cultivars, NB-5 and NB-9. The analysis revealed two main clusters, each containing sub-clusters, based on the pomological traits. Cluster I consisted of 49 genotypes, while Cluster II included 33 bael genotypes (**Figure 4**).

## 4 Discussion

Significant differences in both the pomological traits and morphological characteristics have been observed among closely related genotypes of bael fruits during the studies. This variation in fruit characteristics may be governed by genomic characteristics associated with the genotypes and also due to acclimatized conditions, which were more favorable for the wild bael genotypes; hence, the environment plays an important role in growth parameters. The other probable reason may be due to the fertility status of soil, micro-climate, and inherent characteristics of germplasm. Our results are in accordance with the findings of Debbarma and Hazarika (2023), who studied the variation in fruit characteristics in 30 bael accessions. In a similar study, Dhakar et al. (2019) also evaluated the genotypic diversity among 75 bael genotypes based on their fruit characteristics. These results are in line with Nagar et al. (2017, 2018), who evaluated different fruit characteristics in 12 bael germplasm. Similarly, Sarker et al. (2015) also studied the fruit characteristics of seven bael cultivars. These results on morphological traits are in agreement with those of Parihar (2015), who reported the color of immature fruit, color of mature fruit, and fruit shape of 20 bael genotypes. Similar findings by Nagar (2016) also reported the variations in fruit apex, fruit base, and fruit shape with respect to 12 bael germplasms; similarly, Pavani et al. (2017) observed different fruit characteristics of 20 bael genotypes.

Genetic variance and heritability play vital roles in improving crops through selective breeding. High GCV and PCV indicate more inheritable genetic variation, which can be used to develop superior cultivars. The lower GCV and PCV imply limited potential for improvement through selection, highlighting the importance of assessing these metrics in breeding programs. In vegetatively propagated crops such as bael, estimating broad-sense heritability is especially useful, as it considers both additive and non-additive genetic components passed to offspring. Previous studies on bael have reported high GCV, PCV, and heritability across various traits in bael, demonstrating significant heritable variation (Debbarma and Hazarika, 2023). Our findings are also closely related to the findings of Sharma et al. (2024) on walnuts and Alqahtani et al. (2023) on date palms, further supporting the notion that these traits can be effectively improved through selective breeding. The substantial heritable variation in bael provides a robust foundation for breeding programs aimed at enhancing desirable characteristics, both economically and nutritionally. This genetic diversity not only facilitates the development of superior cultivars but also contributes to the resilience of bael fruits in changing environmental conditions. By focusing on genotypes with high heritability and variance, breeders can optimize selection strategies to achieve significant advancements in bael cultivation.

Correlations at both genetic and phenotypic levels play a significant role in the selection process for parental plants in breeding programs. These correlations are essential for breeders to identify and choose parents with desired traits. In this study, highly significant correlations (*p* < 0.01) were observed among all pomological traits examined. Specifically, fruit length, fruit width, and fruit weight exhibited significant positive correlations with other traits at both the genotypic and phenotypic levels. The correlation coefficients among all traits were further analyzed to delineate genotypic and phenotypic relationships. These analyses, based on error variance and covariance matrices of pomological traits, revealed highly significant associations among all traits examined. Notably, significant relationships were found among all pomological characteristics at both genotypic and phenotypic levels. However, it was observed that pulp percentage exhibited negative correlations with other traits. Moreover, certain traits displayed particularly strong associations. The present study revealed that for most traits, the genotypic correlation coefficients were of a higher magnitude than the phenotypic ones, indicating the predominant role of heritable factors. Genetic relation of traits may result from pleiotropic effects of a gene, linkage of two genes, chromogema, and regimental affiliation or environmental influences. These results are in conformity with the findings of Debbarma and Hazarika (2023), who studied the genotypic correlation and phenotypic correlation of morphological characteristics in 30 bael accessions.

Plotting relationships among individuals in two or more dimensions using PCA enables a more realistic interpretation of those relationships (Crovello, 1970). PCA reduces dimensionality, which looks at data to find relationships between objects, estimate the correlation structure of variables, and find the minimum number of components (a linear combination of the original features) required to explain the majority of the variance with the least amount of information loss (Rodriguez-Delgado et al., 2002; Samec et al., 2016). To develop higher-quality bael genotypes, it is essential to identify and characterize the genetic variability present in bael. According to PCA, the genetic diversity among genotypes may be influenced by elements such as heterogeneity, population genetic architecture, and developmental features (Murty and Arunachalam, 1966). In the current study, genotype similarities and differences were assessed using PCA with pomological variables. PCA generally verified that the pomological features were arranged into species-specific clusters. The results revealed that the initial first two principal components collectively contributed 63.98% of the cumulative variance, suggesting that they could effectively represent the majority of bael traits, potentially indicating the integration of these traits. Individuals with higher scores from comprehensive evaluation were subsequently chosen through this analysis. PCA indicates that the genetic diversity among the genotypes could be due to factors like heterogeneity, genetic architecture of the populations, and developmental traits. A similar finding was observed by Debbarma and Hazarika (2023), who conducted principal component analysis of morphological characteristics in 30 bael accessions. Similarly, Dhakar et al. (2019) also conducted principal component analysis among 75 genotypes. Similar findings were also observed by Zhu et al. (2022) in *Camellia oleifera*, Xin et al. (2022) in calamansi, Gecer et al. (2020) in apricot, Khadivi et al. (2020) in *Pyrus syriaca*, Moradi et al. (2019) in Cornelian cherry, Khadivi et al. (2018) in edible fig, and Kumar et al. (2018) in wild apple.

Cluster analysis condenses data into a singular meaningful dimension, revealing notable distinctions among bael genotypes. In this study, Cluster I encompassed 49 genotypes, while Cluster II included 33 bael genotypes. A similar study was reported by Debbarma and Hazarika (2023), who studied the cluster analysis of various morphological characteristics in 30 bael accessions. Similarly, Dhakar et al. (2019) also studied cluster analysis based on fruit traits of 75 genotypes of bael. Similar studies were conducted on other fruit crops, *viz*., *Camellia oleifera* (Zhu et al., 2022), calamansi (Xin et al., 2022), apricot (Gecer et al., 2020), *P. syriaca* (Khadivi et al., 2020), Cornelian cherry (Moradi et al., 2019), edible fig (Khadivi et al., 2018), and wild apple (Kumar et al., 2018).

### 4.1 Desirable genotypes for further breeding program

JMU-Bael (Sel-27) exhibited the maximum fruit length, fruit width, fruit weight, pulp weight per fruit, pulp percentage, inner diameter, shell percentage, and fruit skull thickness. Importantly, this genotype had average fruit weight (0.9–1.5 kg), pulp percentage (>80%), skin percentage (<20%), and skin thickness (<2 mm); these specific parameters were considered based on consumer preferences (Dhakar et al., 2019). This genotype, with its superior pomological traits, represents a valuable genetic resource for future breeding programs targeting the development of enhanced cultivars that meet global regulatory standards.

## 5 Conclusion

This study highlights the significant diversity of wild bael genotypes in the Jammu region of India. Among the 80 selected genotypes, JMU-Bael (Sel-27) showed superior traits in terms of pomological characteristics for adaptation to changing climatic conditions. This genotype, having excellent traits of interest and high genetic dissimilarity, can be further used in breeding programs to obtain segregates, and accordingly, the existing bael populations can serve as a genetic resource for bael variety development for commercial cultivation under changing climate scenarios.

## Data Availability

The datasets presented in this study can be found in online repositories. The names of the repository/repositories and accession number(s) can be found in the article/**Supplementary Material**.
