# Supplementary material for: Morphology and pomological characterization of bael [Aegle marmelos (L.) Correa] genotypes for climate change mitigation under north-western Himalayas
Source: Front Plant Sci. 2025 Mar 18;16:1496769. doi: 10.3389/fpls.2025.1496769 (PMC11962431; doi:10.3389/fpls.2025.1496769)
Supplement: Supplementary file 1 [file DataSheet1.docx]

**Supplementary material**

**Pomological Characterization of Bael [*Aegle marmelos* (L.) Correa] Genotypes for Climate Change Mitigation in the North-Western Himalayan**

**Table S1. Code and geographical location of wild bael genotypes and commercial bael cultivars.**

| **S. No.** | **Code** | **Area/Block** | **Latitude (^o^)** | **Longitude (^o^)** | **Elevation**  **(m above msl)** |
| --- | --- | --- | --- | --- | --- |
|  | JMU-Bael (Sel-1) | Narval Pain, Jammu, J&K, India | 32^o^ 40. 790 | 074^o^ 49. 697 | 279 |
|  | JMU-Bael (Sel-2) | Miran Sahib, Jammu, J&K, India | 32^o^ 38. 510 | 074^o^ 48. 607 | 284 |
|  | JMU-Bael (Sel-3) | Miran Sahib, Jammu, J&K, India | 32^o^ 38. 480 | 074^o^ 48. 779 | 282 |
|  | JMU-Bael (Sel-4) | Badayal Quzian, Jammu, J&K, India | 32^o^ 35. 165 | 074^o^ 43. 662 | 272 |
|  | JMU-Bael (Sel-5) | Chakrohi, Jammu, J&K, India | 32^o^ 32. 721 | 074^o^ 43. 114 | 265 |
|  | JMU-Bael (Sel-6) | Hansa, Jammu, J&K, India | 32^o^ 32. 785 | 074^o^ 43. 789 | 268 |
|  | JMU-Bael (Sel-7) | R.S Pura, Jammu, J&K, India | 32^o^ 36. 292 | 074^o^ 43. 942 | 283 |
|  | JMU-Bael (Sel-8) | Bishnah, Jammu, J&K, India | 32^o^ 36. 977 | 074^o^ 51. 274 | 290 |
|  | JMU-Bael (Sel-9) | Bishnah, Jammu, J&K, India | 32^o^ 36. 829 | 074^o^ 51. 369 | 287 |
|  | JMU-Bael (Sel-10) | Bishnah, Jammu, J&K, India | 32^o^ 36. 790 | 074^o^ 51. 432 | 291 |
|  | JMU-Bael (Sel-11) | Kotli, Jammu, J&K, India | 32^o^ 37. 847 | 074^o^ 49. 760 | 298 |
|  | JMU-Bael (Sel-12) | Raipur Jangir, Jammu, J&K, India | 32^o^ 43. 100 | 074^o^ 46. 550 | 267 |
|  | JMU-Bael (Sel-13) | Raipur Jangir, Jammu, J&K, India | 32^o^ 43. 099 | 074^o^ 46. 553 | 268 |
|  | JMU-Bael (Sel-14) | Raipur Jangir, Jammu, J&K, India | 32^o^ 43. 097 | 074^o^ 46. 554 | 269 |
|  | JMU-Bael (Sel-15) | Raipur Jangir, Jammu, J&K, India | 32^o^ 43. 104 | 074^o^ 46. 557 | 268 |
|  | JMU-Bael (Sel-16) | Poni Chak, Jammu, J&K, India | 32^o^ 44. 379 | 074^o^ 48. 070 | 278 |
|  | JMU-Bael (Sel-17) | Poni Chak, Jammu, J&K, India | 32^o^ 44. 380 | 074^o^ 48. 076 | 279 |
|  | JMU-Bael (Sel-18) | Akal, Jammu, J&K, India | 32^o^ 40. 557 | 074^o^ 49. 570 | 274 |
|  | JMU-Bael (Sel-19) | Hakkal, Jammu, J&K, India | 32^o^ 40. 770 | 074^o^ 49. 009 | 268 |
|  | JMU-Bael (Sel-20) | Talab Tillo, Jammu, J&K, India | 32^o^ 44. 200 | 074^o^ 50. 058 | 295 |
|  | JMU-Bael (Sel-21) | Damana, Jammu, J&K, India | 32^o^ 47. 214 | 074^o^ 47. 091 | 315 |
|  | JMU-Bael (Sel-22) | Damana, Jammu, J&K, India | 32^o^ 47. 208 | 074^o^ 47. 089 | 301 |
|  | JMU-Bael (Sel-23) | Mishri Wala, Jammu, J&K, India | 32^o^ 48. 396 | 074^o^ 46. 354 | 301 |
|  | JMU-Bael (Sel-24) | Pandorian, Jammu, J&K, India | 32^o^ 48. 108 | 074^o^ 45. 605 | 293 |
|  | JMU-Bael (Sel-25) | Pandorian, Jammu, J&K, India | 32^o^ 48. 115 | 074^o^ 45. 607 | 294 |
|  | JMU-Bael (Sel-26) | Gobindpura, Jammu, J&K, India | 32^o^ 40. 699 | 074^o^ 49. 953 | 259 |
|  | JMU-Bael (Sel-27) | Gobindpura, Jammu, J&K, India | 32^o^ 40. 552 | 074^o^ 49. 986 | 242 |
|  | JMU-Bael (Sel-28) | Old Satwari, Jammu, J&K, India | 32^o^ 40. 858 | 074^o^ 49. 713 | 259 |
|  | JMU-Bael (Sel-29) | Satwari, Jammu, J&K, India | 32^o^ 41. 086 | 074^o^ 50. 451 | 283 |
|  | JMU-Bael (Sel-30) | Satwari, Jammu, J&K, India | 32^o^ 41. 262 | 074^o^ 50. 822 | 288 |
|  | JMU-Bael (Sel-31) | Satwari, Jammu, J&K, India | 32^o^ 41. 260 | 074^o^ 50. 823 | 287 |
|  | JMU-Bael (Sel-32) | Satwari, Jammu, J&K, India | 32^o^ 41. 303 | 074^o^ 50. 795 | 290 |
|  | JMU-Bael (Sel-33) | Satwari, Jammu, J&K, India | 32^o^ 41. 464 | 074^o^ 50. 946 | 293 |
|  | JMU-Bael (Sel-34) | Satwari, Jammu, J&K, India | 32^o^ 41. 469 | 074^o^ 50. 955 | 283 |
|  | JMU-Bael (Sel-35) | Chatha, Jammu, J&K, India | 32^o^ 39. 941 | 074^o^ 49. 384 | 271 |
|  | JMU-Bael (Sel-36) | Chatha, Jammu, J&K, India | 32^o^ 39. 981 | 074^o^ 49. 440 | 272 |
|  | JMU-Bael (Sel-37) | Chatha, Jammu, J&K, India | 32^o^ 39. 779 | 074^o^ 49. 377 | 268 |
|  | JMU-Bael (Sel-38) | Vijaypur, Samba, J&K, India | 32^o^ 33. 911 | 075^o^ 01. 299 | 340 |
|  | JMU-Bael (Sel-39) | Kuralia, Samba, J&K, India | 32^o^ 32. 176 | 074^o^ 59.212 | 320 |
|  | JMU-Bael (Sel-40) | Kuralia, Samba, J&K, India | 32^o^ 31. 507 | 074^o^ 59. 102 | 311 |
|  | JMU-Bael (Sel-41) | Kuralia, Samba, J&K, India | 32^o^ 31. 545 | 074^o^ 58. 989 | 310 |
|  | JMU-Bael (Sel-42) | Rara, Samba, J&K, India | 32^o^ 33. 350 | 074^o^ 57. 612 | 320 |
|  | JMU-Bael (Sel-43) | Rara, Samba, J&K, India | 32^o^ 33. 335 | 074^o^ 57. 610 | 321 |
|  | JMU-Bael (Sel-44) | Rara, Samba, J&K, India | 32^o^ 33. 334 | 074^o^ 57. 616 | 322 |
|  | JMU-Bael (Sel-45) | Thalori, Samba, J&K, India | 32^o^ 34. 390 | 075^o^ 00. 982 | 351 |
|  | JMU-Bael (Sel-46) | Thalori, Samba, J&K, India | 32^o^ 34. 554 | 075^o^ 01. 512 | 366 |
|  | JMU-Bael (Sel-47) | Gurha, Samba, J&K, India | 32^o^ 35. 842 | 075^o^ 03. 083 | 378 |
|  | JMU-Bael (Sel-48) | Uttervehni, Samba, J&K, India | 32^o^ 38. 966 | 075^o^ 03. 617 | 403 |
|  | JMU-Bael (Sel-49) | Uttervehni, Samba, J&K, India | 32^o^ 39.302 | 075^o^ 03. 715 | 421 |
|  | JMU-Bael (Sel-50) | Uttervehni, Samba, J&K, India | 32^o^ 39. 294 | 075^o^ 03. 676 | 393 |
|  | JMU-Bael (Sel-51) | Uttervehni, Samba, J&K, India | 32^o^ 39. 303 | 075^o^ 03. 658 | 395 |
|  | JMU-Bael (Sel-52) | Uttervehni, Samba, J&K, India | 32^o^ 39. 307 | 075^o^ 03. 667 | 397 |
|  | JMU-Bael (Sel-53) | Kartholi, Samba, J&K, India | 32^o^ 37. 591 | 074^o^ 55. 381 | 323 |
|  | JMU-Bael (Sel-54) | Kartholi, Samba, J&K, India | 32^o^ 37. 603 | 074^o^ 55. 388 | 323 |
|  | JMU-Bael (Sel-55) | Nehalki, Samba, J&K, India | 32^o^ 37. 185 | 074^o^ 56. 123 | 329 |
|  | JMU-Bael (Sel-56) | Nehalki, Samba, J&K, India | 32^o^ 37. 180 | 074^o^ 56. 135 | 328 |
|  | JMU-Bael (Sel-57) | Nehalki, Samba, J&K, India | 32^o^ 37. 098 | 074^o^ 56. 203 | 320 |
|  | JMU-Bael (Sel-58) | Taror, Samba, J&K, India | 32^o^ 36. 190 | 074^o^ 56. 878 | 310 |
|  | JMU-Bael (Sel-59) | Taror, Samba, J&K, India | 32^o^ 36. 315 | 074^o^ 56. 745 | 311 |
|  | JMU-Bael (Sel-60) | Raya, Samba, J&K, India | 32^o^ 36. 302 | 074^o^ 58. 626 | 347 |
|  | JMU-Bael (Sel-61) | Raya, Samba, J&K, India | 32^o^ 36. 304 | 074^o^ 58. 628 | 348 |
|  | JMU-Bael (Sel-62) | Sangar Parmandal, Samba, J&K, India | 32^o^ 35. 701 | 075^o^ 03. 016 | 383 |
|  | JMU-Bael (Sel-63) | Sangar Parmandal, Samba, J&K, India | 32^o^ 39. 780 | 074^o^ 03. 904 | 390 |
|  | JMU-Bael (Sel-64) | Sangar Parmandal, Samba, J&K, India | 32^o^ 39. 728 | 074^o^ 03. 949 | 393 |
|  | JMU-Bael (Sel-65) | Sangar Parmandal, Samba, J&K, India | 32^o^ 39. 804 | 074^o^ 03. 813 | 392 |
|  | JMU-Bael (Sel-66) | Sangar Parmandal, Samba, J&K, India | 32^o^ 39. 811 | 074^o^ 03. 838 | 394 |
|  | JMU-Bael (Sel-67) | Vijaypur, Samba, J&K, India | 32^o^ 33. 887 | 074^o^ 01. 412 | 331 |
|  | JMU-Bael (Sel-68) | Vijaypur, Samba, J&K, India | 32^o^ 33. 885 | 074^o^ 01. 414 | 332 |
|  | JMU-Bael (Sel-69) | Vijaypur, Samba, J&K, India | 32^o^ 33. 883 | 074^o^ 01. 417 | 334 |
|  | JMU-Bael (Sel-70) | Vijaypur, Samba, J&K, India | 32^o^ 33. 891 | 074^o^ 01. 401 | 335 |
|  | JMU-Bael (Sel-71) | Kathua, J&K, India | 32^o^ 22. 940 | 075^o^ 33. 073 | 356 |
|  | JMU-Bael (Sel-72) | Nagri Parol, Kathua , J&K, India | 32^o^ 20. 640 | 075^o^ 26. 087 | 297 |
|  | JMU-Bael (Sel-73) | Airwan, Kathua, J&K, India | 32^o^ 21. 907 | 075^o^ 24. 665 | 288 |
|  | JMU-Bael (Sel-74) | Airwan, Kathua, J&K, India | 32^o^ 21. 908 | 075^o^ 24. 663 | 289 |
|  | JMU-Bael (Sel-75) | Budhi, Kathua, J&K, India | 32^o^ 26. 277 | 075^o^ 26. 283 | 355 |
|  | JMU-Bael (Sel-76) | Budhi, Kathua, J&K, India | 32^o^ 26. 251 | 075^o^ 26. 292 | 352 |
|  | JMU-Bael (Sel-77) | Budhi, Kathua , J&K, India | 32^o^ 26. 835 | 075^o^ 26. 754 | 395 |
|  | JMU-Bael (Sel-78) | Budhi, Kathua, J&K, India | 32^o^ 26. 870 | 075^o^ 26. 727 | 394 |
|  | JMU-Bael (Sel-79) | Budhi, Kathua, J&K, India | 32^o^ 26. 988 | 075^o^ 26. 897 | 410 |
|  | JMU-Bael (Sel-80) | Mertha, Kathua, J&K, India | 32^o^ 27. 331 | 075^o^ 27. 441 | 458 |
|  | NB-5 | Raya, J&K, India | 32^o^ 36. 871 | 075^o^ 00. 182 | 411 |
|  | NB-9 | Raya, J&K, India | 32^o^ 36. 935 | 075^o^ 00. 175 | 414 |

**Table S2. Analysis of variance for 16 pomological traits of bael.**

| **Characters** | **Mean sum of squares** | | |
| --- | --- | --- | --- |
|  | **Replication (d.f.=2)** | **Treatment (d.f.=81)** | **Error (d.f.=162)** |
| Fruit length (cm) | 0.876 | 9.325** | 0.025 |
| Fruit width (cm) | 0.666 | 9.668** | 0.02 |
| Fruit weight (g) | 1,070.51 | 166326.677** | 22.161 |
| Pulp weight (g) | 1,292.68 | 92570.014** | 13.564 |
| Pulp (%) | 22.005 | 91.386** | 0.705 |
| Shell weight/fruit (g) | 160.799 | 10312.349** | 4.991 |
| Shell (%) | 23.687 | 29.797** | 0.097 |
| Fruit skull thickness (mm) | 0.065 | 0.233** | 0.006 |
| Inner diameter (cm) | 0.615 | 9.66** | 0.019 |
| Seed length (mm) | 0.054 | 5.629** | 0.022 |
| Seed diameter (mm) | 0.054 | 3.262** | 0.012 |
| Number of seed sack per fruit | 5.691 | 14.662** | 0.302 |
| Number of seeds per sack | 23.033 | 31.236** | 0.273 |
| Number of seeds per fruit | 496.984 | 4719.679** | 5.854 |
| Total seed weight per fruit | 10.489 | 259.307** | 0.645 |
| Test seed weight per fruit | 20.518 | 16.699** | 1.144 |

**Figure S1. Variations in fruit characteristics of bael genotypes.**

| 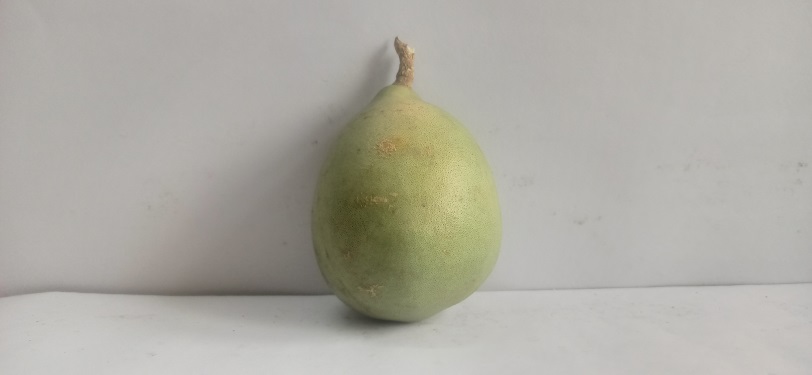 | 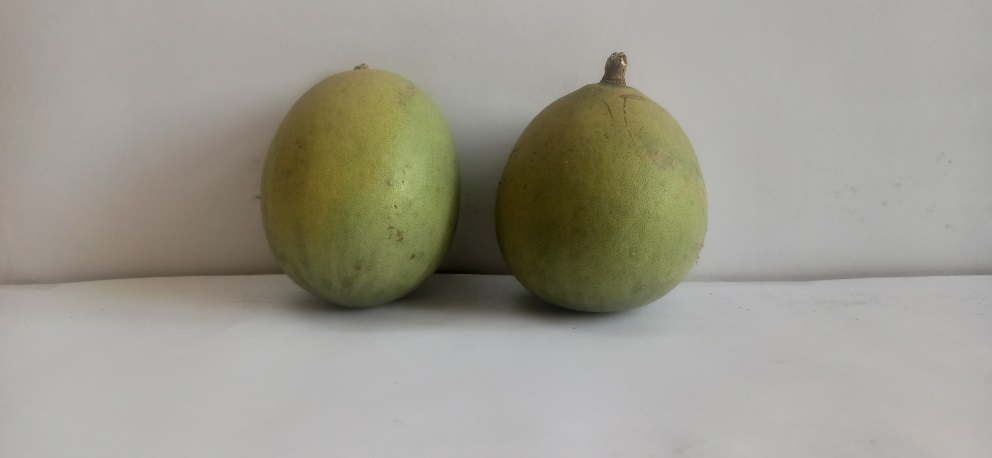 | 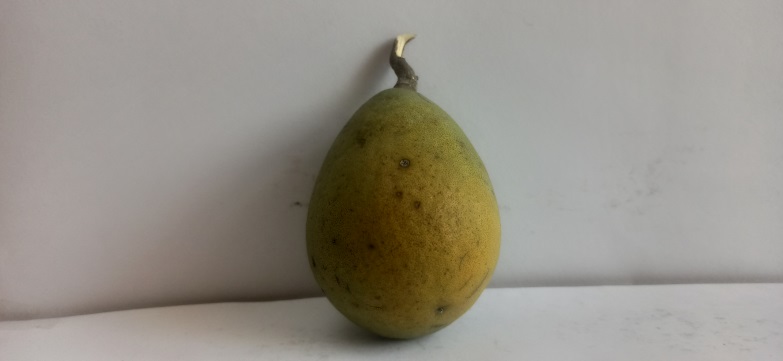 |
| --- | --- | --- |
| **JMU-Bael (Sel-1)** | **JMU-Bael (Sel-2)** | **JMU-Bael (Sel-3)** |
| 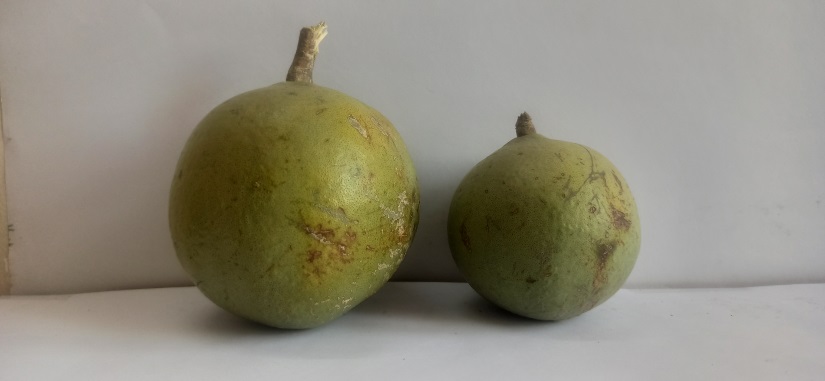 | 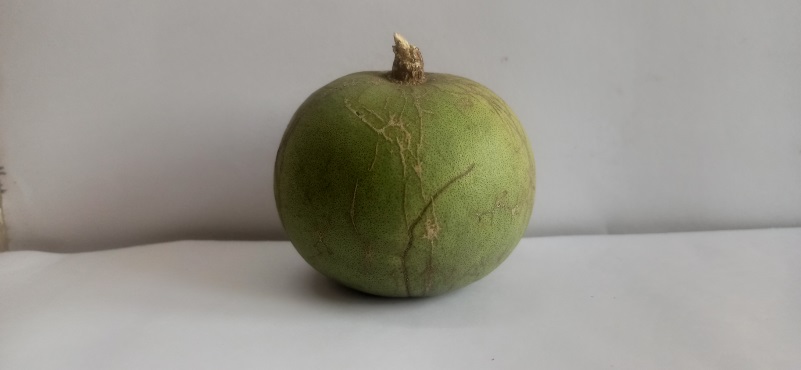 | 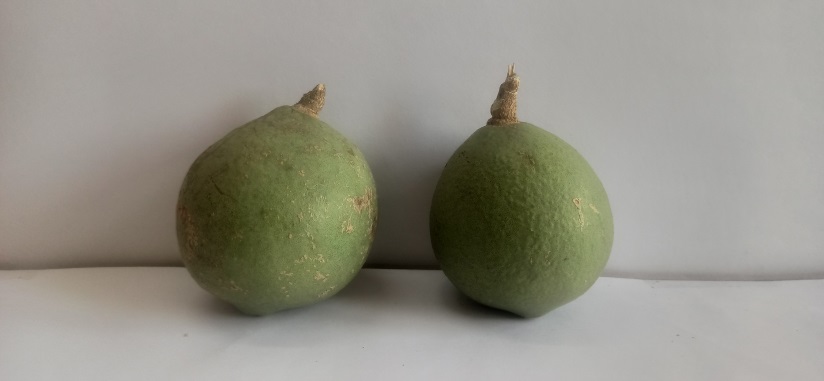 |
| **JMU-Bael (Sel-4)** | **JMU-Bael (Sel-5)** | **JMU-Bael (Sel-6)** |
| 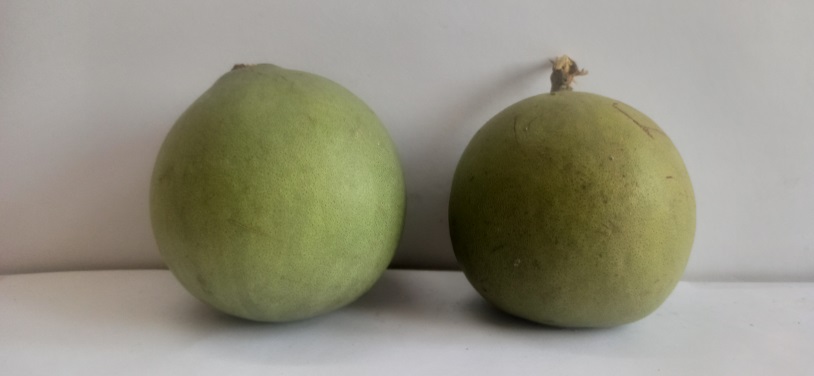 | 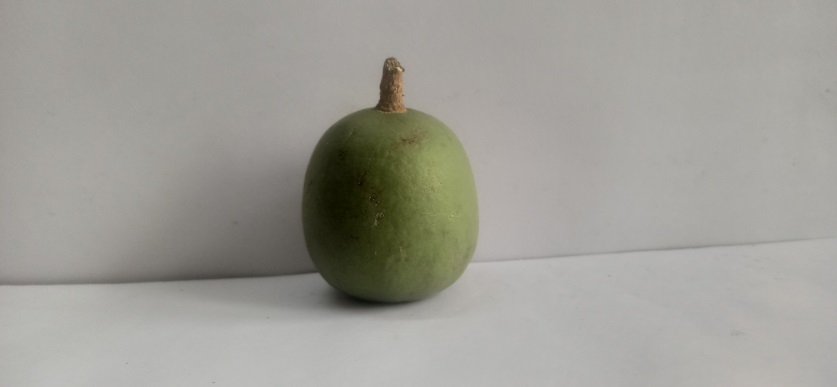 | 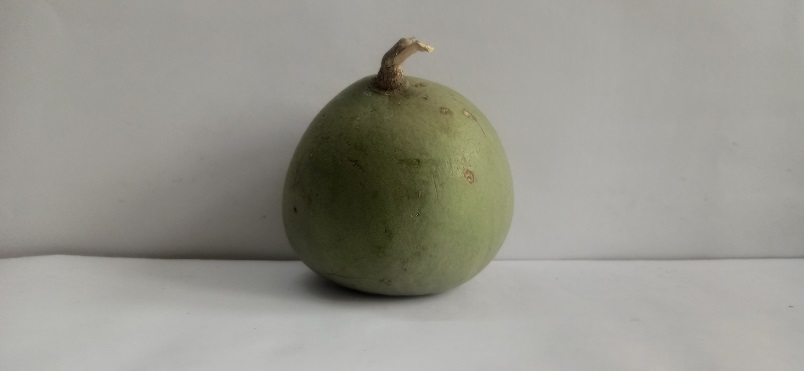 |
| **JMU-Bael (Sel-7)** | **JMU-Bael (Sel-8)** | **JMU-Bael (Sel-9)** |
| 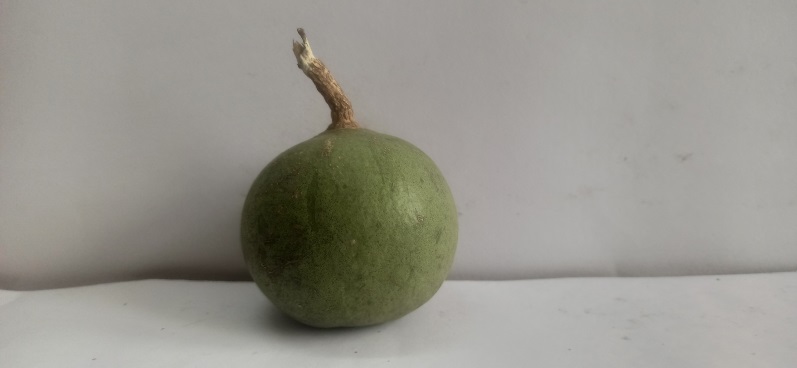 | 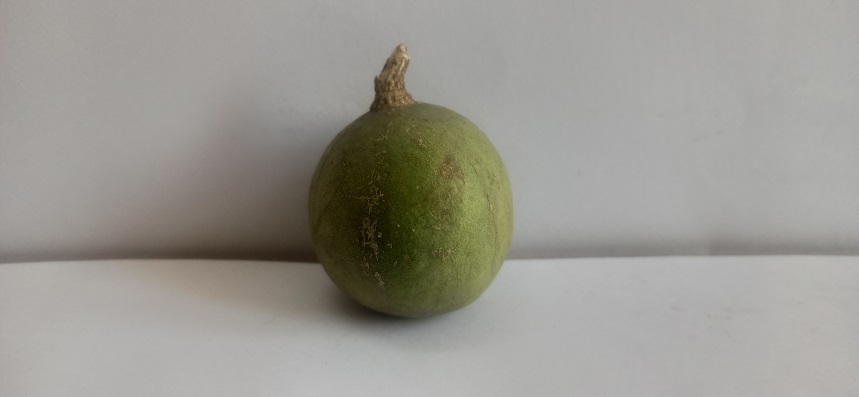 | 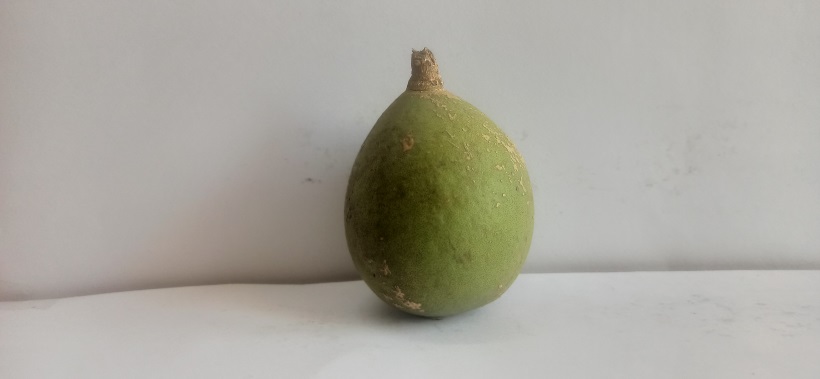 |
| **JMU-Bael (Sel-10)** | **JMU-Bael (Sel-11)** | **JMU-Bael (Sel-12)** |

| 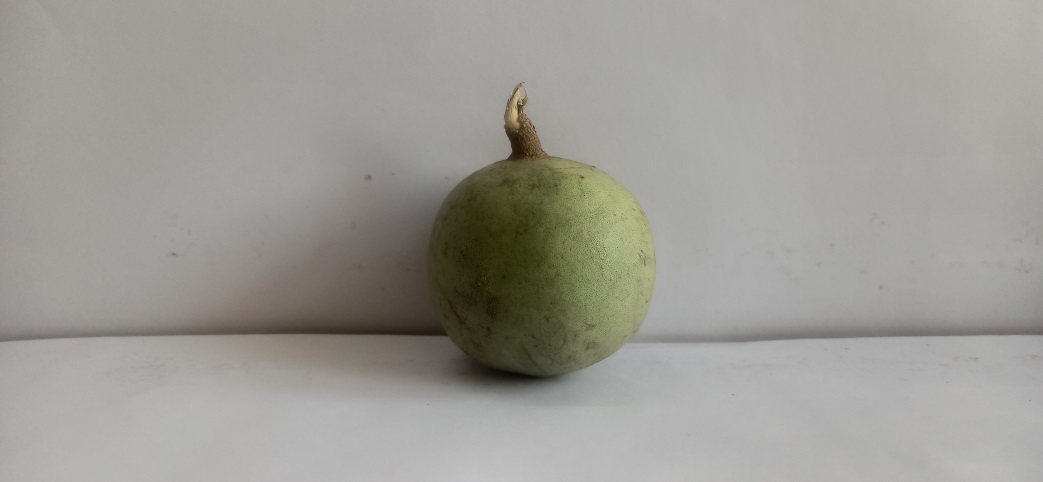 | 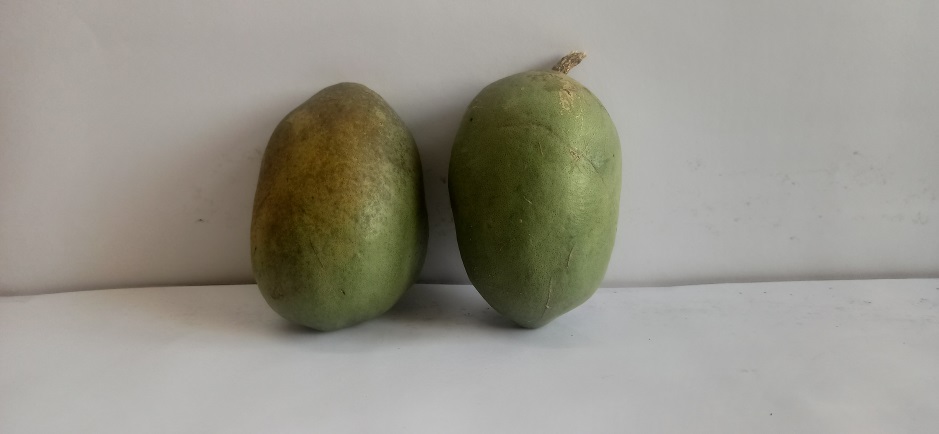 | 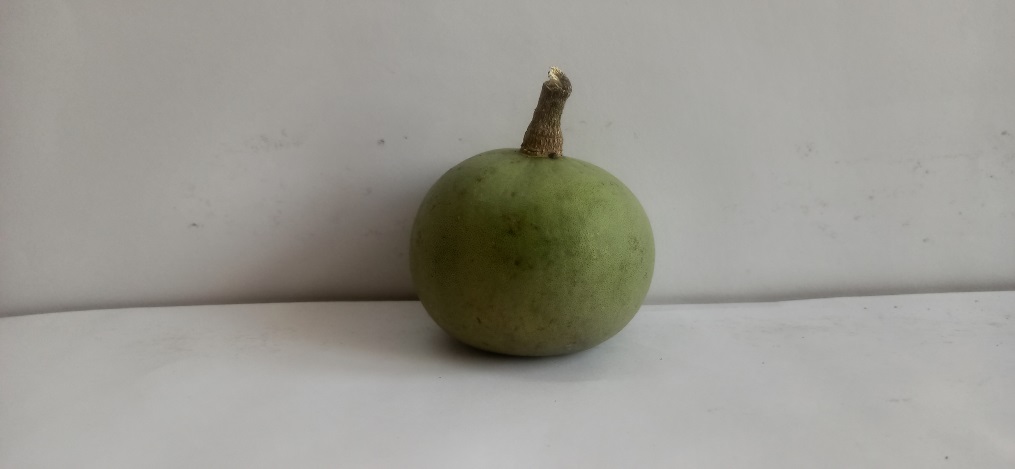 |
| --- | --- | --- |
| **JMU-Bael (Sel-13)** | **JMU-Bael (Sel-14)** | **JMU-Bael (Sel-15)** |
| 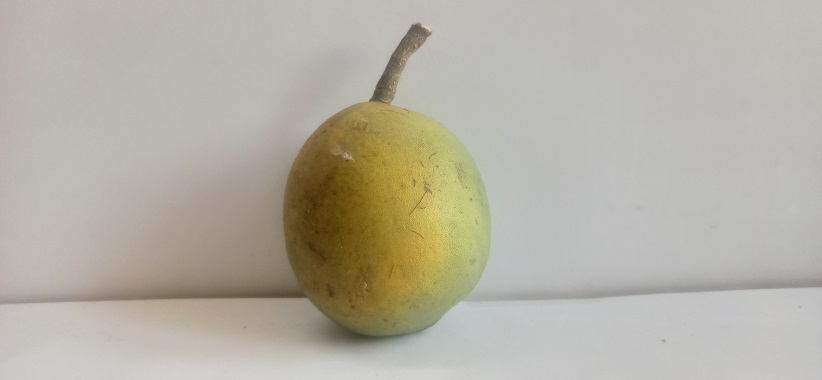 | 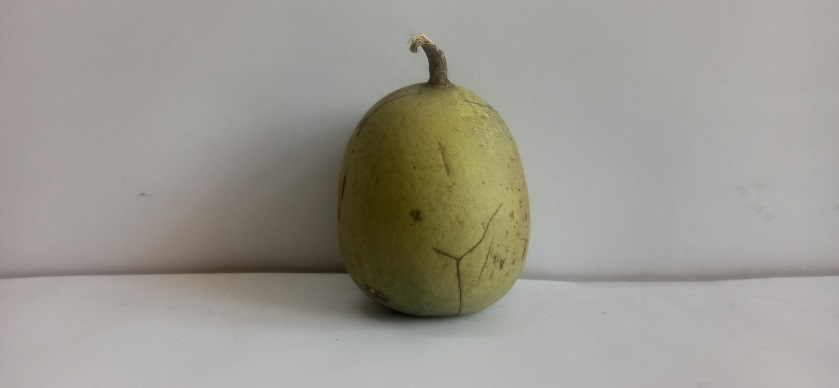 | 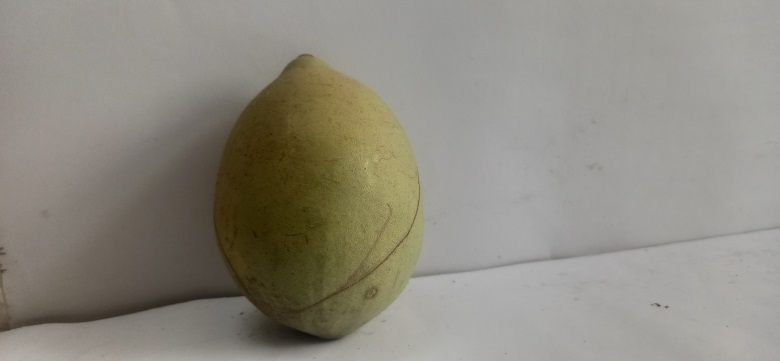 |
| **JMU-Bael (Sel-16)** | **JMU-Bael (Sel-17)** | **JMU-Bael (Sel-18)** |
| 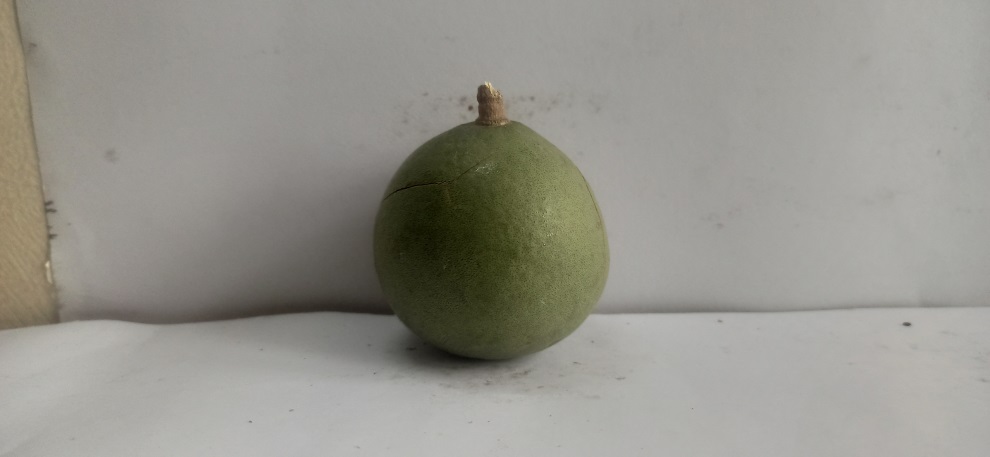 | 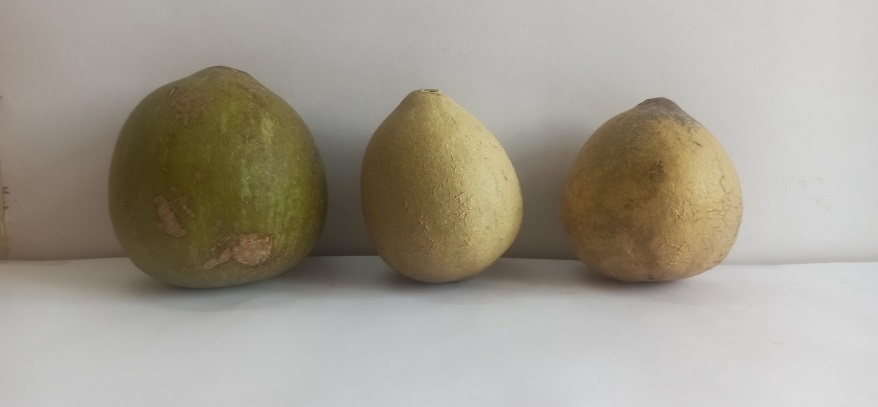 | 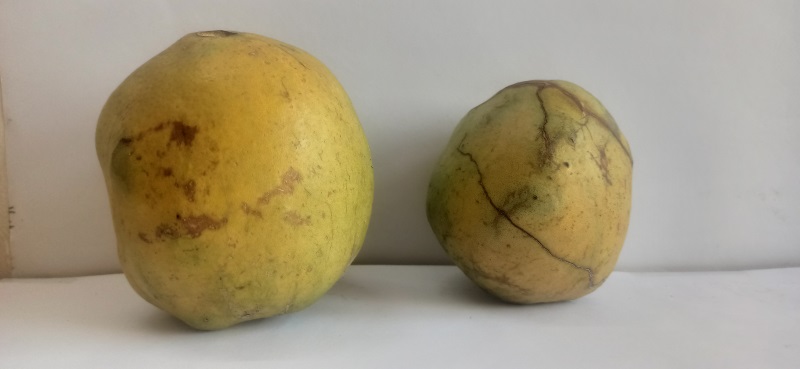 |
| **JMU-Bael (Sel-19)** | **JMU-Bael (Sel-20)** | **JMU-Bael (Sel-21)** |
| 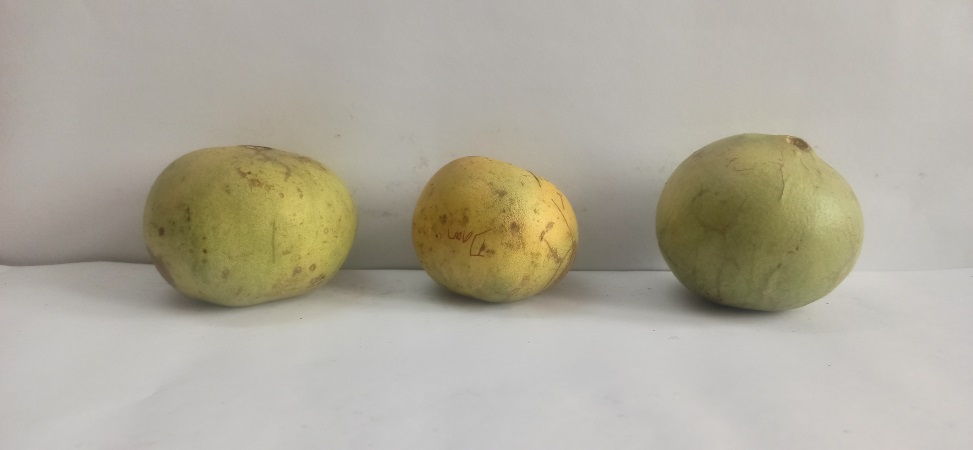 | 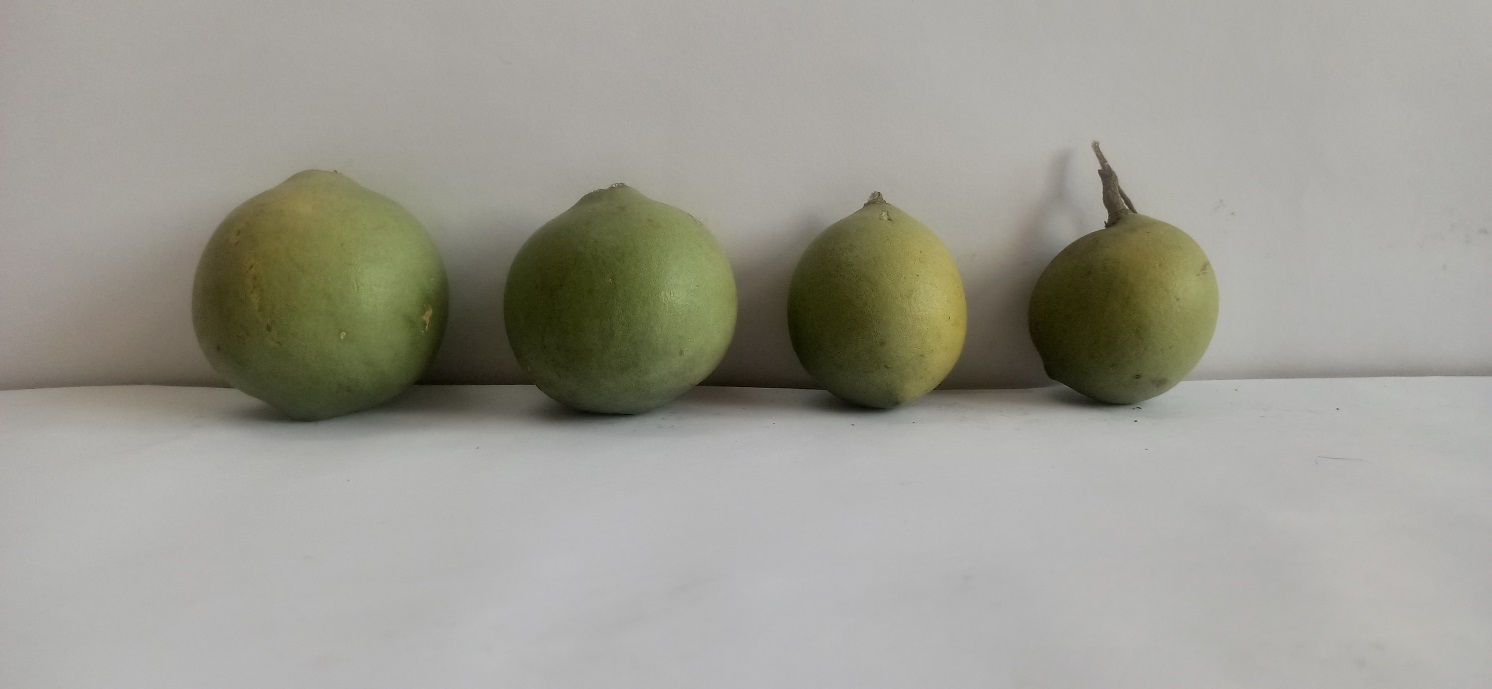 | 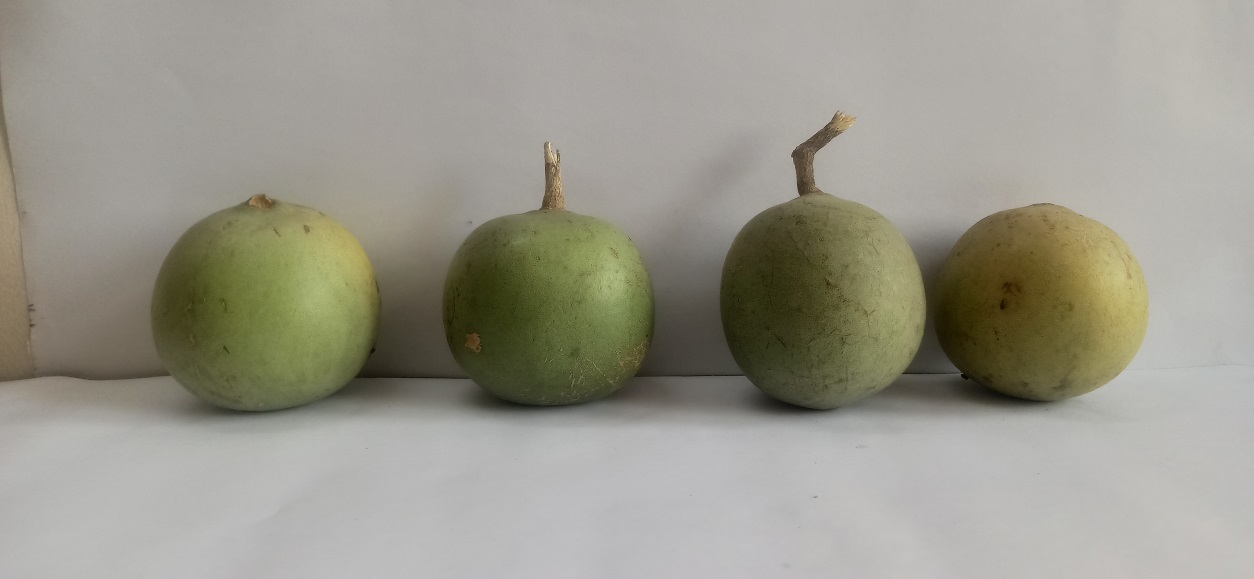 |
| **JMU-Bael (Sel-22)** | **JMU-Bael (Sel-23)** | **JMU-Bael (Sel-24)** |

| 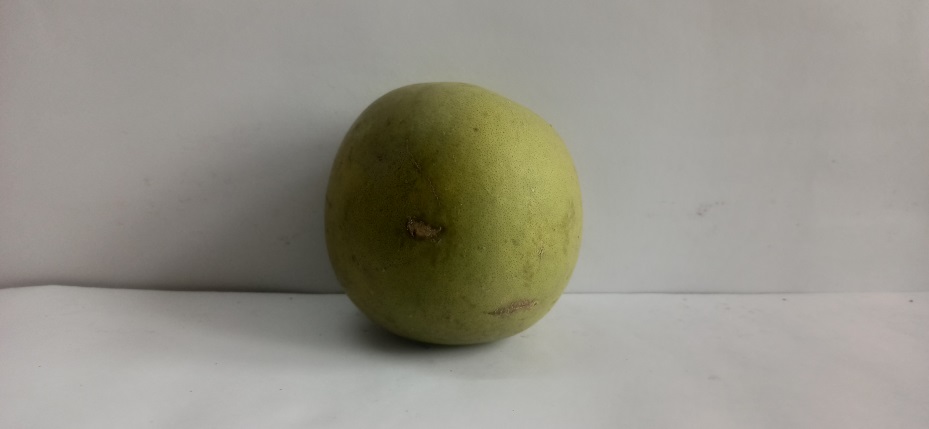 | 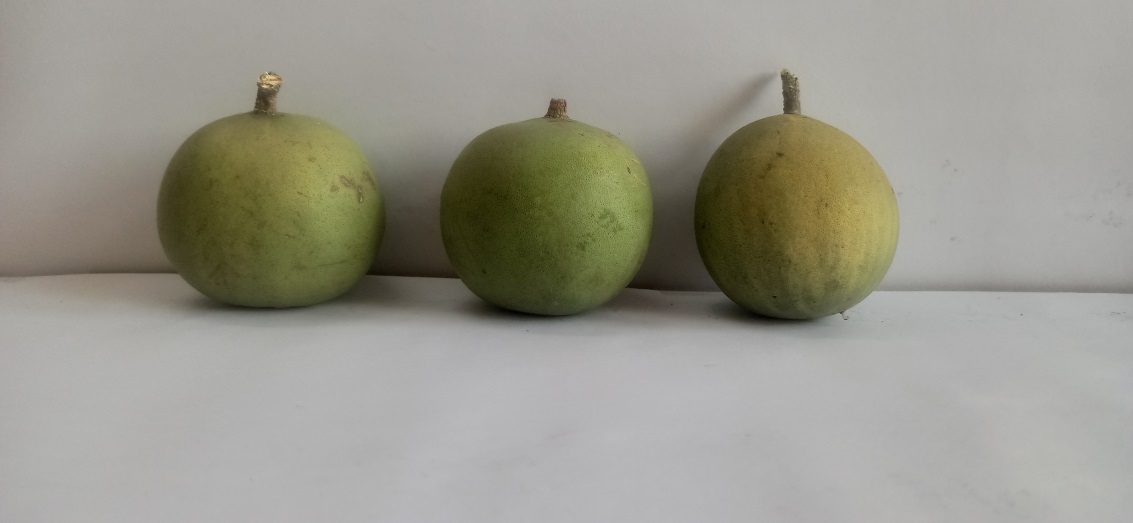 | 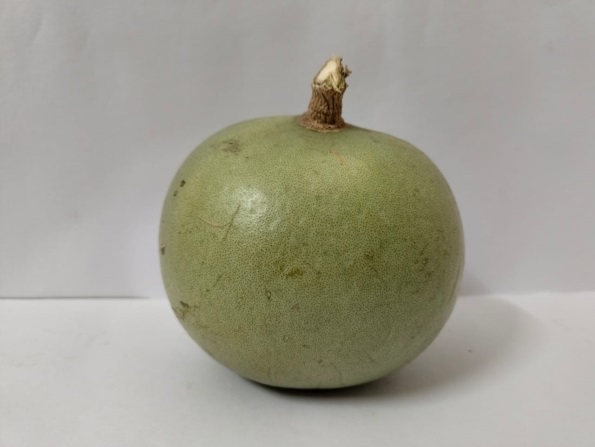 |
| --- | --- | --- |
| **JMU-Bael (Sel-25)** | **JMU-Bael (Sel-26)** | **JMU-Bael (Sel-27)** |
| 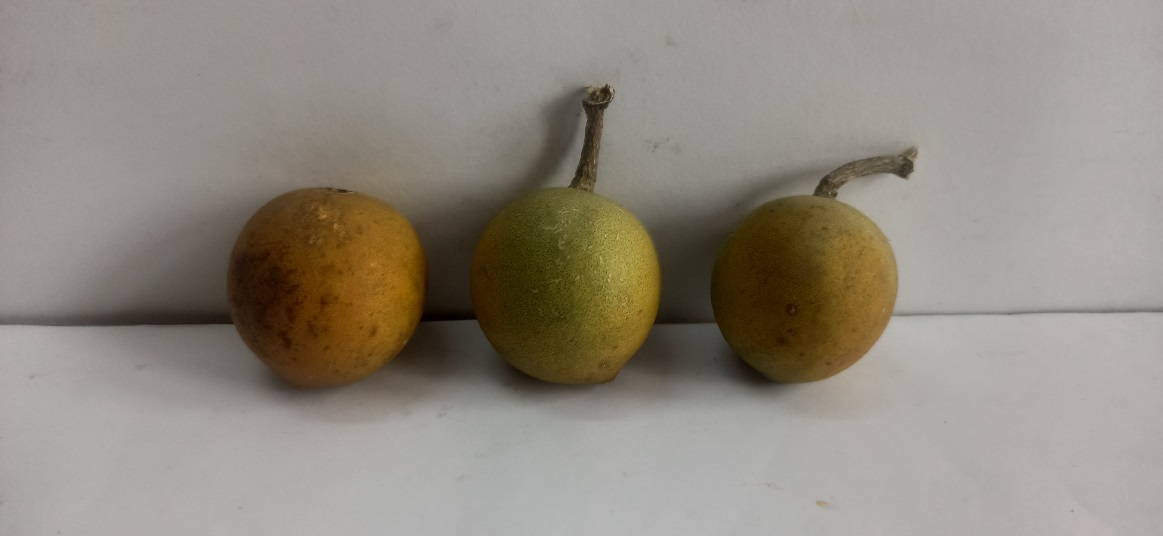 | 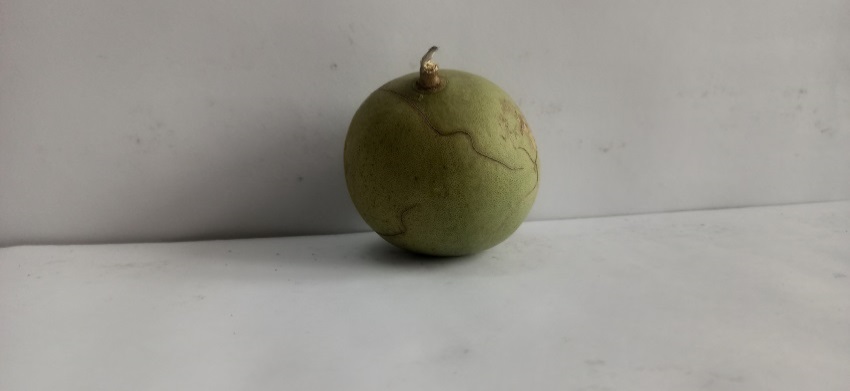 | 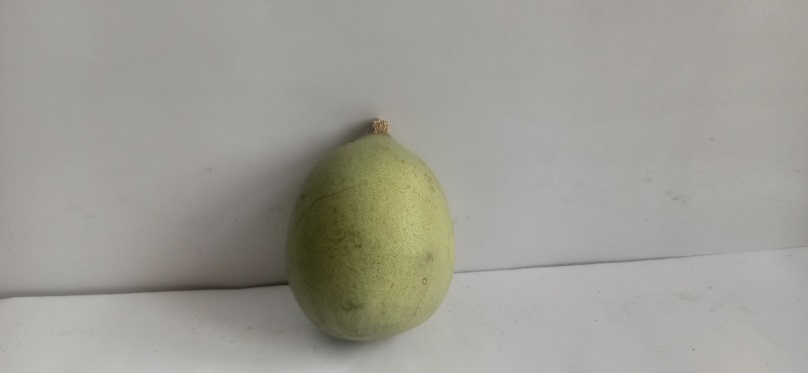 |
| **JMU-Bael (Sel-28)** | **JMU-Bael (Sel-29)** | **JMU-Bael (Sel-30)** |
| 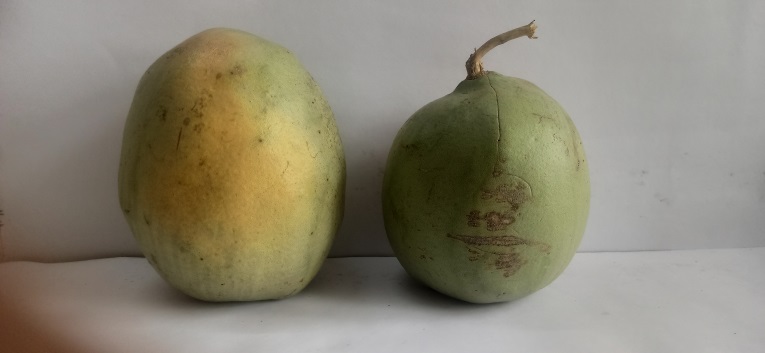 | 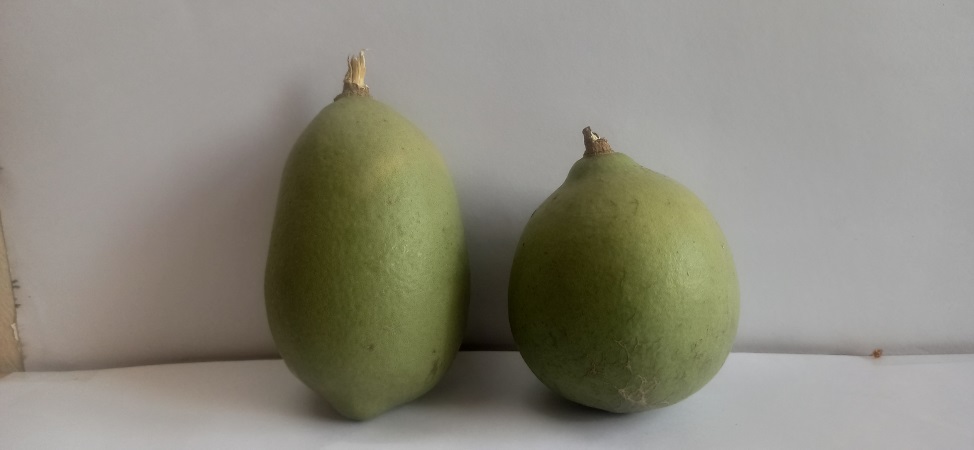 | 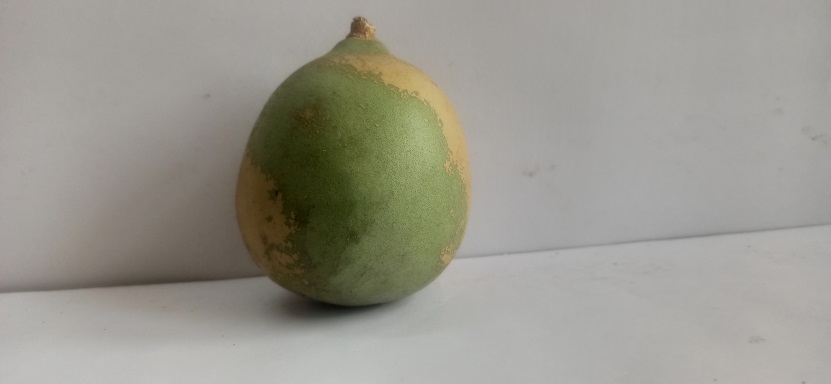 |
| **JMU-Bael (Sel-31)** | **JMU-Bael (Sel-32)** | **JMU-Bael (Sel-33)** |
| 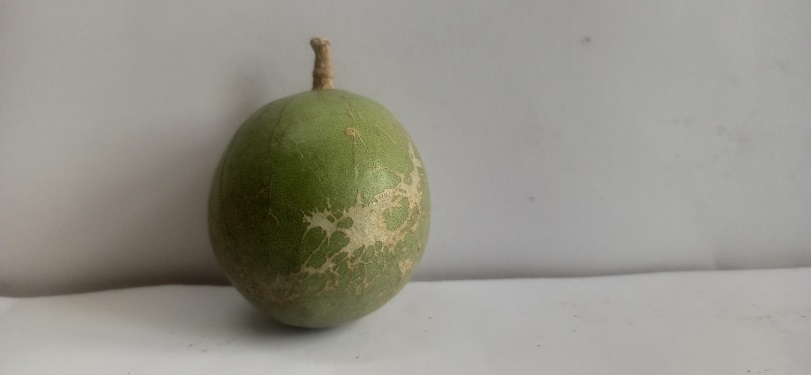 | 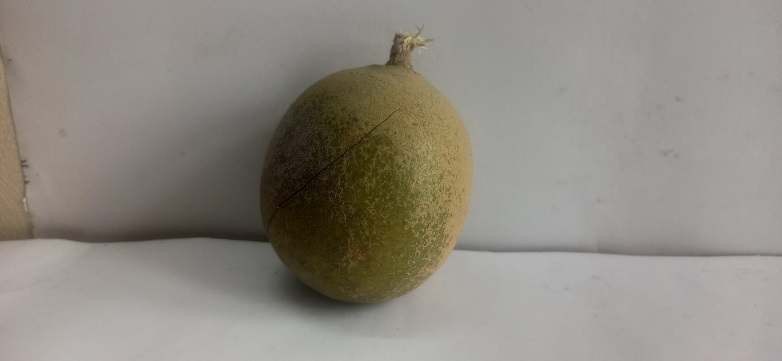 | 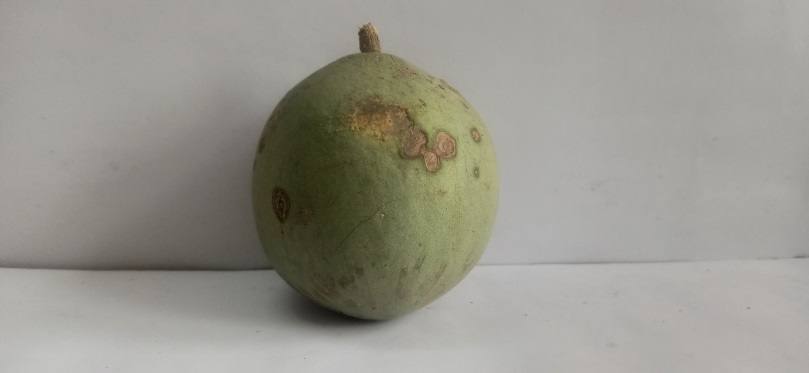 |
| **JMU-Bael (Sel-34)** | **JMU-Bael (Sel-35)** | **JMU-Bael (Sel-36)** |

| 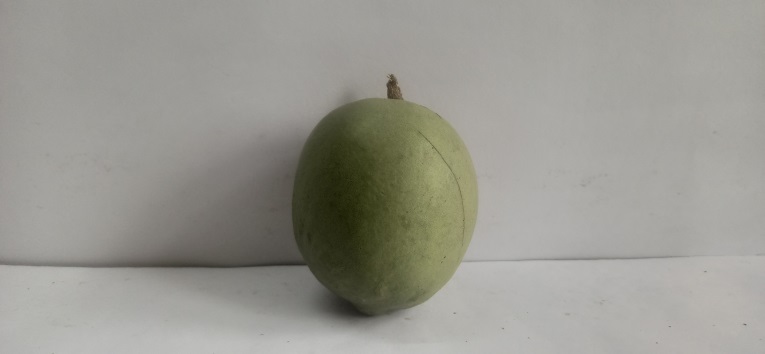 | 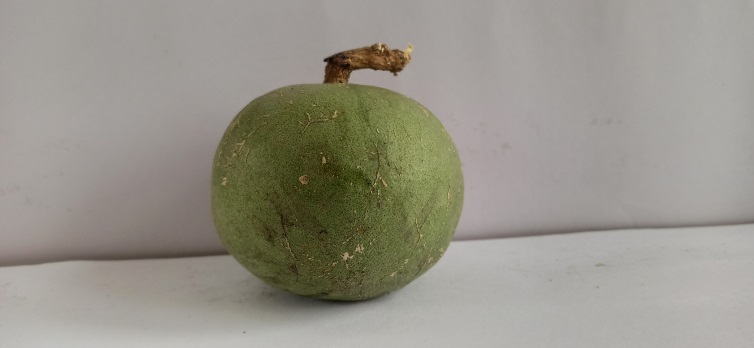 | 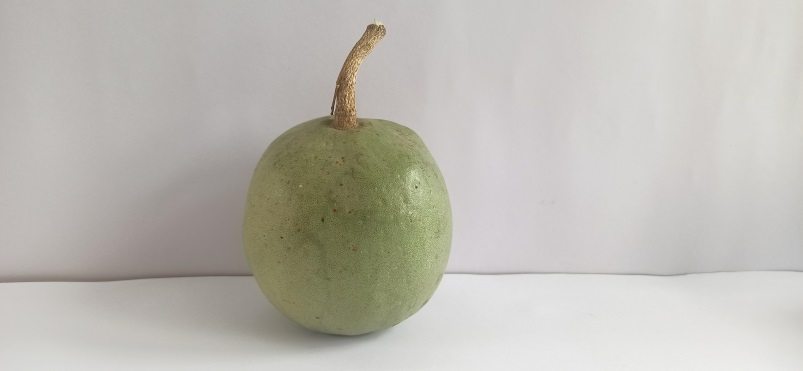 |
| --- | --- | --- |
| **JMU-Bael (Sel-37)** | **JMU-Bael (Sel-38)** | **JMU-Bael (Sel-39)** |
| 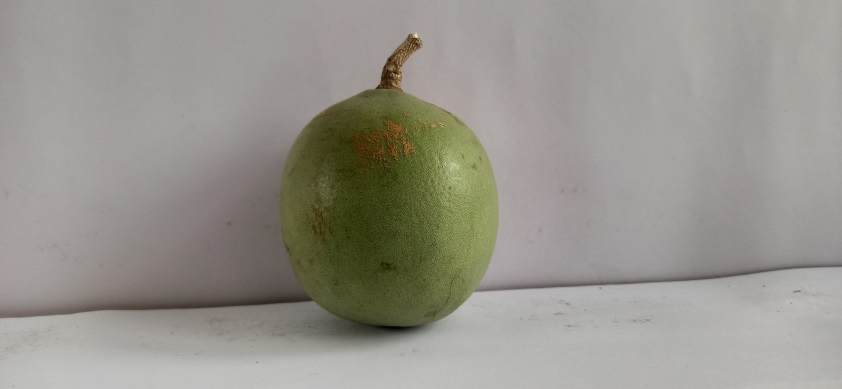 | 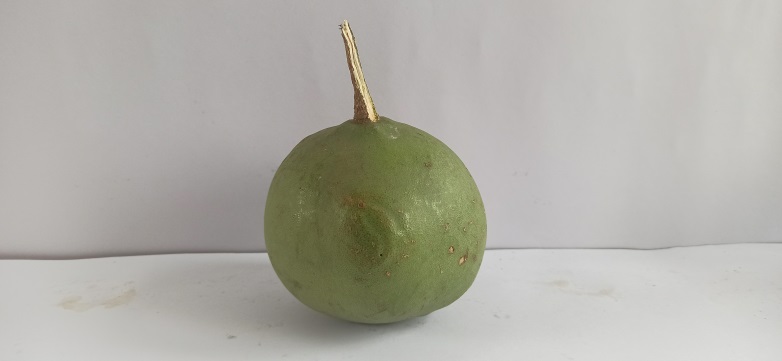 | 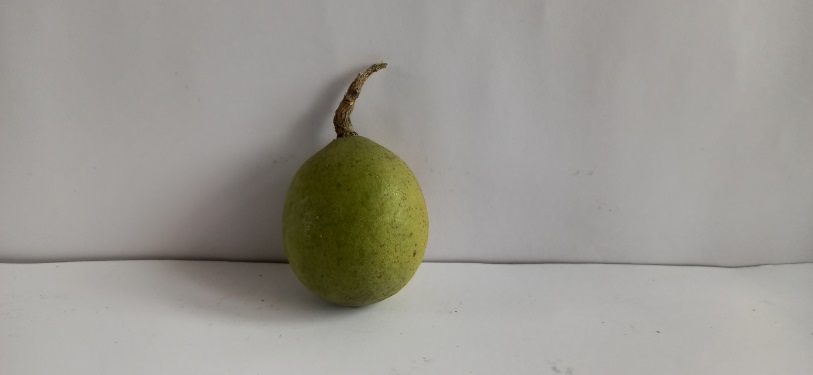 |
| **JMU-Bael (Sel-40)** | **JMU-Bael (Sel-41)** | **JMU-Bael (Sel-42)** |
| 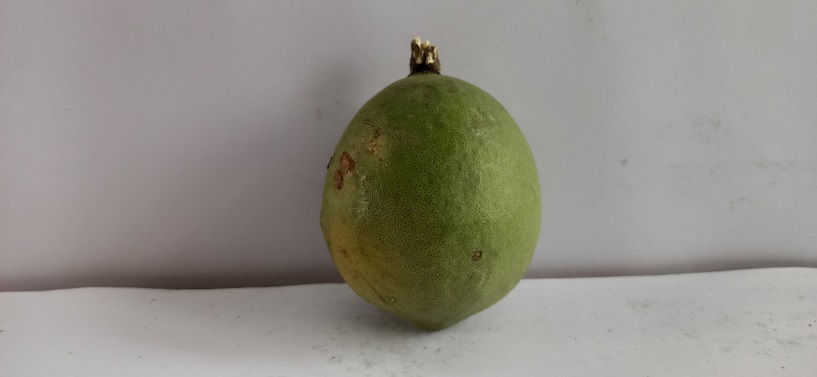 | 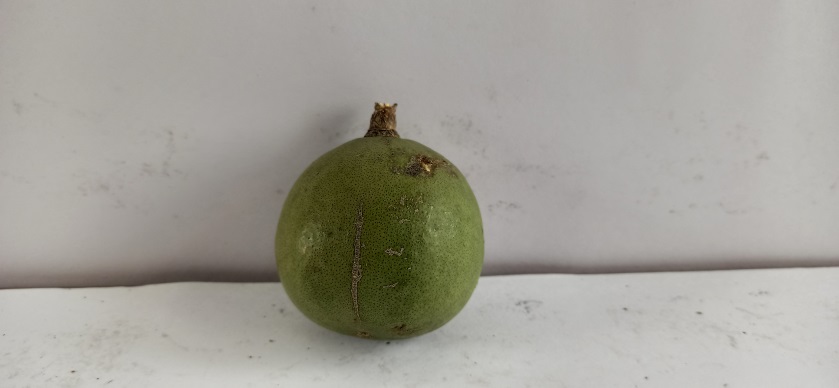 | 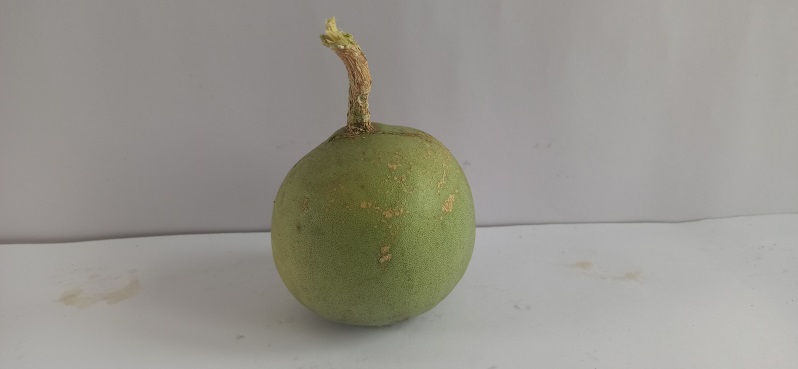 |
| **JMU-Bael (Sel-43)** | **JMU-Bael (Sel-44)** | **JMU-Bael (Sel-45)** |
| 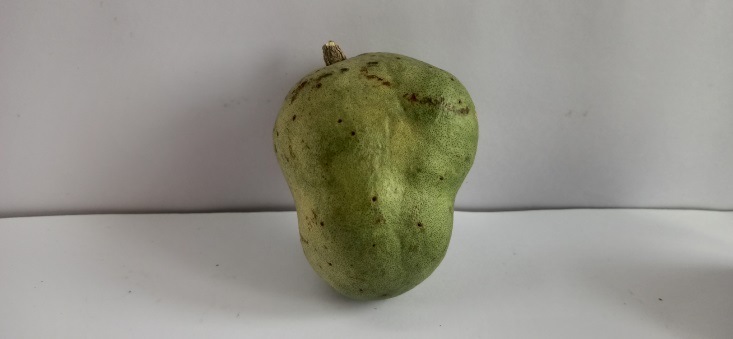 | 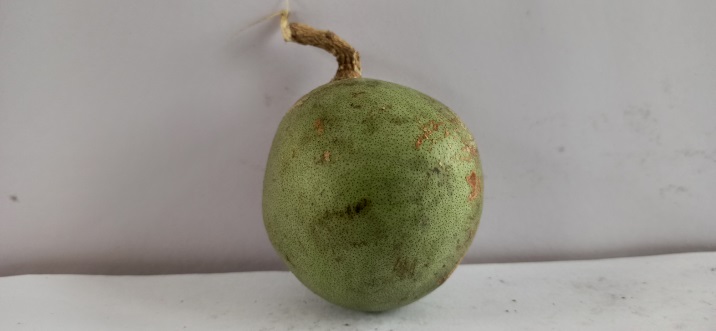 | 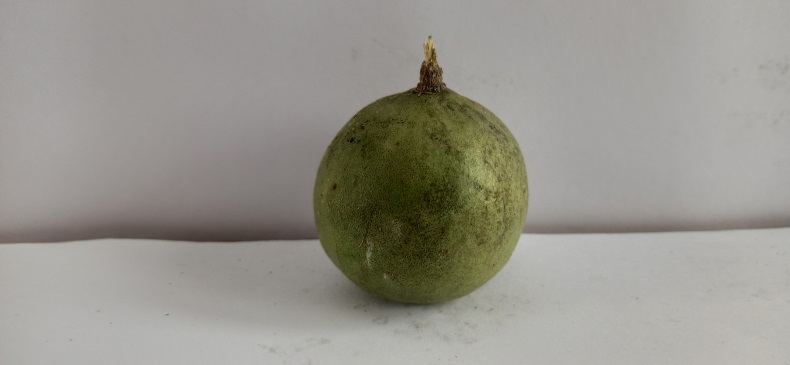 |
| **JMU-Bael (Sel-46)** | **JMU-Bael (Sel-47)** | **JMU-Bael (Sel-48)** |

| 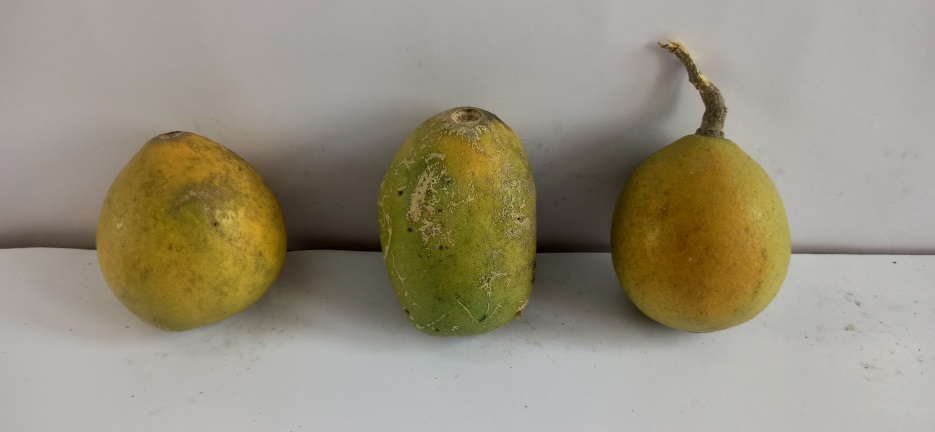 | 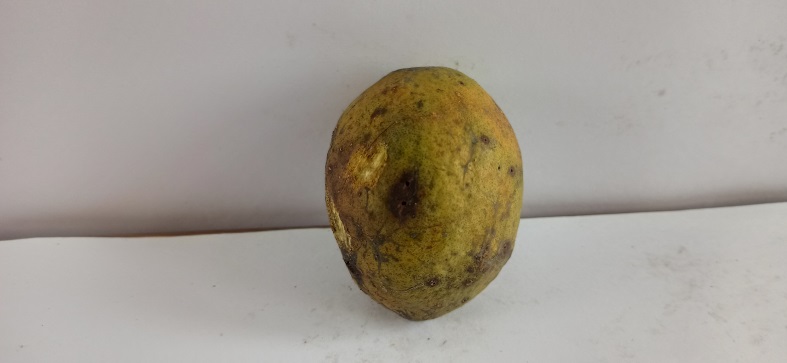 | 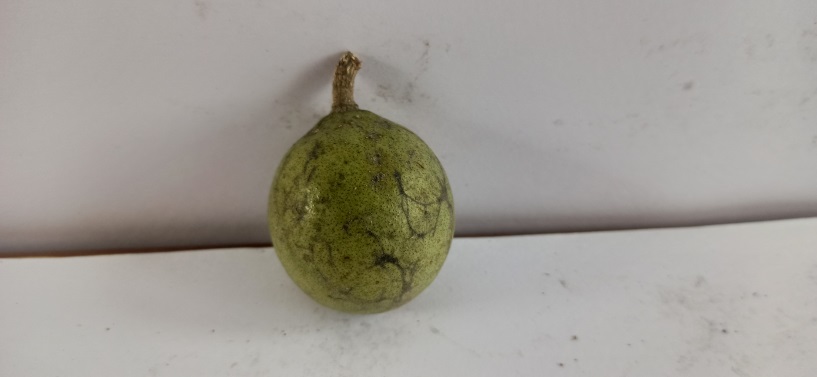 |
| --- | --- | --- |
| **JMU-Bael (Sel-49)** | **JMU-Bael (Sel-50)** | **JMU-Bael (Sel-51)** |
| 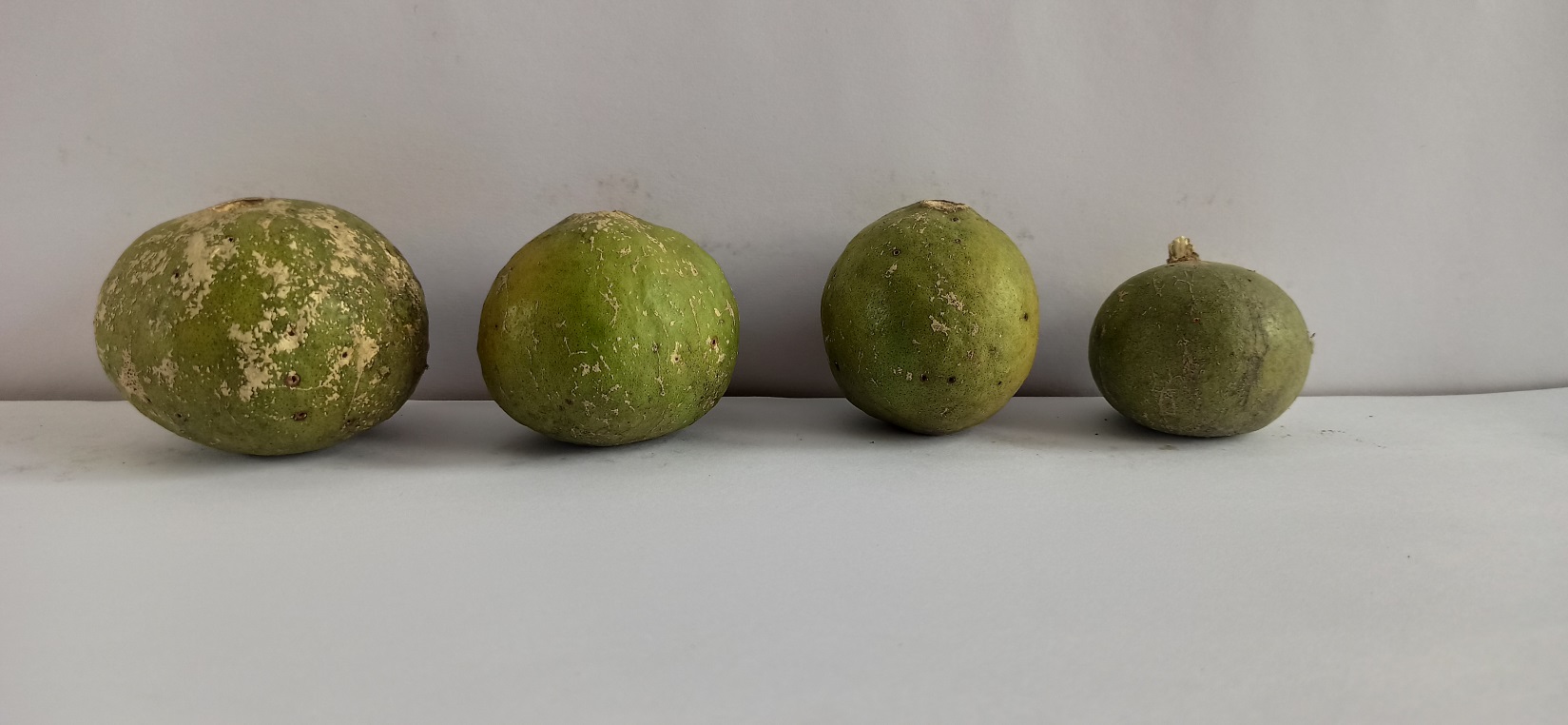 | 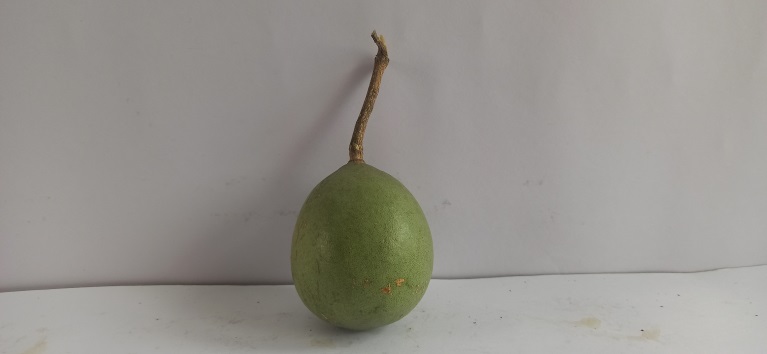 | 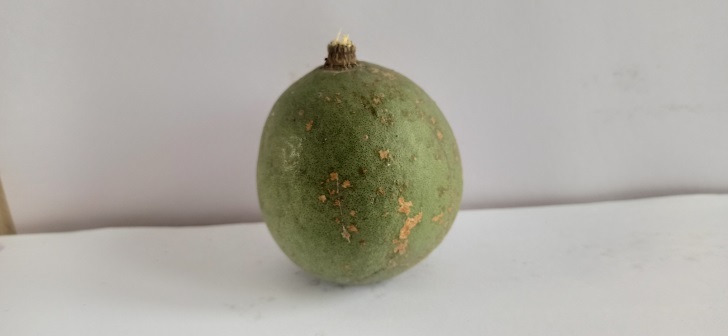 |
| **JMU-Bael (Sel-52)** | **JMU-Bael (Sel-53)** | **JMU-Bael (Sel-54)** |
| 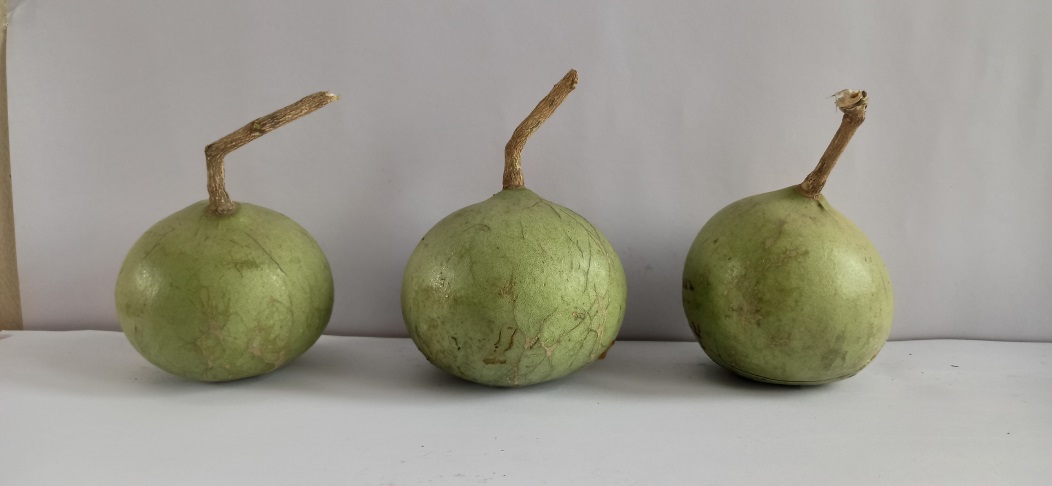 | 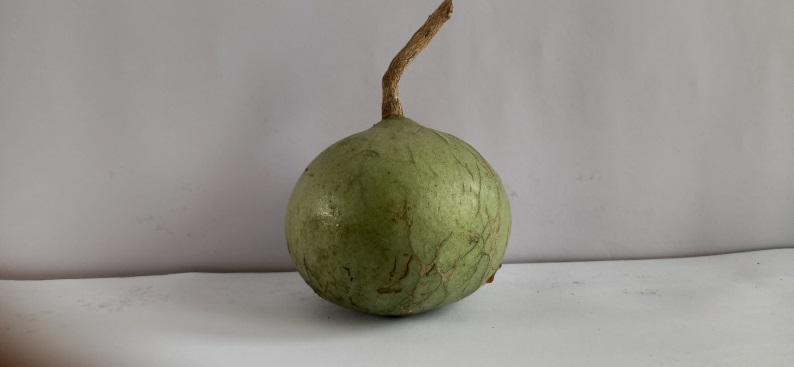 | 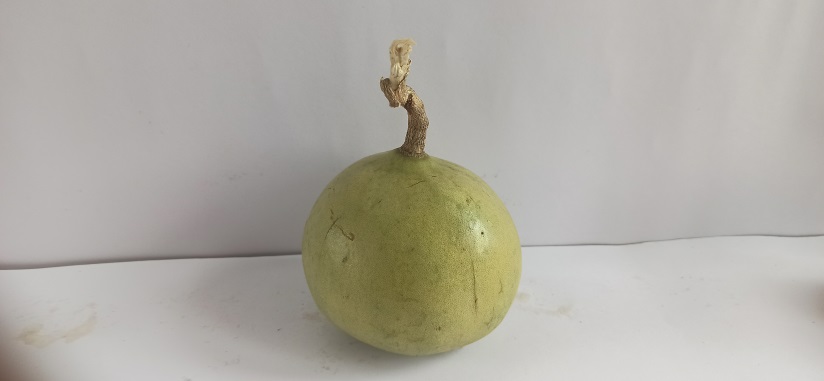 |
| **JMU-Bael (Sel-55)** | **JMU-Bael (Sel-56)** | **JMU-Bael (Sel-57)** |
| 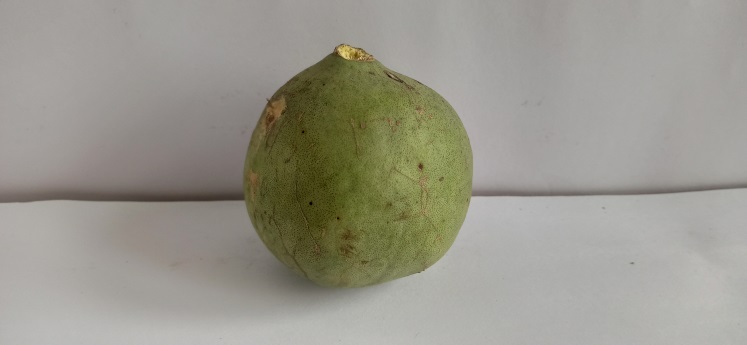 | 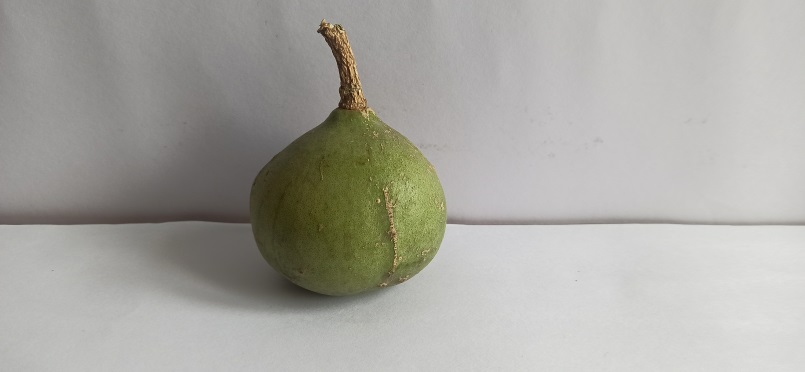 | 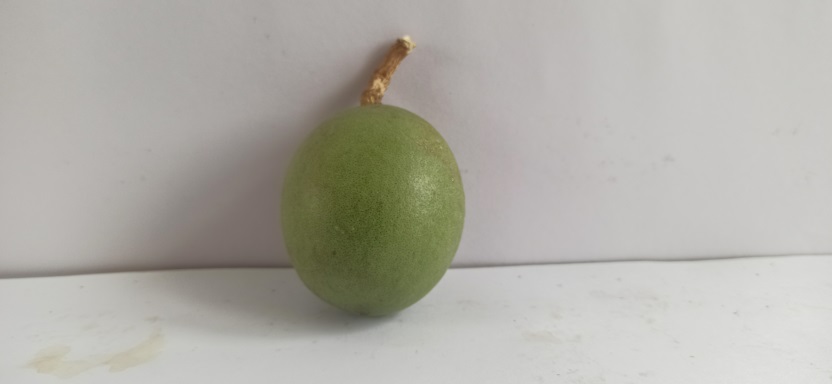 |
| **JMU-Bael (Sel-58)** | **JMU-Bael (Sel-59)** | **JMU-Bael (Sel-60)** |

| 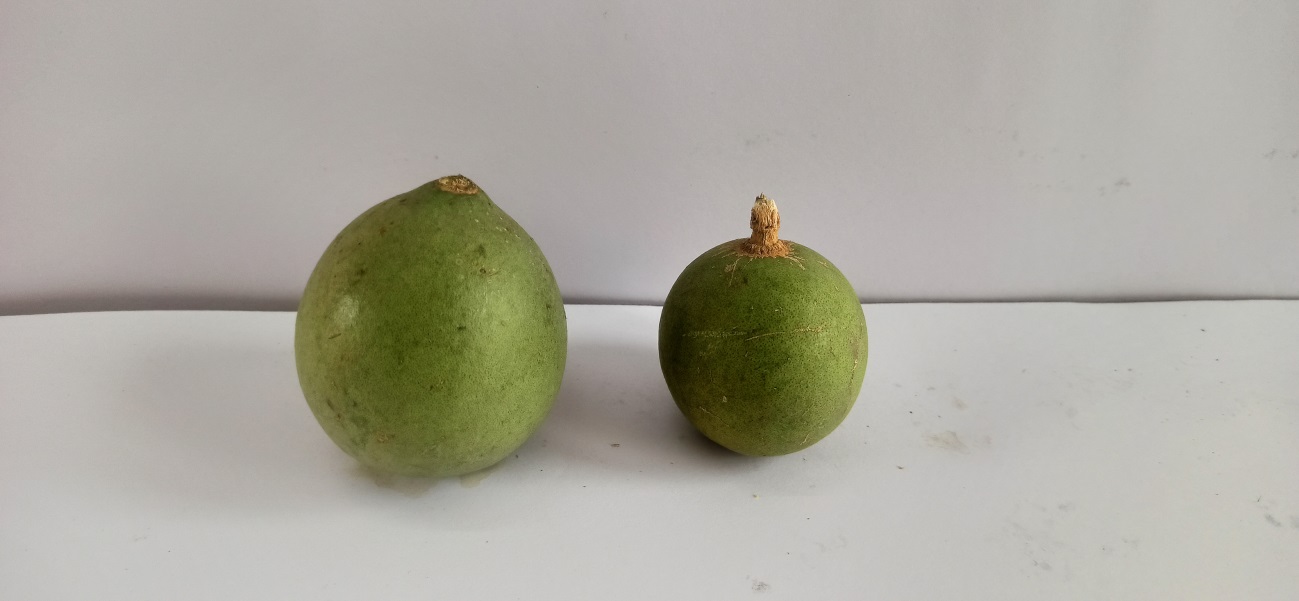 | 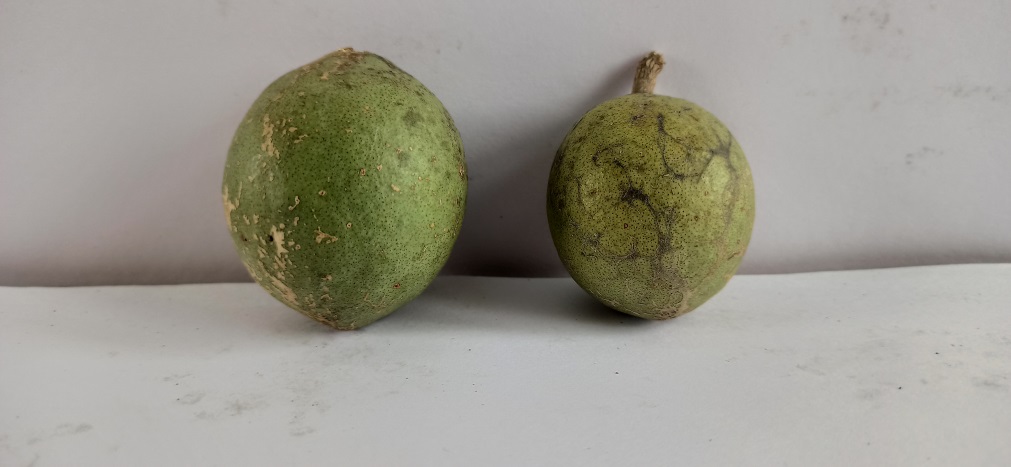 | 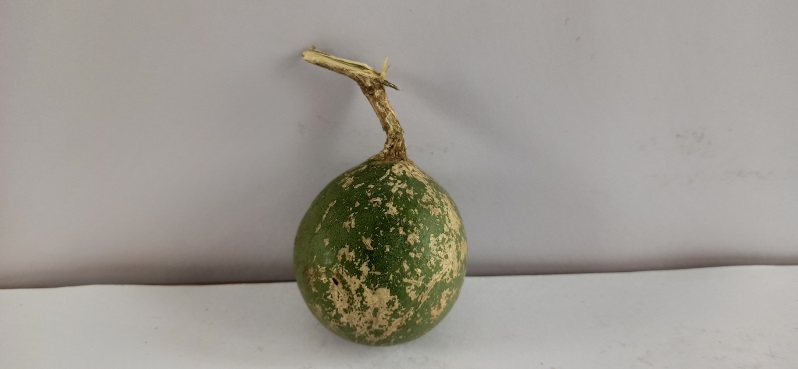 |
| --- | --- | --- |
| **JMU-Bael (Sel-61)** | **JMU-Bael (Sel-62)** | **JMU-Bael (Sel-63)** |
| 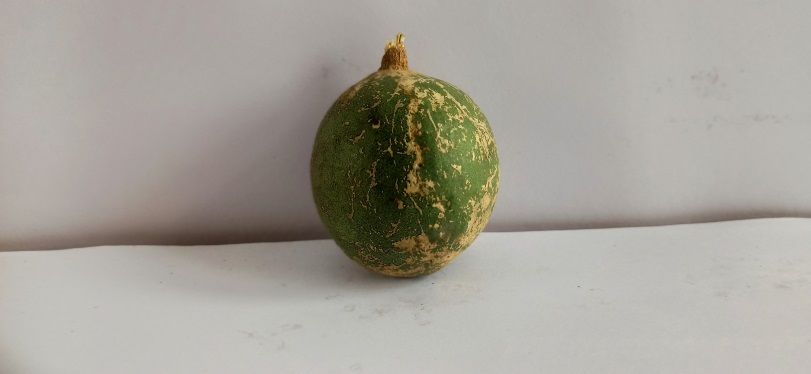 | 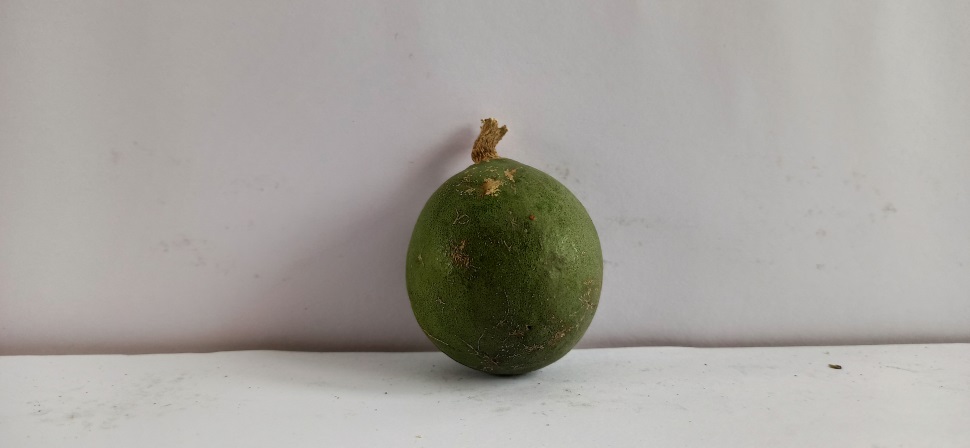 | 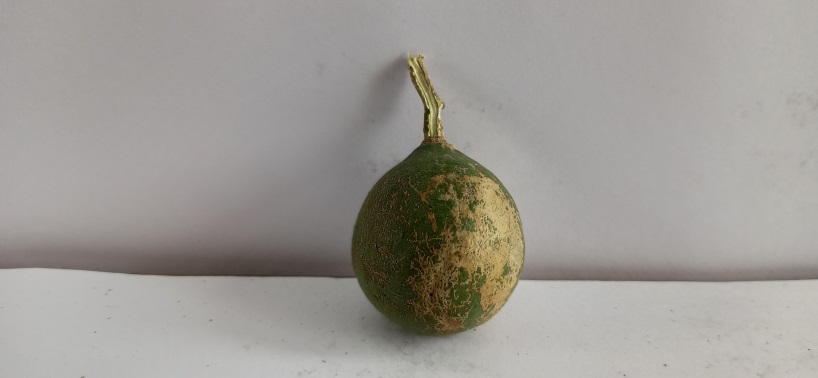 |
| **JMU-Bael (Sel-64)** | **JMU-Bael (Sel-65)** | **JMU-Bael (Sel-66)** |
| 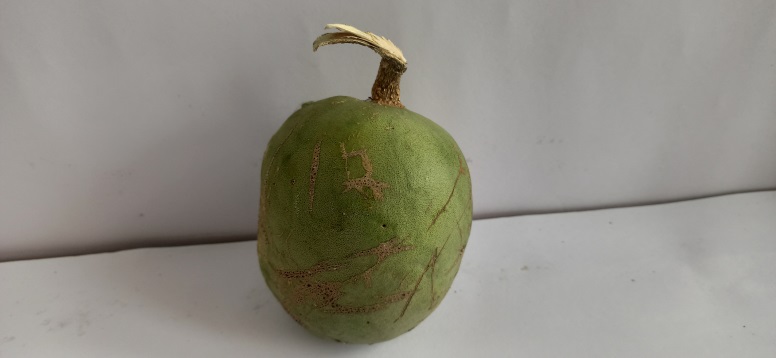 | 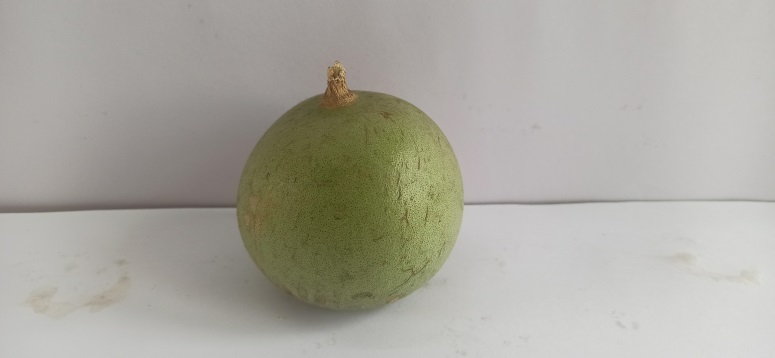 | 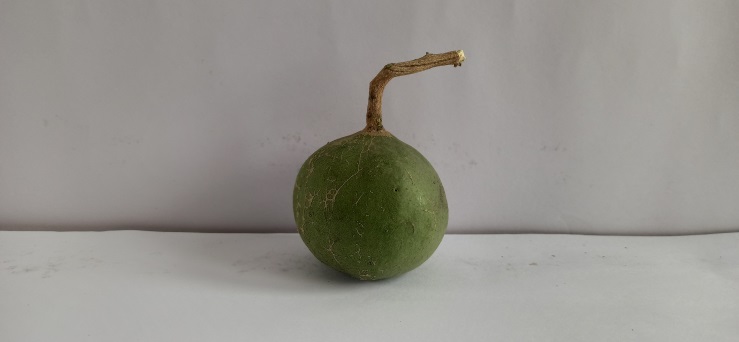 |
| **JMU-Bael (Sel-67)** | **JMU-Bael (Sel-68)** | **JMU-Bael (Sel-69)** |
| 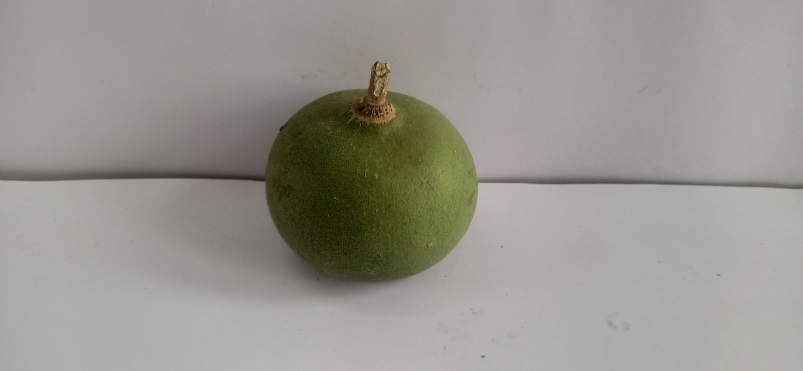 | 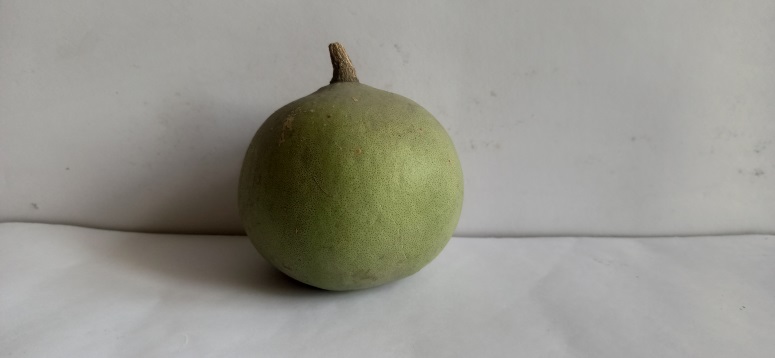 | 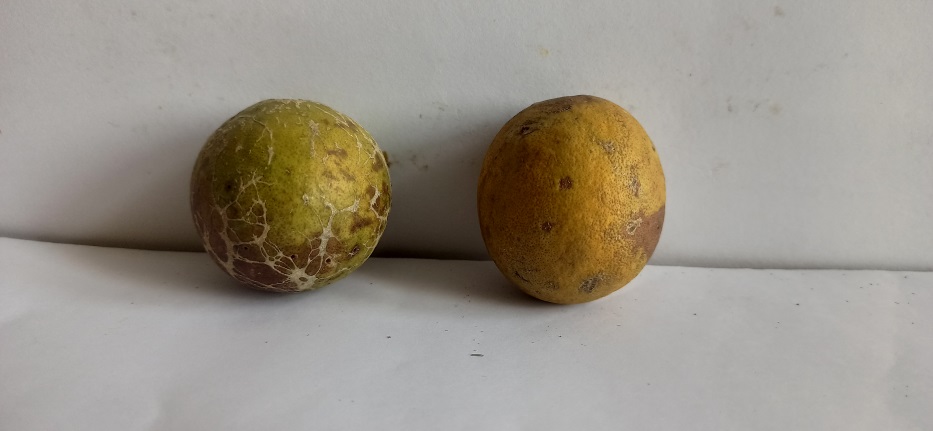 |
| **JMU-Bael (Sel-70)** | **JMU-Bael (Sel-71)** | **JMU-Bael (Sel-72)** |

| 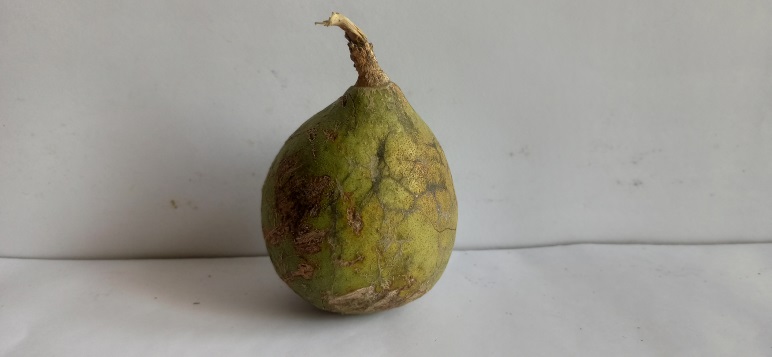 | 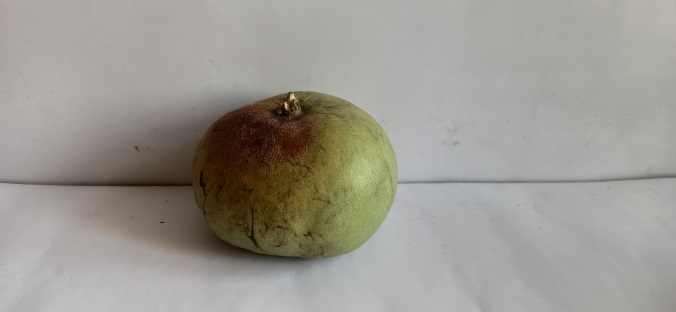 | 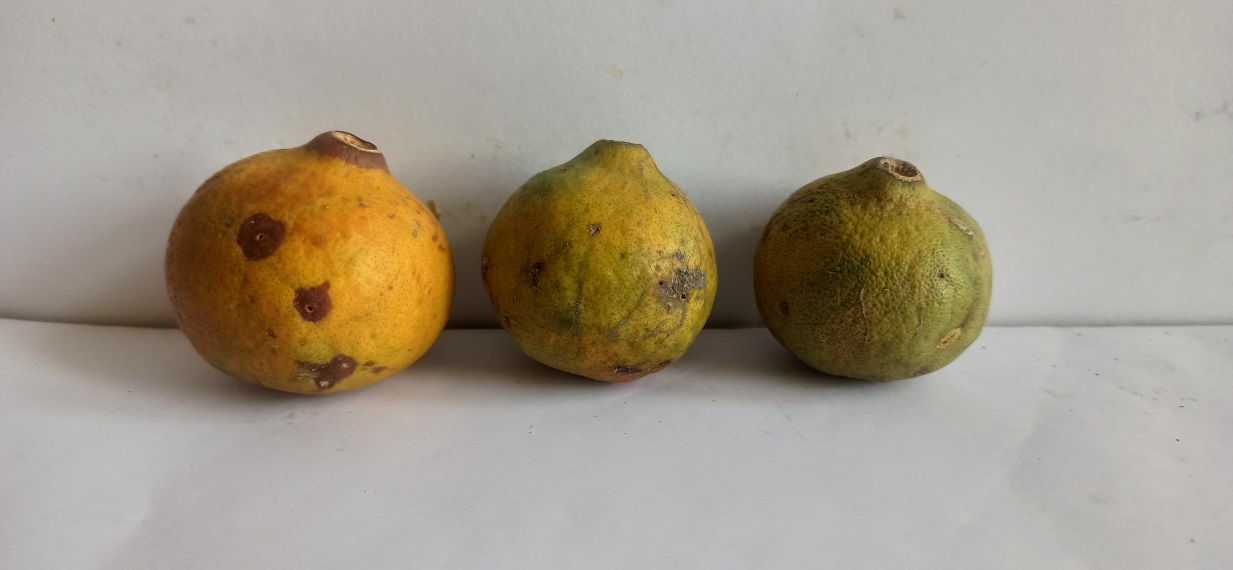 |
| --- | --- | --- |
| **JMU-Bael (Sel-73)** | **JMU-Bael (Sel-74)** | **JMU-Bael (Sel-75)** |
| 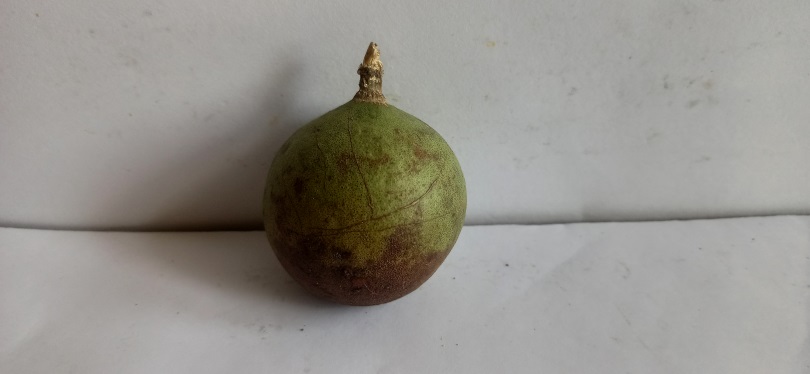 | 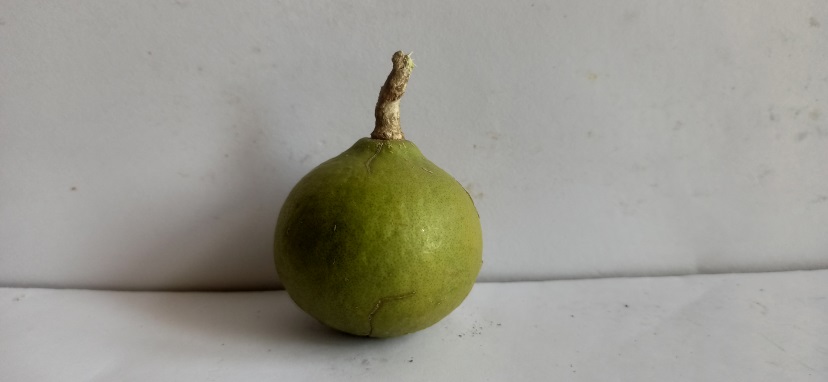 | 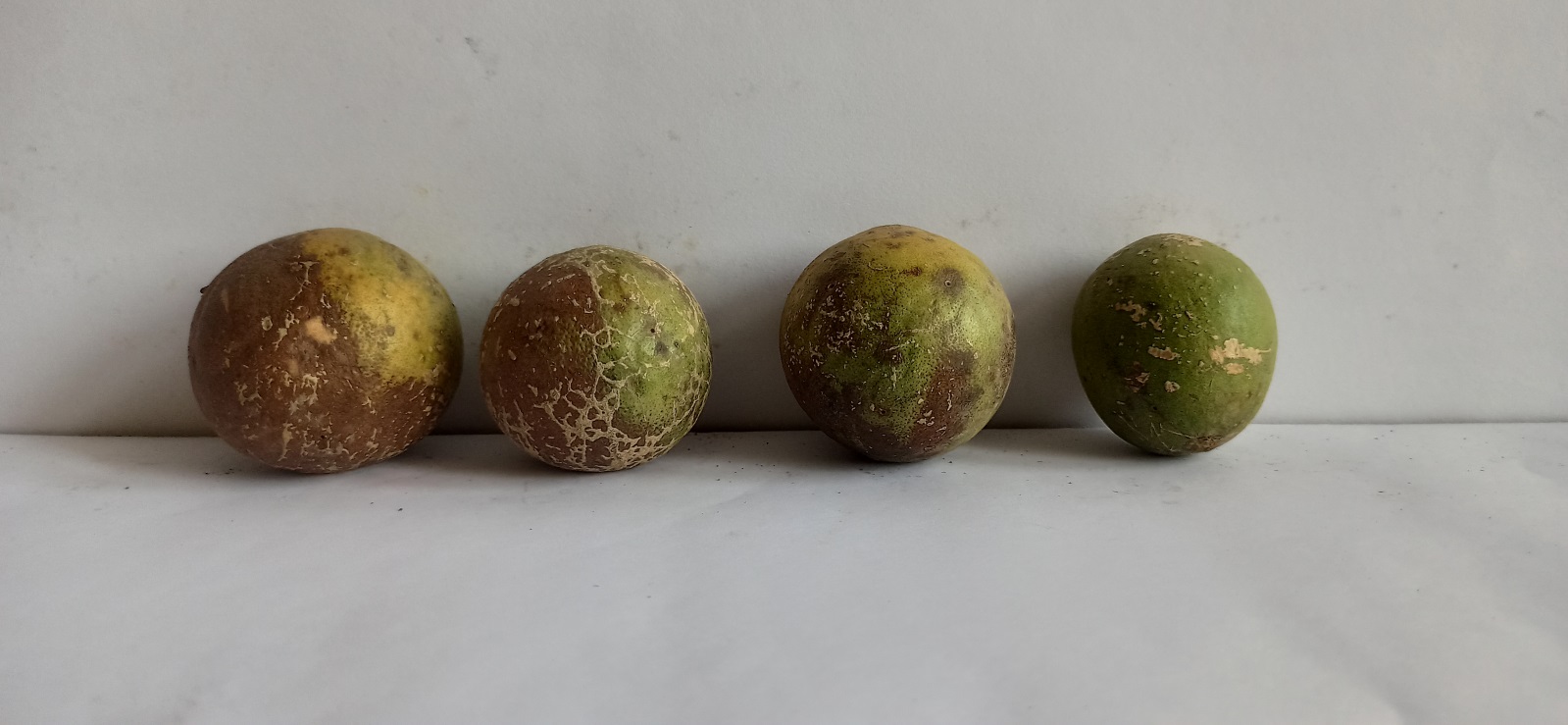 |
| **JMU-Bael (Sel-76)** | **JMU-Bael (Sel-77)** | **JMU-Bael (Sel-78)** |
| 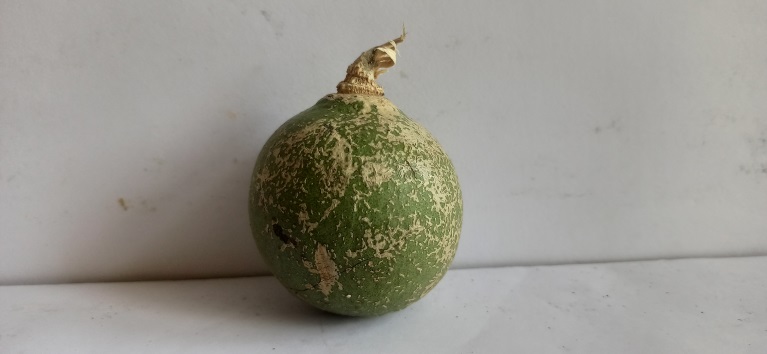 | 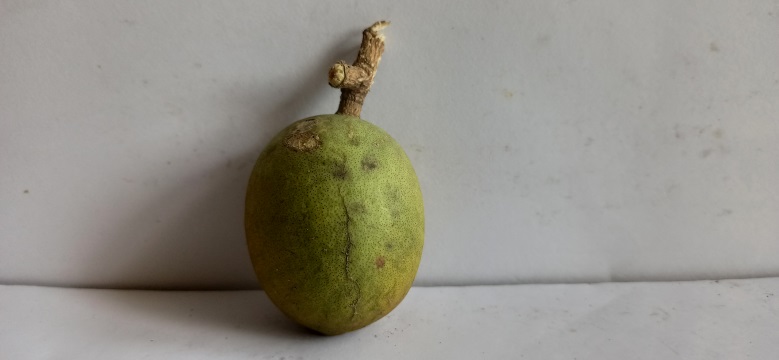 | 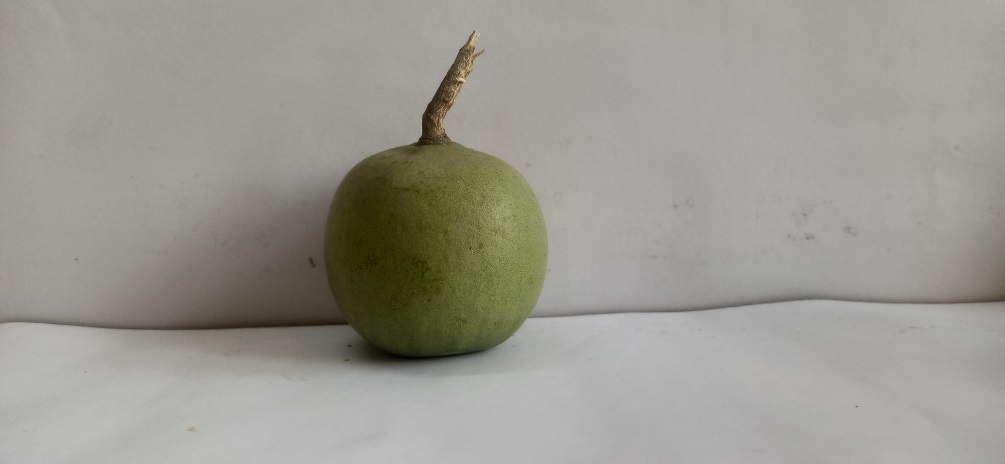 |
| **JMU-Bael (Sel-79)** | **JMU-Bael (Sel-80)** | **NB-5** |
| 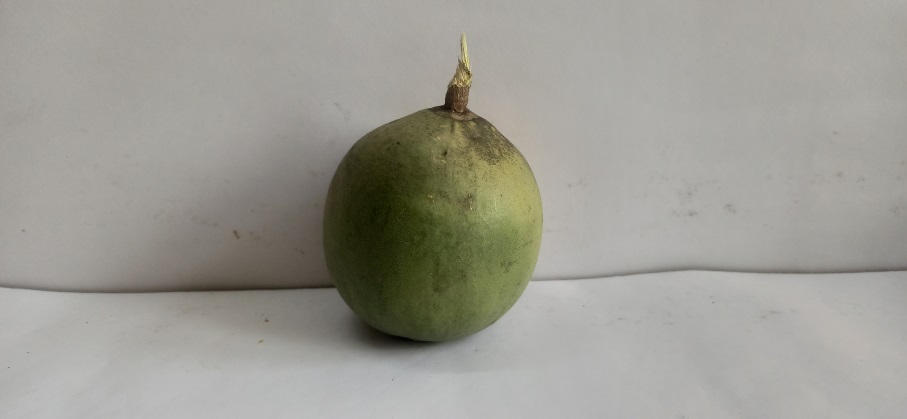 |  |  |
| **NB-9** |  |  |
