# Supplementary material for: Morphology and pomological characterization of bael [Aegle marmelos (L.) Correa] genotypes for climate change mitigation under north-western Himalayas
Source: Front Plant Sci. 2025 Mar 18;16:1496769. doi: 10.3389/fpls.2025.1496769 (PMC11962431; doi:10.3389/fpls.2025.1496769)
Supplement: Supplementary file 2 [file DataSheet2.docx]

**Table S3 Pomological characteristic of bael genotypes.**

| **Genotypes** | **Fruit  length (cm)** | **Fruit  width (cm)** | **Fruit  weight (g)** | **Pulp  weight (g)** | **Pulp  percentage (%)** | **Shell weight/fruit (g)** | **Shell percentage (%)** | **Fruit skull thickness (mm)** |
| --- | --- | --- | --- | --- | --- | --- | --- | --- |
| JMU-Bael (Sel-1) | 10.65±0.1^cde^ | 8.34±0.08^op^ | 412.33±4.16^t^ | 305.31±2.18^t^ | 74.05±0.3^ghijk^ | 95.67±2.4^qr^ | 23.2±0.38^rst^ | 2.57±0.04^vwxy^ |
| JMU-Bael (Sel-2) | 10.93±0.09^bc^ | 8.66±0.11^lmn^ | 477±4.58^op^ | 341.6±5.05^pq^ | 71.61±0.38^lmn^ | 109.51±0.99^l^ | 22.96±0.38^rst^ | 2.91±0.07^cdefgh^ |
| JMU-Bael (Sel-3) | 7.95±0.12^vw^ | 6.84±0.1^wx^ | 221.67±4.04^h1^ | 166.72±4.14^e1^ | 75.21±0.5^efgh^ | 46.69±0.24^c1^ | 21.06±0.38^xy^ | 2.33±0.05^za1b1c1^ |
| JMU-Bael (Sel-4) | 10.43±0.19^ef^ | 10.35±0.22^d^ | 740.67±7.02^g^ | 511.79±2.27^i^ | 69.1±0.5^qrst^ | 202.29±4.6^d^ | 27.31±0.38^c^ | 2.95±0.04^abcd^ |
| JMU-Bael (Sel-5) | 7.35±0.13^b1c1d1^ | 5.13±0.08^f1^ | 376±5.57^vw^ | 260.05±5.34^x^ | 69.16±0.57^qrst^ | 89.25±1.07^stu^ | 23.74±0.38^pqr^ | 2.87±0.06^defghij^ |
| JMU-Bael (Sel-6) | 9.14±0.08^pq^ | 7.95±0.06^r^ | 325.67±3.06^z^ | 219.56±2.57^a1^ | 67.42±0.17^uvwx^ | 88.58±0.62^stu^ | 27.2±0.38^cd^ | 2.93±0.03^abcdef^ |
| JMU-Bael (Sel-7) | 9.47±0.16^klmn^ | 9.25±0.12^hi^ | 475.33±7.51^p^ | 327.29±7.08^r^ | 68.86±0.41^rstu^ | 124.52±0.75^k^ | 26.2±0.38^efg^ | 2.85±0.02^bcdefghijk^ |
| JMU-Bael (Sel-8) | 7.64±0.14^xyza1^ | 8.33±0.11^op^ | 298±8.54^a1^ | 193.83±7.33^b1c1^ | 65.04±0.6^a1b1c1d1^ | 72.1±1.22^w^ | 24.19±0.38^nopq^ | 2.8±0.09^fghijklmno^ |
| JMU-Bael (Sel-9) | 10.54±0.21^de^ | 10.93±0.13^b^ | 851.67±2.52^b^ | 627.42±2.25^c^ | 73.67±0.48^ijk^ | 211.2±4.56^c^ | 24.8±0.46^lmno^ | 2.89±0.09^abcdefghi^ |
| JMU-Bael (Sel-10) | 7±0.17^e1f1^ | 7.6±0.13^s^ | 227.33±7.51^g1h1^ | 154.79±6.42^f1^ | 68.09±0.65^tuvw^ | 51.59±1.09^a1b1^ | 22.69±0.38^stu^ | 2.75±0.06^jklmnopqrs^ |
| JMU-Bael (Sel-11) | 5.03±0.09^o1^ | 5.21±0.11^ef1^ | 75.67±3.06^w1x1^ | 45.5±1.97^q1^ | 60.13±0.17^h1i1^ | 20.58±0.58^i1j1^ | 27.19±0.39^cd^ | 2.84±0.07^defghijkl^ |
| JMU-Bael (Sel-12) | 7.68±0.12^wxyz^ | 6.69±0.18^xy^ | 167.33±4.16^n1^ | 101.66±1.68^i1j1^ | 60.75±0.51^h1^ | 31.9±0.29^e1^ | 19.06±0.38^b1c1d1e1^ | 2.28±0.08^a1b1c1d1^ |
| JMU-Bael (Sel-13) | 9.11±0.13^pq^ | 9.42±0.11^gh^ | 460.67±3.79^r^ | 342.41±3.87^p^ | 74.33±0.26^ghijk^ | 99.86±0.99^o^ | 21.68±0.38^vwx^ | 2.69±0.04^nopqrstuv^ |
| JMU-Bael (Sel-14) | 11.03±0.02^b^ | 8.8±0.18^kl^ | 509±2^n^ | 390.76±3.57^m^ | 76.77±0.4^cd^ | 100.12±1.65^no^ | 19.67±0.38^a1b1^ | 2.23±0.13^c1d1e1^ |
| JMU-Bael (Sel-15) | 7.68±0.18^wxyz^ | 9.49±0.18^gh^ | 484±4.58^o^ | 315.42±2.99^s^ | 65.17±0.41^za1b1c1^ | 131.03±3.01^j^ | 27.07±0.38^cd^ | 2.91±0.04^abcdefg^ |
| JMU-Bael (Sel-16) | 10.38±0.04^ef^ | 9.9±0.06^ef^ | 588.67±2.52^k^ | 404.79±2.7^l^ | 68.76±0.35^rstu^ | 158±2.35^g^ | 26.84±0.38^cdef^ | 2.87±0.02^abcdefghij^ |
| JMU-Bael (Sel-17) | 9.82±0.14^hij^ | 8.18±0.16^pqr^ | 366.33±4.16^x^ | 261.54±4.63^x^ | 71.39±0.48^n^ | 92.56±0.55^rs^ | 25.27±0.38^hijk^ | 2.93±0.02^abcde^ |
| JMU-Bael (Sel-18) | 10.75±0.1^bcd^ | 8.09±0.25^qr^ | 510.33±3.06^mn^ | 363.66±0.34^o^ | 71.26±0.38^n^ | 139.33±2.77^h^ | 27.3±0.38^c^ | 2.95±0.02^abcd^ |
| JMU-Bael (Sel-19) | 6.11±0.21^h1i1^ | 5.89±0.12^a1^ | 106.67±5.69^r1s1^ | 65.43±1.41^n1^ | 61.34±2.13^g1h1^ | 27.47±1.08^f1^ | 25.76±0.38^ghij^ | 2.88±0.03^abcdefgh^ |
| JMU-Bael (Sel-20) | 9.76±0.24^hij^ | 8.92±0.25^jk^ | 386.67±5.13^u^ | 284.86±2.52^v^ | 73.67±0.33^ijk^ | 85.7±2.6^u^ | 22.16±0.38^uvw^ | 2.65±0.05^rstuvw^ |
| JMU-Bael (Sel-21) | 10.71±0.14^cd^ | 10.94±0.01^b^ | 807±3.61^d^ | 556.65±5.55^f^ | 68.98±0.4^rst^ | 219.25±2.22^b^ | 27.17±0.38^cd^ | 2.87±0.02^abcdefghij^ |
| JMU-Bael (Sel-22) | 7.82±0.19^vwx^ | 7.13±0.19^uv^ | 239.67±4.73^e1^ | 154.67±4.46^f1^ | 64.54±0.6^b1c1d1e1^ | 62.73±0.46^y^ | 26.18±0.38^efg^ | 2.98±0.06^a^ |
| JMU-Bael (Sel-23) | 7.86±0.14^vwx^ | 9.98±0.15^ef^ | 470.33±5.51^pq^ | 327.96±2.58^r^ | 69.73±0.33^opqr^ | 101.98±2.9^1mnop^ | 21.68±0.38^vwx^ | 2.77±0.03^ijklmnopqr^ |
| JMU-Bael (Sel-24) | 8.67±0.13^rs^ | 9.81±0.16^f^ | 507±5.57^n^ | 379.5±6.1^n^ | 74.85±0.42^efghi^ | 109.4±1.15^l^ | 21.58±0.38^wx^ | 2.67±0.04^pqrstuvw^ |
| JMU-Bael (Sel-25) | 9.84±0.2^hi^ | 10.76±0.21^bc^ | 619.33±6.03^j^ | 430.66±6.68^k^ | 69.54±0.46^opqrst^ | 155.19±1.6^g^ | 25.06±0.38^jkl^ | 2.97±0.04^ab^ |
| JMU-Bael (Sel-26) | 8.79±0.18^rs^ | 8.42±0.1^nop^ | 375.33±4.51^w^ | 273.65±3.52^w^ | 72.91±0.4^klm^ | 81.68±1.88^v^ | 21.76±0.38^vwx^ | 2.57±0.03^vwxy^ |
| JMU-Bael (Sel-27) | 12.05±0.07^a^ | 11.72±0.02^a^ | 917.65±0.44^a^ | 746.81±1.55^a^ | 81.38±0.15^a^ | 155.66±0.61^g^ | 16.96±0.07^h1^ | 1.80±0.04^h1^ |
| JMU-Bael (Sel-28) | 5.09±0.04^o1^ | 5.25±0.11^ef1^ | 58.33±3.06^y1^ | 37.61±2.52^r1^ | 64.47±0.99^b1c1d1e1^ | 12.54±0.53^m1n1^ | 21.49±0.38^wx^ | 2.52±0.03^xy^ |
| JMU-Bael (Sel-29) | 8.03±0.1^uv^ | 7.95±0.13^r^ | 288±3.61^b1^ | 226.6±4.13^a1^ | 78.68±0.45^b^ | 51.08±0.49^a1b1^ | 17.74±0.38^g1^ | 1.99±0.04^g1^ |
| JMU-Bael (Sel-30) | 9.45±0.2l^mno^ | 8.33±0.19^op^ | 384.33±4.51^uv^ | 282.11±2.15^v^ | 73.4±0.36^ijk^ | 91.02±2.47^st^ | 23.68±0.38^pqr^ | 2.85±0.05^bcdefghijk^ |
| JMU-Bael (Sel-31) | 9.87±0.03^hi^ | 9.26±0.03^hi^ | 835±3^c^ | 579.75±2.02^e^ | 69.43±0.04^pqrst^ | 225.42±2.86^a^ | 27±0.28^cd^ | 2.64±0.02^rstuvwx^ |
| JMU-Bael (Sel-32) | 10.41±0.22^ef^ | 6.99±0.12^vw^ | 329±6.56^z^ | 234.85±6.46^z^ | 71.38±0.57^n^ | 70.55±0.68^w^ | 21.44±0.38^wx^ | 2.52±0.06^xy^ |
| JMU-Bael (Sel-33) | 9.43±0.43^mno^ | 9.44±0.17^gh^ | 511.67±6.51^mn^ | 365.75±3.14^o^ | 71.48±0.3^mn^ | 99.59±3.16^pq^ | 19.46±0.38^a1b1c1^ | 2.25±0.06^a1b1c1d1e1^ |
| JMU-Bael (Sel-34) | 9.18±0.23^opq^ | 9.31±0.16^ghi^ | 462.33±4.51^qr^ | 338.24±10.22^pq^ | 73.16±2.85^jk^ | 103.89±14.25^mn^ | 22.47±2.86^tu^ | 2.51±0.04^xy^ |
| JMU-Bael (Sel-35) | 9.54±0.17^jklm^ | 8.19±0.18^pqr^ | 378.33±3.51^uvw^ | 278.37±3.69^vw^ | 73.58±0.33^ijk^ | 88.64±0.87^stu^ | 23.43±0.38^qrs^ | 2.88±0.03^abcdefghi^ |
| JMU-Bael (Sel-36) | 10.39±0.06^ef^ | 10.56±0.13^cd^ | 758.67±6.51^f^ | 542.73±2.09^g^ | 71.54±0.38^mn^ | 205.99±4.59^d^ | 27.15±0.38^cd^ | 2.95±0.03^abcd^ |
| JMU-Bael (Sel-37) | 10±0.21^gh^ | 9.16±0.14^ij^ | 517.67±6.03^m^ | 370.01±6.32^o^ | 71.48±0.4^mn^ | 135.61±0.71^hi^ | 26.2±0.38^efg^ | 2.73±0.02^klmnopqrst^ |
| JMU-Bael (Sel-38) | 7.84±0.17^vwx^ | 9.26±0.14^hi^ | 417±8.19^t^ | 295.2±7.59^u^ | 70.79±0.43^nop^ | 104.98±0.87^m^ | 25.18±0.38^ijkl^ | 2.68±0.03^opqrstuvw^ |
| JMU-Bael (Sel-39) | 10.93±0.16^bcd^ | 10.82±0.09^b^ | 815.33±3.06^d^ | 601.93±5.11^d^ | 73.83±0.41^hijk^ | 179.04±2.72^e^ | 21.96±0.38^uvw^ | 2.59±0.05^uvwx^ |
| JMU-Bael (Sel-40) | 10.86±0.17^bcd^ | 9.86±0.19^ef^ | 559±3.61^l^ | 436.55±5.09^k^ | 78.09±0.42^bc^ | 103.91±1.55^mn^ | 18.59±0.38^d1e1f1^ | 2.36±0.03^za1b1^ |
| JMU-Bael (Sel-41) | 9.31±0.16^mnop^ | 10.36±0.12^d^ | 673.33±5.03^h^ | 526.01±1.99^h^ | 78.12±0.32^bc^ | 132.86±3.53^ij^ | 19.73±0.38^a1b1^ | 2.25±0.06^a1b1c1d1e1^ |
| JMU-Bael (Sel-42) | 8.27±0.06^tu^ | 6.69±0.04^xy^ | 201.67±5.13^j1k1^ | 133.17±3.68^g1^ | 66.03±0.4^xyza1^ | 54.57±1.52^za1b1^ | 27.06±0.38^cd^ | 2.81±0.03^efghijklmn^ |
| JMU-Bael (Sel-43) | 7.39±0.23^a1b1c1d1^ | 6.62±0.22^xyz^ | 192.33±5.13^l1^ | 129.1±3.22^g1h1^ | 67.12±0.25^wxyz^ | 49.59±1.96^b1c1^ | 25.79±0.38^ghij^ | 2.93±0.02^abcdef^ |
| JMU-Bael (Sel-44) | 7.72±0.1^wxy^ | 7.23±0.09^tuv^ | 211.67±5.86^i1^ | 129.27±5.15^g1h1^ | 61.07±0.78^h1^ | 51.93±1.04^za1b1^ | 24.54±0.38^lmno^ | 2.81±0.06^efghijklmn^ |
| JMU-Bael (Sel-45) | 9.12±0.17^pq^ | 9.91±0.16^ef^ | 457.33±5.03^r^ | 334.17±5.45^qr^ | 73.07±0.46^jkl^ | 103.72±1.21^mno^ | 22.68±0.38^stu^ | 2.78±0.04^hijklmnopq^ |
| JMU-Bael (Sel-46) | 9.48±0.14^klm^ | 7.09±0.17^uv^ | 256±5.57^d1^ | 180.41±4.89^d1^ | 70.47±0.41^nopq^ | 70.46±0.79^w^ | 27.52±0.38^c^ | 2.91±0.04^abcdefg^ |
| JMU-Bael (Sel-47) | 7.67±0.12^wxyz^ | 7.07±0.15^uvw^ | 204.67±3.51^i1j1^ | 123.57±1.93^h1^ | 60.38±0.27^h1i1^ | 53.35±0.61^za1b1^ | 26.07±0.38^fg^ | 2.92±0.05^abcdef^ |
| JMU-Bael (Sel-48) | 6.72±0.1^g1^ | 5.62±0.1^bcd1^ | 112.67±3.51^q1r1^ | 74.05±3.24^l1m1^ | 65.72±0.83^yza1b1^ | 25.53±0.47^f1g1^ | 22.66±0.38^stu^ | 2.7±0.03^mnopqrstuv^ |
| JMU-Bael (Sel-49) | 8.01±0.1^uv^ | 7.26±0.1^tu^ | 194.33±4.04^k1l1^ | 132.62±3.41^g1^ | 68.24±0.41^stuvw^ | 56.47±0.82^z^ | 29.06±0.38^b^ | 2.88±0.04^abcdefghij^ |
| JMU-Bael (Sel-50) | 7.59±0.3^xyza1b1^ | 6.4±0.32^z^ | 115.67±6.03^q1^ | 70.05±4.98^l1m1n1^ | 60.56±1.15^h1^ | 27.98±1.09^f1^ | 24.19±0.38^nopq^ | 2.83±0.02^defghijklm^ |
| JMU-Bael (Sel-51) | 6.37±0.16^h1^ | 5.72±0.15^a1bc1^ | 108±5.57^q1r1s1^ | 63.79±4.54^n1o1^ | 59.06±1.4^i1j1^ | 32.6±2.08^e1^ | 30.18±0.39^a^ | 2.86±0.03^abcdefghij^ |
| JMU-Bael (Sel-52) | 5.83±0.19^j1k1l1^ | 7.03±0.22^uvw^ | 137.33±5.03^p1^ | 83.47±4.13^k1^ | 60.78±0.81^h1^ | 34.2±0.94^e1^ | 24.9±0.38^klmn^ | 2.99±0.03^a^ |
| JMU-Bael (Sel-53) | 7.52±0.26^yzabc1^ | 6.55±0.24^yz^ | 176.33±6.66^m1^ | 122.94±5.89^h1^ | 69.72±0.77^opqrs^ | 33.81±0.98^e1^ | 19.17±0.38^b1c1d1^ | 2.28±0.04^a1b1c1d1^ |
| JMU-Bael (Sel-54) | 7.78±0.12^vwxy^ | 7.45±0.08^st^ | 237.67±2.52^e1f1^ | 150.59±2.61^f1^ | 63.36±0.48^e1f1^ | 64.55±0.62^xy^ | 27.16±0.38^cd^ | 2.65±0.05^rstuvw^ |
| JMU-Bael (Sel-55) | 9.19±0.15^nop^ | 9.48±0.17^gh^ | 443±3^s^ | 344.21±4.02^p^ | 77.7±0.42^bc^ | 79.73±1.3^v^ | 18±0.38^f1g1^ | 2.16±0.04^d1e1f1^ |
| JMU-Bael (Sel-56) | 8.61±0.24^s^ | 10.5±0.05^d^ | 660±7.55^i^ | 452.89±7.82^j^ | 68.62±0.43^rstuv^ | 171.78±1.28^f^ | 26.03±0.38^gh^ | 2.8±0.02^fghijklmnopq^ |
| JMU-Bael (Sel-57) | 8.9±0.16^qr^ | 8.69±0.17^klm^ | 371.33±5.69^wx^ | 257.6±5.12^x^ | 69.37±0.33^pqrst^ | 87.8±2.75^stu^ | 23.64±0.38^pqr^ | 2.79±0.05^ghijklmnopqr^ |
| JMU-Bael (Sel-58) | 9.27±0.13^mno^ | 9.56±0.14^g^ | 478.67±3.21^op^ | 377.5±4.14^n^ | 78.87±0.39^b^ | 95.92±1.46^qr^ | 20.04±0.38^za1^ | 2.38±0.04^za1^ |
| JMU-Bael (Sel-59) | 7.29±0.29^c1d1^ | 8.43±0.21^nop^ | 266.67±4.73^c1^ | 190.31±4.47^c1^ | 71.37±0.42^n^ | 65.7±0.37^xy^ | 24.64±0.38^lmno^ | 2.63±0.05^stuvwx^ |
| JMU-Bael (Sel-60) | 6.89±0.35^f1g1^ | 5.93±0.2^a1^ | 131.67±4.04^p1^ | 99±3.73^j1^ | 75.19±0.52^efgh^ | 24.72±0.3^f1g1h1^ | 18.77±0.38^c1d1e1f1^ | 2.36±0.03^za1b1^ |
| JMU-Bael (Sel-61) | 7.41±0.09^za1b1c1d1^ | 6.48±0.06^yz^ | 166±5.57^n1^ | 101.96±3.86^i1j1^ | 61.42±0.38^g1h1^ | 39.92±1.84^d1^ | 24.05±0.38^opq^ | 2.87±0.03^abcdefghij^ |
| JMU-Bael (Sel-62) | 5.94±0.08^i1j1k1^ | 5.38±0.12^def1^ | 95.67±2.08^t1u1^ | 56.1±1.95^p1^ | 58.64±0.76^j1^ | 23.28±0.15^g1h1i1^ | 24.33±0.38^mno^ | 2.88±0.04^abcdefghi^ |
| JMU-Bael (Sel-63) | 5.92±0.2^i1j1k1^ | 5.44±0.11^de1^ | 93.67±4.73^u1^ | 56.53±1.51^o1p1^ | 60.35±1.45^h1i1^ | 24.38±0.88^g1h1i1^ | 26.03±0.39^gh^ | 2.84±0.02^cdefghijkl^ |
| JMU-Bael (Sel-64) | 7±0.19^e1f1^ | 6.39±0.16^z^ | 148.67±9.29^o1^ | 99.67±7.54^j1^ | 67.04±0.87^wxyz^ | 34.23±1.61^e1^ | 23.02±0.39^rst^ | 2.33±0.05^za1b1c1^ |
| JMU-Bael (Sel-65) | 6.02±0.12^i1j1^ | 5.87±0.08^a1b1^ | 108.67±7.02^q1r1s1^ | 68.19±5.76^m1n1^ | 62.75±1.25^fg1^ | 24.93±1.27^f1g1^ | 22.94±0.38^rst^ | 2.67±0.05^qrstuv^ |
| JMU-Bael (Sel-66) | 4.6±0.08^p1^ | 4.64±0.11^g1^ | 56.33±1.53^y1^ | 34.63±2.19^s1^ | 61.47±4.8^g1h1^ | 10.26±0.4^n1^ | 18.21±0.38^f1g1^ | 2.25±0.04^bc1d1e1^ |
| JMU-Bael (Sel-67) | 10.17±0.1^fg^ | 10.09±0.14^e^ | 768.33±4.04^e^ | 547.19±5.52^g^ | 71.22±0.4^n^ | 203.22±2.31^d^ | 26.45±0.38^defg^ | 2.97±0.02^abc^ |
| JMU-Bael (Sel-68) | 7.65±0.08^xyza1^ | 8.53±0.06^mno^ | 341.33±3.21^y^ | 259.19±2.32^x^ | 75.94±0.32^de^ | 62.47±1.7^y^ | 18.3±0.38^e1f1g1^ | 2.31±0.03^a1b1c1^ |
| JMU-Bael (Sel-69) | 6.92±0.1^e1f1g1^ | 7.56±0.08^s^ | 230.67±2.52^f1g1^ | 157.61±1.32^f1^ | 68.33±0.31^rstuvw^ | 41.89±1.3^d1^ | 18.16±0.38^f1g1^ | 2.2±0.04^c1d1e1f1^ |
| JMU-Bael (Sel-70) | 6.9±0.23^f1g1^ | 8.85±0.18^kl^ | 281.67±7.02^b1^ | 199.74±6.61^b1^ | 70.91±0.58^no^ | 62.34±0.64^y^ | 22.13±0.38^uvw^ | 2.81±0.61^efghijklmn^ |
| JMU-Bael (Sel-71) | 8.32±0.22^t^ | 9.49±0.19^gh^ | 438.33±8.74^s^ | 302.32±7.83^tu^ | 68.97±0.42^rst^ | 112.5±0.94^l^ | 25.66±0.38^ghij^ | 2.84±0.04^defghijkl^ |
| JMU-Bael (Sel-72) | 5.11±0.2^o1^ | 5.21±0.19^ef1^ | 70.67±4.73^x1^ | 44.98±3.99^q1r1^ | 63.65±1.36^d1e1f1^ | 16.21±0.83^k1l1m1^ | 22.94±0.38^rst^ | 2.72±0.04^lmnopqrstuv^ |
| JMU-Bael (Sel-73) | 9.66±0.17^ijkl^ | 8.03±0.21^r^ | 324.67±4.16^z^ | 246.1±2.16^y^ | 75.8±0.31^def^ | 68.22±2.11^wx^ | 21.01±0.38^xy^ | 2.44±0.06^yz^ |
| JMU-Bael (Sel-74) | 9.73±0.12^hijk^ | 10.95±0.1^b^ | 848.33±4.04^b^ | 639.12±1.62^b^ | 75.34±0.36^defg^ | 174.6±4^f^ | 20.58±0.38^yz^ | 2.7±0.04^nopqrstuv^ |
| JMU-Bael (Sel-75) | 5.56±0.24^lm^ | 5.79±0.13^a1bc1^ | 83.67±4.73^v1w1^ | 55.75±4.02^p1^ | 66.63±1.03^xyz^ | 18.14±0.72^j1k1l1^ | 21.69±0.38^vwx^ | 2.56±0.05^wxyz^ |
| JMU-Bael (Sel-76) | 5.53±0.18^mn^ | 5.56±0.21^cd1^ | 78±4^v1w1x1^ | 49.94±3.46^p1q1^ | 64.03±1.16^c1d1e1f1^ | 14.47±0.53^l1m1^ | 18.55±0.38^d1e1f1^ | 2.23±0.04^c1d1e1^ |
| JMU-Bael (Sel-77) | 5.27±0.29^no^ | 5.4±0.27^de1^ | 84.33±8.02^v1^ | 55.55±6.38^p1^ | 65.87±1.32^yza1b1^ | 18.59±1.53^j1k1^ | 22.04±0.38^uvw^ | 2.52±0.06^xy^ |
| JMU-Bael (Sel-78) | 5.69±0.15^klm^ | 5.8±0.15^a1bc1^ | 103±7.94^s1t1^ | 76.72±6.85^k1l1^ | 74.48±0.9^fghij^ | 18.73±1.06^j1k1^ | 18.18±0.38^f1g1^ | 2.15±0.05^e1f1^ |
| JMU-Bael (Sel-79) | 7.2±0.34^de^ | 7±0.3^vw^ | 180±11.14^m1^ | 108.26±7.38^i1^ | 60.14±0.51^h1i1^ | 46.7±2.28^c1^ | 25.95±0.38^ghi^ | 2.96±0.02^abcd^ |
| JMU-Bael (Sel-80) | 6.01±0.15^i1j1^ | 5.62±0.15^cd1^ | 105.33±8.5^r1s1^ | 63.97±7.14^n1^ | 60.73±1.87^h1^ | 20.81±1.36^h1i1j1^ | 19.75±0.38^a1b1^ | 2.44±0.05^yz^ |
| NB-5 | 9.29±0.01^mnop^ | 8.93±0.06^jk^ | 674.33±6.03^h^ | 452.81±3.83^j^ | 67.15±0.1^vwxyz^ | 181.73±2.21^e^ | 26.95±0.1^cde^ | 2.09±0.03^f1g1^ |
| NB-9 | 9.89±0^ghi^ | 9.3±0.03^hi^ | 830±6^c^ | 574.92±2.72^e^ | 69.27±0.27^qrst^ | 225.35±3.62^a^ | 27.15±0.25^cd^ | 2.62±0.02^tuvwx^ |
| **C.D. at 5%** | 0.26 | 0.23 | 7.68 | 6.02 | 1.35 | 3.61 | 0.50 | 0.13 |

Value represent the mean±SD (Standard deviation)
Mean with different letters in each column is significantly different from each other at *P*< 0.05 (Fisher’s least significant difference)

Continue Table 1

Continue Table 1

| **Genotypes** | **Inner diameter (cm)** | **Seed length (mm)** | **Seed diameter (mm)** | **Number of seed sack per fruit** | **Number of seeds per sack** | **Number of seeds per fruit** | **Total seed weight per fruit (g)** | **Test seed weight per fruit (g/100 seeds)** |
| --- | --- | --- | --- | --- | --- | --- | --- | --- |
| JMU-Bael (Sel-1) | 8.09±0.08^tuv^ | 10.08±0.06^b^ | 5.94±0.04^klmn^ | 11.33±0.58^kl^ | 7.33±0.58^opqr^ | 75.67±4.16^e1f1^ | 11.35±0.33^h1i1j1^ | 15.02±0.44^i1j1^ |
| JMU-Bael (Sel-2) | 8.37±0.12^qrs^ | 6.84±0.04^stuvw^ | 5.74±0.03^opqrst^ | 11.67±0.58^jk^ | 12.67±0.58^de^ | 133.33±3.06^nop^ | 25.89±0.25^jk^ | 19.42±0.27^opqrstuvwxy^ |
| JMU-Bael (Sel-3) | 6.61±0.1^b1c1d1^ | 6.57±0.04^xyza1b1^ | 6.38±0.04^ef^ | 10.33±0.58^mn^ | 4.67±0.58^tu^ | 45±2.65^o1^ | 8.25±0.12^m1n1o1^ | 18.37±0.88^vwxyza1b1c1d1^ |
| JMU-Bael (Sel-4) | 10.05±0.22^d^ | 5.09±0.04^n1o1p1^ | 5.03±0.04^xy^ | 12.67±0.58^hi^ | 11.33±0.58^fgh^ | 138.33±3.06^jklm^ | 26.58±1.12^ij^ | 19.21±0.39^pqrstuvwxyz^ |
| JMU-Bael (Sel-5) | 4.84±0.08^n1^ | 8.64±0.03^e^ | 5.96±0.02^jklmn^ | 15.67±0.58^bc^ | 9.33±0.58^klm^ | 139.33±3.06^ijkl^ | 26.7±0.81^ij^ | 19.16±0.32^pqrstuvwxyza1^ |
| JMU-Bael (Sel-6) | 7.66±0.06^w^ | 9.91±0.04^b^ | 6.77±0.04^b^ | 10.67±0.58^lm^ | 6.67±0.58^qr^ | 71±2.65^f1g1^ | 17.53±1.12^vwx^ | 24.7±1.58^abc^ |
| JMU-Bael (Sel-7) | 8.96±0.12^k^ | 8.22±0.04^ghi^ | 5.82±0.04^mnop^ | 16.33±0.58^ab^ | 8.33±0.58^mno^ | 130±2^opq^ | 23.52±0.38^mn^ | 18.09±0.03^xyza1b1c1d1e1^ |
| JMU-Bael (Sel-8) | 8.05±0.1^uv^ | 7.73±0.05^klm^ | 5.39±0.03^vw^ | 10.67±0.58^lm^ | 14.33±0.58^bc^ | 146.67±3.06^gh^ | 32.07±0.74^f^ | 21.87±0.21^efghijkl^ |
| JMU-Bael (Sel-9) | 10.64±0.13^b^ | 7.81±0.04^kl^ | 6.58±0.03^cd^ | 12.33±0.58^ij^ | 7.33±0.58^opqr^ | 81.33±3.51^c1d1^ | 13.05±0.2^e1f1^ | 16.05±0.44^f1g1h1i1j1^ |
| JMU-Bael (Sel-10) | 7.32±0.12^x^ | 7.35±0.03^no^ | 6.05±0.03^ghijkl^ | 8.67±0.58^pq^ | 14.67±0.58^b^ | 127±5.57^q^ | 20.95±0.39^op^ | 16.51±0.43^e1f1g1h1i1^ |
| JMU-Bael (Sel-11) | 4.93±0.1^m1n1^ | 6.77±0.02^uvwxyz^ | 4.81±0.02^za1b1^ | 12.33±0.58^ij^ | 4.67±0.58^tu^ | 48±2^n1o1^ | 9.59±0.54^l1m1^ | 19.97±0.48^mnopqrstuvw^ |
| JMU-Bael (Sel-12) | 6.46±0.18^c1d1e1^ | 7.71±0.03^lm^ | 6.77±0.03^b^ | 9.67±0.58^no^ | 15±1^b^ | 147.33±4.16^fg^ | 33.77±2.34^e^ | 22.95±2.07^cdefghijk^ |
| JMU-Bael (Sel-13) | 9.15±0.11^hijk^ | 7.95±0.02^jk^ | 5.64±0.04^pqrst^ | 10.33±0.58^mn^ | 9.67±0.58^jkl^ | 96.33±4.51^x^ | 18.39±0.89^stuv^ | 19.09±0.08^pqrstuvwxyza1b1^ |
| JMU-Bael (Sel-14) | 8.58±0.17^opq^ | 6.43±1.16^a1b1c1d1^ | 5.7±0.01^pqrst^ | 11.33±0.58^kl^ | 10±1^ijk^ | 101.67±3.06^vw^ | 18.12±0.75^tuvw^ | 17.82±0.25^xyza1b1c1d1e1f1^ |
| JMU-Bael (Sel-15) | 9.2±0.18^hijk^ | 8.13±0.05^hij^ | 6.38±0.03^ef^ | 11.67±0.58^jk^ | 14.33±0.58^bc^ | 164.67±3.06^c^ | 37.55±1.27^c^ | 22.8±0.68^defg^ |
| JMU-Bael (Sel-16) | 9.61±0.06^ef^ | 8.35±0.04^fgh^ | 5.9±0.02^ghij^ | 10.67±0.58^lm^ | 12±1^efg^ | 134.33±4.04^mno^ | 25.87±0.23^jk^ | 19.27±0.43^pqrstuvwxy^ |
| JMU-Bael (Sel-17) | 7.89±0.15^vw^ | 6.33±0.04^c1d1e1^ | 6.76±0.04^bc^ | 12.67±0.58^hi^ | 5.33±0.58^st^ | 62.67±2.08^i1j1^ | 12.24±0.29^f1g1h1^ | 19.53±0.18^nopqrstuvwx^ |
| JMU-Bael (Sel-18) | 7.79±0.25^w^ | 6.52±0.03^za1b1c1^ | 5.37±0.04^w^ | 9.33±0.58^op^ | 4.67±0.58^tu^ | 43.33±1.53^o1p1^ | 7.34±0.22^o1^ | 16.95±0.15^c1d1e1f1g1h1^ |
| JMU-Bael (Sel-19) | 5.6±0.12^h1i1^ | 5.85±0.03^hij1j1^ | 5.41±0.03^uvw^ | 12.33±0.58^ij^ | 8.33±0.58^mno^ | 95.33±2.31^x^ | 13.76±3.46^d1e1^ | 14.47±3.83^j1^ |
| JMU-Bael (Sel-20) | 8.66±0.25^nop^ | 9.56±0.05^c^ | 7.12±0.04^a^ | 11.33±0.58^kl^ | 8±0^nop^ | 85.33±2.52^a1b1c1^ | 16.11±0.13^yza1^ | 18.89±0.4^qrstuvwxyza1b1^ |
| JMU-Bael (Sel-21) | 10.66±0.02^b^ | 7.24±0.03^opq^ | 6.02±0.04^hijklm^ | 12.33±0.58^ij^ | 12.33±0.58^def^ | 143.67±1.53^ghi^ | 31.1±0.22^fg^ | 21.65±0.08^fghijklm^ |
| JMU-Bael (Sel-22) | 6.83±0.19^za1b1^ | 7.25±0.04^opq^ | 5.91±0.02^lmno^ | 12.67±0.58^hi^ | 10±1^ijk^ | 128.67±3.06^pq^ | 22.26±0.1^no^ | 17.31±0.34^b1c1d1e1f1g1h1^ |
| JMU-Bael (Sel-23) | 9.7±0.15^ef^ | 6.34±0.04^b1c1d1e1^ | 5.96±0.04^jklmn^ | 16.67±0.58^a^ | 11.33±0.58^fgh^ | 171±2^b^ | 40.4±0.25^b^ | 23.62±0.14^bcdef^ |
| JMU-Bael (Sel-24) | 9.54±0.16^fg^ | 6.76±0.02^vwxyz^ | 5.62±0.02^qrst^ | 15.33±0.58^cd^ | 7±1^pqr^ | 94.67±3.06^xy^ | 18.1±0.02^uvw^ | 19.13±0.59^pqrstuvwxyza1^ |
| JMU-Bael (Sel-25) | 10.46±0.21^bc^ | 6.53±0.03^za1b1c1^ | 4.57±0.55^d1e1^ | 11.67±0.58^jk^ | 13±1^de^ | 152±4^ef^ | 33.48±0.21^e^ | 22.04±0.45^defghijk^ |
| JMU-Bael (Sel-26) | 8.16±0.1^stu^ | 8.09±0.03^ij^ | 6.11±0.03^ghijk^ | 12.67±0.58^hi^ | 9.33±0.58^klm^ | 114.67±2.08^t^ | 20±0.13^pqr^ | 17.45±0.2^za1b1c1d1e1f1g1^ |
| JMU-Bael (Sel-27) | 11.54±0.02^a^ | 8.57±0.02^ef^ | 6.49±0.02^de^ | 14.33±0.58^ef^ | 6.67±0.58^qr^ | 85.33±2.52^a1b1c1^ | 15.18±0.9^za1b1c1^ | 16.69±0.28^d1e1f1g1h1i1^ |
| JMU-Bael (Sel-28) | 5±0.11^n1^ | 4.64±0.03^q1^ | 4.14±0.03^h1i1^ | 12.33±0.58^ij^ | 4.33±0.58^tuv^ | 45±2.65^o1^ | 8.19±0.06^n1o1^ | 18.24±0.92^wxyza1b1c1d1e1^ |
| JMU-Bael (Sel-29) | 7.75±0.13^w^ | 6.22±0.03^d1e1f1^ | 5.76±0.03^opq^ | 11.67±0.58^jk^ | 5.33±0.58^st^ | 58.33±1.53^j1k1^ | 10.31±0.06^i1j1k1l1^ | 17.69±0.37^yza1b1c1d1e1f1^ |
| JMU-Bael (Sel-30) | 8.05±0.19^uv^ | 7.06±0.03^pqrst^ | 5.55±0.04^stuv^ | 14.33±0.58^ef^ | 4.67±1.15^tu^ | 57.67±2.52^k1l1^ | 11.2±0.12^h1i1j1k1^ | 19.45±0.65^opqrstuvwxy^ |
| JMU-Bael (Sel-31) | 9±0.03^jkl^ | 7.03±0.05^qrst^ | 5.91±0.03^lmno^ | 10.67±0.58^lm^ | 12.33±0.58^def^ | 128.33±2.52^q^ | 29.83±1.94^gh^ | 23.24±1.43^cdefg^ |
| JMU-Bael (Sel-32) | 6.74±0.12^za1b1^ | 6.87±0.04^stuv^ | 5.61±0.03^qrst^ | 14.33±0.58^ef^ | 7.33±0.58^opqr^ | 128.67±3.51^pq^ | 23.59±0.25^mn^ | 18.34±0.32^vwxyza1b1c1d1e1^ |
| JMU-Bael (Sel-33) | 9.21±0.17^hij^ | 7.18±0.04^opqr^ | 5.14±0.04^x^ | 11.67±0.58^jk^ | 17.33±0.58^a^ | 182.33±3.21^a^ | 46.33±0.5^a^ | 25.41±0.19^ab^ |
| JMU-Bael (Sel-34) | 9.06±0.16^ijkl^ | 8.28±0.03^ghi^ | 6.13±0.04^ghi^ | 9.33±0.58^op^ | 12.33±0.58^def^ | 106.67±3.06^u^ | 20.21±0.17^pqr^ | 18.95±0.39^qrstuvwxyza1b1^ |
| JMU-Bael (Sel-35) | 7.9±0.18^vw^ | 7.12±0.03^opqr^ | 6.46±0.04^de^ | 11.33±0.58^kl^ | 6.33±0.58^rs^ | 65.33±3.06^h1i1^ | 11.33±0.31^h1i1j1^ | 17.35±0.44^a1b1c1d1e1f1g1h1^ |
| JMU-Bael (Sel-36) | 10.26±0.13^cd^ | 8.22±0.02^ghi^ | 6.48±0.02^de^ | 11.67±0.58^jk^ | 5.33±0.58^st^ | 55.67±3.21^k1l1^ | 9.94±0.26^k1l1^ | 17.88±0.64^xyza1b1c1d1e1^ |
| JMU-Bael (Sel-37) | 8.89±0.13^lmn^ | 9.05±0.03^d^ | 6.21±0.03^fg^ | 10.33±0.58^mn^ | 7±0^pqr^ | 69.33±1.15^g1h1^ | 12.04±0.08^f1g1h1^ | 17.37±0.21^a1b1c1d1e1f1g1h1^ |
| JMU-Bael (Sel-38) | 9±0.14^jkl^ | 7±0.02^rstu^ | 4.24±0.02^g1h1^ | 12.33±0.58^ij^ | 7.33±0.58^opqr^ | 83±2.65^b1c1d1^ | 16.81±0.42^wxyz^ | 20.26±0.15^klmnopqrstu^ |
| JMU-Bael (Sel-39) | 10.56±0.09^b^ | 7.74±0.02^klm^ | 6±0.02^i^ | 11.67±0.58^jk^ | 13.33±0.58^cd^ | 155.67±2.08^de^ | 34.36±0.28^de^ | 22.07±0.11^defghij^ |
| JMU-Bael (Sel-40) | 9.62±0.18^ef^ | 7.3±0.62^nop^ | 5.78±0.04^nop^ | 12.33±0.58^ij^ | 8.33±0.58^mno^ | 98.33±2.52^wx^ | 18.54±0.1^stuv^ | 18.86±0.39^qrstuvwxyza1b1^ |
| JMU-Bael (Sel-41) | 10.13±0.11^d^ | 8.27±0.03^ghi^ | 5.58±0.03^rstu^ | 13.67±0.58^fg^ | 5.33±0.58^st^ | 71±3.61^f1g1^ | 14.46±0.34^b1c1d1^ | 20.39±0.58^jklmnopqrstu^ |
| JMU-Bael (Sel-42) | 6.41±0.04^d1e1f1^ | 6±0.02^f1g1h1^ | 3.96±0.03^j1k1l1^ | 8.33±0.58^qr^ | 8.67±0.58^lmn^ | 68±2.65^g1h1^ | 13.93±0.23^c1d1e1^ | 20.49±0.52^ijklmnopqr^ |
| JMU-Bael (Sel-43) | 6.33±0.21^e1f1g1^ | 6.02±0.07^f1g1h1^ | 3.82±0.03^f1g1h1^ | 7.67±0.58^rs^ | 8±0^nop^ | 65.33±0.58^h1i1^ | 13.64±0.04^de^ | 20.87±0.14^hijklmnop^ |
| JMU-Bael (Sel-44) | 6.95±0.09^yza1^ | 5.83±0.03^h1i1j1k1^ | 4.19±0.03^g1h1i1^ | 11.33±0.58^kl^ | 13.33±0.58^cd^ | 143±2^ghij^ | 30.47±0.12^gh^ | 21.31±0.21^ghijklmn^ |
| JMU-Bael (Sel-45) | 9.63±0.16^ef^ | 6.75±0.04^vwxyz^ | 3.58±0.05^no^ | 15.33±0.58^cd^ | 7.33±0.58^opqr^ | 104.33±2.52^uv^ | 19.45±0.21^qrst^ | 18.65±0.26^stuvwxyza1b1c1^ |
| JMU-Bael (Sel-46) | 6.8±0.17^za1b1^ | 4.53±0.02^q1^ | 3.87±0.02^l1^ | 7.33±0.58^s^ | 3.33±0.58^vw^ | 23.33±4.04^r1^ | 5.13±0.1^p1^ | 22.36±3.27^defgh^ |
| JMU-Bael (Sel-47) | 6.77±0.15^za1b1^ | 7.3±0.04^no^ | 5.39±0.04^vw^ | 10.33±0.58^mn^ | 14.33±0.58^bc^ | 142.33±2.52^hijk^ | 27.74±1.33^i^ | 19.49±0.6^opqrstuvwxyz^ |
| JMU-Bael (Sel-48) | 5.35±0.1^j1k1l1^ | 5.62±0.02^j1k1l1^ | 4.8±0.03^a1b1c1^ | 9.67±0.58^no^ | 6.67±0.58^qr^ | 70.33±0.58^g1^ | 13.09±0.14^e1f1^ | 18.61±0.06^stuvwxyza1b1c1^ |
| JMU-Bael (Sel-49) | 6.98±0.11^yz^ | 4.57±0.03^q1^ | 3.9±0.02^k1l1m1^ | 8.33±0.58^qr^ | 3.33±0.58^vw^ | 24.67±1.15^r1^ | 5.25±0.04^p1^ | 21.3±1.01^ghijklmn^ |
| JMU-Bael (Sel-50) | 6.12±0.32^g1^ | 5.93±0.03^g1h1^ | 4.72±0.03^b1c1d1^ | 10.33±0.58^mn^ | 8.67±0.58^lmn^ | 90.33±2.08^yz^ | 17.64±0.3^vwx^ | 19.53±0.12^nopqrstuvwx^ |
| JMU-Bael (Sel-51) | 5.43±0.16^i1j1k1^ | 6.35±0.04^b1c1d1e1^ | 3.1±0.02^q1^ | 8.33±0.58^qr^ | 10.33±0.58^hijk^ | 80±2^d1e1^ | 11.62±1.37^g1h1i1^ | 14.51±1.61^j1^ |
| JMU-Bael (Sel-52) | 6.74±0.21^za1b1^ | 5.6±0.02^kl^ | 4.99±0.04^xyz^ | 10.67±0.58^lm^ | 8.33±0.58^mno^ | 88.33±2.52^za1^ | 19.67±0.09^pqrs^ | 22.27±0.54^defghi^ |
| JMU-Bael (Sel-53) | 6.32±0.23^e1f1g1^ | 7.84±0.04^kl^ | 3.73±0.03^m1n1^ | 12.33±0.58^ij^ | 8±0^nop^ | 96.33±1.53^x^ | 19.58±0.09^qrs^ | 20.33±0.23^jklmnopqrs^ |
| JMU-Bael (Sel-54) | 7.18±0.08^xy^ | 6.81±0.04^tuvwx^ | 4.05±0.04^i1j1k1^ | 11.67±0.58^jk^ | 9.33±0.58^klm^ | 118.33±2.52^st^ | 22.53±0.05^n^ | 19.04±0.37^qrstuvwxyza1b1^ |
| JMU-Bael (Sel-55) | 9.27±0.16^hi^ | 6.55±0.04^yza1b1c1^ | 6.15±0.04^ghi^ | 15.33±0.58^cd^ | 6.67±0.58^qr^ | 103.67±2.52^uv^ | 19.06±0.08^rstu^ | 18.39±0.37^vwxyza1b1c1d1^ |
| JMU-Bael (Sel-56) | 10.22±0.05^cd^ | 6.81±0.03^tuvwx^ | 5.98±0.03^i^ | 13.33±0.58^gh^ | 12.33±0.58^def^ | 155.67±3.06^de^ | 35.33±0.19^d^ | 22.7±0.33^defghijk^ |
| JMU-Bael (Sel-57) | 8.42±0.16^pqr^ | 7.73±0.03^klm^ | 5.89±0.04^lm^ | 16.33±0.58^ab^ | 7.67±0.58^nopq^ | 125.67±2.52^qr^ | 25.94±2.25^jk^ | 20.67±2.19^hijklmnopq^ |
| JMU-Bael (Sel-58) | 9.32±0.14^gh^ | 10.68±0.03^a^ | 5.95±0.03^klmn^ | 14.67±0.58^de^ | 2.33±0.58^w^ | 27.67±3.06^r1^ | 5.24±0.12^p1^ | 19.06±1.62^pqrstuvwxyza1b1^ |
| JMU-Bael (Sel-59) | 8.16±0.2^stu^ | 7.51±0.03^mn^ | 4.13±0.02^h1i1j1^ | 14.33±0.58^ef^ | 5±1^t^ | 57.67±3.51^k1l1^ | 10.66±0.09^i1j1k1l1^ | 18.53±0.99^tuvwxyza1b1c1^ |
| JMU-Bael (Sel-60) | 5.7±0.2^h1^ | 6.61±0.04^wxyza1^ | 5.14±0.55^x^ | 9.33±0.58^op^ | 4.33±0.58^tuv^ | 39.67±3.06^p1q1^ | 7.95±0.08^o1^ | 20.12±1.33^lmnopqrstuv^ |
| JMU-Bael (Sel-61) | 6.19±0.06^f1g1^ | 5.87±0.02^h1i1^ | 4.75±0.04^a1b1c1^ | 12.33±0.58^ij^ | 11±1^ghi^ | 121±3^rs^ | 24.12±0.07^lm^ | 19.94±0.44^mnopqrstuvw^ |
| JMU-Bael (Sel-62) | 5.09±0.12^m1n1^ | 6.27±0.03^d1e1^ | 4.42±0.03^e1f1^ | 12.33±0.58^ij^ | 6.33±0.58^rs^ | 75.33±3.06^e1f1^ | 16.29±0.05^xyz^ | 21.65±0.82^fghijklm^ |
| JMU-Bael (Sel-63) | 5.15±0.11^l1m1^ | 5.54±0.04^l1^ | 4.33±0.04^f1g1^ | 11.67±0.58^jk^ | 7.33±0.58^opqr^ | 80.67±2.08^c1d1^ | 12.76±2.36^e1f1g1^ | 15.85±3.19^g1h1i1j1^ |
| JMU-Bael (Sel-64) | 6.16±0.16^g1^ | 5.82±0.04^h1i1j1k1^ | 5.35±0.03^h1i1j1k1^ | 9.33±0.58^op^ | 7.33±0.58^opqr^ | 57.67±3.51^k1l1^ | 14.77±0.36^a1b1c1d1^ | 25.65±0.96^a^ |
| JMU-Bael (Sel-65) | 5.6±0.08^h1i1^ | 5.87±0.02^h1i1^ | 5.37±0.02^vw^ | 11.33±0.58^kl^ | 8±1^nop^ | 71.33±3.51^f1g1^ | 15.54±0.13^yza1b1^ | 21.81±0.89^fghijklm^ |
| JMU-Bael (Sel-66) | 4.41±0.1^o1^ | 4.65±0.03^q1^ | 3.09±0.03^q1^ | 10.33±0.58^mn^ | 5.33±0.58^st^ | 53.33±3.06^l1m1^ | 11.45±2.98^g1h1i1j1^ | 21.3±4.52^ghijklmn^ |
| JMU-Bael (Sel-67) | 9.79±0.15^e^ | 5.45±0.04^l1m1^ | 4.75±0.04^l1m1^ | 13.33±0.58^gh^ | 6.33±0.58^rs^ | 82.67±2.08^b1c1d1^ | 17.93±0.05^uvw^ | 21.69±0.48^fghijklm^ |
| JMU-Bael (Sel-68) | 8.3±0.07^rst^ | 5.18±0.03^no^ | 5.79±0.02^n1o1^ | 13.67±0.58^fg^ | 7.33±0.58^opqr^ | 101.33±3.51^vw^ | 19.67±0.18^pqrs^ | 19.42±0.5^opqrstuvwxy^ |
| JMU-Bael (Sel-69) | 7.34±0.07^x^ | 4.95±0.04^o1p1^ | 4.28±0.04^f1g1h1^ | 12.67±0.58^hi^ | 12.33±0.58^def^ | 147.33±3.51^fg^ | 31.16±0.1^fg^ | 21.16±0.44^ghijklmno^ |
| JMU-Bael (Sel-70) | 8.57±0.13^opq^ | 5.66±0.04^i1j1k1l1^ | 5.01±0.55^xy^ | 15.33±0.58^cd^ | 7.33±0.58^opqr^ | 106±2.65^uv^ | 19.58±0.11^qrs^ | 18.48±0.36^uvwxyza1b1c1d1^ |
| JMU-Bael (Sel-71) | 9.2±0.2^hijk^ | 5.96±0.03^g1h1^ | 6.18±0.03^gh^ | 11.67±0.58^jk^ | 10.33±0.58^hijk^ | 116.33±2.08^st^ | 23.52±0.36^mn^ | 20.22±0.09^lmnopqrstu^ |
| JMU-Bael (Sel-72) | 4.94±0.19^m1n1^ | 5.13±0.02^no1p1^ | 3.29±0.02^p1^ | 8.33±0.58^qr^ | 6.33±0.58^rs^ | 50.67±3.06^m1n1^ | 9.47±0.11^l1m1n1^ | 18.73±0.9^qrstuvwxyza1b1c1^ |
| JMU-Bael (Sel-73) | 7.78±0.21^w^ | 7.69±0.02^lm^ | 5.49±0.02^tu^ | 11.67±0.58^jk^ | 4.67±0.58^tu^ | 55.67±4.93^k1l1^ | 10.34±0.15^i1j1k1l1^ | 18.66±1.47^stuvwxyza1b1c1^ |
| JMU-Bael (Sel-74) | 10.68±0.1^b^ | 8.23±0.03^ghi^ | 6.62±0.03^bcd^ | 16.33±0.58^ab^ | 10.67±0.58^hij^ | 159.67±4.16^d^ | 34.62±0.12^de^ | 21.69±0.49^fghijklm^ |
| JMU-Bael (Sel-75) | 5.53±0.13^h1i1j1k1^ | 5.27±0.02^mn^ | 3.5±0.02^o1^ | 11.33±0.58^kl^ | 5.33±0.58^st^ | 53.67±3.06^k1l1m1^ | 9.78±0.08^l1^ | 18.25±0.88^wxyza1b1c1d1e1^ |
| JMU-Bael (Sel-76) | 5.34±0.2^k1l1^ | 4.91±0.02^p1^ | 2.95±0.04^q1^ | 10.67±0.58^lm^ | 8.33±0.58^mno^ | 87.33±2.08^za1b1^ | 13.59±0.07^d1e1^ | 15.57±0.29^h1i1j1^ |
| JMU-Bael (Sel-77) | 5.15±0.26^l1m1^ | 6.79±0.03^uvwx^ | 5.64±0.04^qrst^ | 12.33±0.58^ij^ | 5±1^t^ | 54.67±4.51^k1l1m1^ | 10.2±0.13^j1k1l1^ | 18.72±1.32^qrstuvwxyza1b1c1^ |
| JMU-Bael (Sel-78) | 5.59±0.15^h1i1j1^ | 3.70±0.04^r1^ | 2.48±0.03^r1^ | 10.67±0.58^lm^ | 3.67±0.58^uv^ | 38.33±4.73^q1^ | 7.56±0.1^o1^ | 19.91±2.34^mnopqrstuvw^ |
| JMU-Bael (Sel-79) | 6.7±0.3^a1b1c1^ | 8.45±0.04^efg^ | 4.91±0.03^yza1^ | 14.67±0.58^de^ | 9.33±0.58^klm^ | 134.67±3.06^lmno^ | 25.04±1.56^kl^ | 18.59±1.08^stuvwxyza1b1c1^ |
| JMU-Bael (Sel-80) | 5.38±0.15^i1j1k1l1^ | 6.13±0.04^e1f1g1^ | 4.63±0.04^c1d1^ | 8.67±0.58^pq^ | 10.33±0.58^hijk^ | 90.33±2.52^yz^ | 20.56±0.08^pq^ | 22.77±0.54^defgh^ |
| NB-5 | 8.72±0.06^mno^ | 8.27±0.03^ghi^ | 5.35±0.03^w^ | 15.33±0.58^cd^ | 11.33±0.58^fgh^ | 167±2.65^bc^ | 39.79±0.31^b^ | 23.83±0.19^bcd^ |
| NB-9 | 9.03±0.03^ijklm^ | 8.6±0.02^e^ | 6.56±0.03^de^ | 15.33±0.58^cd^ | 9.33±0.58^klm^ | 138±3^klmn^ | 29.73±0.47^h^ | 21.54±0.17^fghijklm^ |
| **C.D. at 5%** | 0.23 | 0.24 | 0.18 | 0.89 | 0.84 | 3.90 | 1.29 | 1.72 |

Value represent the mean±SD (Standard deviation)
Mean with different letters in each column is significantly different from each other at *P*< 0.05 (Fisher’s least significant difference)
