# Supplementary material for: Morphology and pomological characterization of bael [Aegle marmelos (L.) Correa] genotypes for climate change mitigation under north-western Himalayas
Source: Front Plant Sci. 2025 Mar 18;16:1496769. doi: 10.3389/fpls.2025.1496769 (PMC11962431; doi:10.3389/fpls.2025.1496769)
Supplement: Supplementary file 3 [file DataSheet3.docx]

| **Genotypes** | **Fruit maturity group** | **Average date of fruit maturity** | **Immature fruit colour** | **Mature fruit colour**  **Table S4 Morphological characteristics of bael genotypes.** | **Styler end cavity** | **Stem end cavity** |
| --- | --- | --- | --- | --- | --- | --- |
| JMU-Bael (Sel-1) | Mid | 2nd week of April | Light green | Greenish pale yellow | Shallow | Shallow |
| JMU-Bael (Sel-2) | Mid | 2nd week of April | Light green | Greenish pale yellow | Shallow | Shallow |
| JMU-Bael (Sel-3) | Mid | 1st week of April | Light green | Greenish pale yellow | Shallow | Shallow |
| JMU-Bael (Sel-4) | Early | 4th week of March | Light green | Greenish pale yellow | Shallow | Shallow |
| JMU-Bael (Sel-5) | Mid | 2nd week of April | Light green | Greenish pale yellow | Depressed | Depressed |
| JMU-Bael (Sel-6) | Mid | 1st week of April | Light green | Greenish pale yellow | Shallow | Shallow |
| JMU-Bael (Sel-7) | Mid | 2nd week of April | Light green | Greenish pale yellow | Shallow | Shallow |
| JMU-Bael (Sel-8) | Mid | 2nd week of April | Light green | Greenish pale yellow | Shallow | Shallow |
| JMU-Bael (Sel-9) | Early | 4th week of March | Light green | Greenish pale yellow | Shallow | Shallow |
| JMU-Bael (Sel-10) | Late | 3rd week of April | Green | Greenish pale yellow | Shallow | Shallow |
| JMU-Bael (Sel-11) | Late | 3rd week of April | Light green | Greenish pale yellow | Shallow | Shallow |
| JMU-Bael (Sel-12) | Late | 3rd week of April | Green | Greenish pale yellow | Shallow | Shallow |
| JMU-Bael (Sel-13) | Mid | 2nd week of April | Light green | Greenish pale yellow | Shallow | Shallow |
| JMU-Bael (Sel-14) | Early | 4th week of March | Light green | Greenish pale yellow | Shallow | Shallow |
| JMU-Bael (Sel-15) | Mid | 2nd week of April | Light green | Greenish pale yellow | Shallow | Shallow |
| JMU-Bael (Sel-16) | Early | 4th week of March | Green | Greenish pale yellow | Shallow | Shallow |
| JMU-Bael (Sel-17) | Mid | 2nd week of April | Light green | Yellowish green | Shallow | Shallow |
| JMU-Bael (Sel-18) | Mid | 1st week of April | Light green | Greenish pale yellow | Shallow | Shallow |
| JMU-Bael (Sel-19) | Late | 3rd week of April | Light green | Greenish pale yellow | Shallow | Shallow |
| JMU-Bael (Sel-20) | Mid | 1st week of April | Green | Greenish pale yellow | Shallow | Shallow |
| JMU-Bael (Sel-21) | Mid | 1st week of April | Light green | Yellowish green | Shallow | Shallow |
| JMU-Bael (Sel-22) | Mid | 2nd week of April | Light green | Yellowish green | Shallow | Shallow |
| JMU-Bael (Sel-23) | Mid | 2nd week of April | Light green | Greenish pale yellow | Shallow | Shallow |
| JMU-Bael (Sel-24) | Early | 4th week of March | Light green | Greenish pale yellow | Shallow | Shallow |
| JMU-Bael (Sel-25) | Early | 4th week of March | Light green | Greenish pale yellow | Shallow | Shallow |
| JMU-Bael (Sel-26) | Mid | 2nd week of April | Light green | Yellowish green | Shallow | Shallow |
| JMU-Bael (Sel-27) | Mid | 1st week of April | Light green | Greenish pale yellow | Shallow | Shallow |
| JMU-Bael (Sel-28) | Late | 3rd week of April | Light green | Greenish pale yellow | Shallow | Shallow |
| JMU-Bael (Sel-29) | Mid | 2nd week of April | Light green | Greenish pale yellow | Shallow | Shallow |
| JMU-Bael (Sel-30) | Mid | 1st week of April | Light green | Greenish pale yellow | Shallow | Shallow |
| JMU-Bael (Sel-31) | Mid | 1st week of April | Light green | Greenish pale yellow | Depressed | Shallow |
| JMU-Bael (Sel-32) | Mid | 1st week of April | Light green | Greenish pale yellow | Shallow | Shallow |
| JMU-Bael (Sel-33) | Early | 4th week of March | Light green | Greenish pale yellow | Shallow | Shallow |
| JMU-Bael (Sel-34) | Mid | 2nd week of April | Light green | Greenish pale yellow | Shallow | Shallow |
| JMU-Bael (Sel-35) | Mid | 2nd week of April | Light green | Greenish pale yellow | Shallow | Shallow |
| JMU-Bael (Sel-36) | Early | 4th week of March | Light green | Greenish pale yellow | Shallow | Shallow |
| JMU-Bael (Sel-37) | Mid | 1st week of April | Light green | Greenish pale yellow | Shallow | Shallow |
| JMU-Bael (Sel-38) | Mid | 2nd week of April | Green | Greenish pale yellow | Shallow | Shallow |
| JMU-Bael (Sel-39) | Mid | 1st week of April | Green | Greenish pale yellow | Shallow | Shallow |
| JMU-Bael (Sel-40) | Early | 4th week of March | Light green | Greenish pale yellow | Shallow | Shallow |
| JMU-Bael (Sel-41) | Early | 4th week of March | Green | Green | Shallow | Shallow |
| JMU-Bael (Sel-42) | Mid | 1st week of April | Light green | Yellowish green | Shallow | Shallow |
| JMU-Bael (Sel-43) | Late | 3rd week of April | Light green | Yellowish green | Shallow | Shallow |
| JMU-Bael (Sel-44) | Mid | 2nd week of April | Green | Yellowish green | Shallow | Shallow |
| JMU-Bael (Sel-45) | Mid | 2nd week of April | Light green | Green | Shallow | Shallow |
| JMU-Bael (Sel-46) | Mid | 1st week of April | Light green | Greenish pale yellow | Shallow | Shallow |
| JMU-Bael (Sel-47) | Mid | 1st week of April | Light green | Greenish pale yellow | Shallow | Shallow |
| JMU-Bael (Sel-48) | Late | 3rd week of April | Green | Greenish pale yellow | Shallow | Shallow |
| JMU-Bael (Sel-49) | Late | 3rd week of April | Light green | Yellowish green | Shallow | Shallow |
| JMU-Bael (Sel-50) | Late | 3rd week of April | Green | Greenish pale yellow | Shallow | Shallow |
| JMU-Bael (Sel-51) | Late | 3rd week of April | Green | Yellowish green | Shallow | Shallow |
| JMU-Bael (Sel-52) | Late | 3rd week of April | Dark green | Green | Shallow | Shallow |
| JMU-Bael (Sel-53) | Late | 3rd week of April | Light green | Greenish pale yellow | Shallow | Shallow |
| JMU-Bael (Sel-54) | Mid | 2nd week of April | Dark green | Greenish pale yellow | Shallow | Shallow |
| JMU-Bael (Sel-55) | Mid | 2nd week of April | Light green | Greenish pale yellow | Shallow | Shallow |
| JMU-Bael (Sel-56) | Early | 4th week of March | Light green | Greenish pale yellow | Shallow | Shallow |
| JMU-Bael (Sel-57) | Mid | 2nd week of April | Green | Greenish pale yellow | Shallow | Shallow |
| JMU-Bael (Sel-58) | Mid | 1st week of April | Light green | Greenish pale yellow | Shallow | Shallow |
| JMU-Bael (Sel-59) | Mid | 2nd week of April | Green | Yellowish green | Shallow | Shallow |
| JMU-Bael (Sel-60) | Late | 3rd week of April | Green | Greenish pale yellow | Shallow | Shallow |
| JMU-Bael (Sel-61) | Late | 3rd week of April | Green | Green | Shallow | Shallow |
| JMU-Bael (Sel-62) | Late | 3rd week of April | Light green | Green | Shallow | Shallow |
| JMU-Bael (Sel-63) | Late | 3rd week of April | Dark green | Green | Shallow | Shallow |
| JMU-Bael (Sel-64) | Late | 3rd week of April | Dark green | Green | Shallow | Shallow |
| JMU-Bael (Sel-65) | Late | 3rd week of April | Dark green | Green | Shallow | Shallow |
| JMU-Bael (Sel-66) | Late | 3rd week of April | Dark green | Green | Shallow | Shallow |
| JMU-Bael (Sel-67) | Mid | 1st week of April | Light green | Yellowish green | Shallow | Shallow |
| JMU-Bael (Sel-68) | Mid | 1st week of April | Green | Green | Shallow | Shallow |
| JMU-Bael (Sel-69) | Mid | 2nd week of April | Light green | Greenish pale yellow | Shallow | Shallow |
| JMU-Bael (Sel-70) | Mid | 1st week of April | Green | Green | Shallow | Shallow |
| JMU-Bael (Sel-71) | Mid | 2nd week of April | Light green | Green | Shallow | Depressed |
| JMU-Bael (Sel-72) | Late | 3rd week of April | Light green | Greenish pale yellow | Shallow | Shallow |
| JMU-Bael (Sel-73) | Mid | 1st week of April | Light green | Greenish pale yellow | Shallow | Depressed |
| JMU-Bael (Sel-74) | Early | 4th week of March | Light green | Greenish pale yellow | Depressed | Shallow |
| JMU-Bael (Sel-75) | Late | 3rd week of April | Green | Greenish pale yellow | Shallow | Depressed |
| JMU-Bael (Sel-76) | Late | 3rd week of April | Light green | Greenish pale yellow | Shallow | Shallow |
| JMU-Bael (Sel-77) | Late | 3rd week of April | Light green | Greenish pale yellow | Shallow | Depressed |
| JMU-Bael (Sel-78) | Late | 3rd week of April | Light green | Greenish pale yellow | Shallow | Shallow |
| JMU-Bael (Sel-79) | Late | 3rd week of April | Light green | Greenish pale yellow | Shallow | Depressed |
| JMU-Bael (Sel-80) | Mid | 1st week of April | Light green | Greenish pale yellow | Shallow | Shallow |
| NB-5 | Late | 3rd week of April | Green | Greenish pale yellow | Depressed | Shallow |
| NB-9 | Mid | 1st week of April | Dark green | Green | Shallow | Shallow |

Continue Table 2

Continue Table 2

| **Genotypes** | **Fruit surface** | **Fruit Shape** | **Fruit skull colour** | **Locule arrangement** | **Arrangement of seed in pulp** | **Seed shape** |
| --- | --- | --- | --- | --- | --- | --- |
| JMU-Bael (Sel-1) | Smooth | Elliptical | Creamish Yellow | Centric | Arranged in straight line | Oblong |
| JMU-Bael (Sel-2) | Smooth | Elliptical | Greenish Yellow | Highly centric | Arranged in straight line | Oblong |
| JMU-Bael (Sel-3) | Smooth | Elliptical | Greenish Yellow | Highly centric | Arranged in straight line | Round |
| JMU-Bael (Sel-4) | Smooth | Globose | Greenish Yellow | Highly centric | Arranged in straight line | Round |
| JMU-Bael (Sel-5) | Smooth | Round | Greenish Yellow | Centric | Arranged in straight line | Oblong |
| JMU-Bael (Sel-6) | Smooth | Globose | Greenish Yellow | Centric | Arranged in straight line | Oblong |
| JMU-Bael (Sel-7) | Smooth | Round | Greenish Yellow | Centric | Arranged in straight line | Oblong |
| JMU-Bael (Sel-8) | Smooth | Round | Greenish Yellow | Centric | Arranged in straight line | Oblong |
| JMU-Bael (Sel-9) | Smooth | Globose | Greenish Yellow | Centric | Arranged in straight line | Round |
| JMU-Bael (Sel-10) | Smooth | Round | Greenish Yellow | Centric | Arranged in straight line | Oblong |
| JMU-Bael (Sel-11) | Rough | Round | Greenish Yellow | Centric | Arranged in straight line | Round |
| JMU-Bael (Sel-12) | Smooth | Ovate | Greenish Yellow | Centric | Arranged in straight line | Round |
| JMU-Bael (Sel-13) | Smooth | Round | Greenish Yellow | Centric | Arranged in straight line | Oblong |
| JMU-Bael (Sel-14) | Smooth | Ovate | Greenish Yellow | Centric | Arranged in straight line | Oblong |
| JMU-Bael (Sel-15) | Smooth | Round | Creamish Yellow | Centric | Arranged in straight line | Oblong |
| JMU-Bael (Sel-16) | Smooth | Round | Greenish Yellow | Centric | Arranged in straight line | Oblong |
| JMU-Bael (Sel-17) | Smooth | Round | Creamish Yellow | Centric | Arranged in straight line | Round |
| JMU-Bael (Sel-18) | Smooth | Elliptical | Russet Yellow | Highly centric | Arranged in straight line | Round |
| JMU-Bael (Sel-19) | Smooth | Round | Greenish Yellow | Centric | Arranged in straight line | Round |
| JMU-Bael (Sel-20) | Rough | Round | Greenish Yellow | Centric | Arranged in straight line | Oblong |
| JMU-Bael (Sel-21) | Smooth | Round | Russet Yellow | Centric | Arranged in straight line | Oblong |
| JMU-Bael (Sel-22) | Smooth | Round | Russet Yellow | Centric | Arranged in straight line | Round |
| JMU-Bael (Sel-23) | Smooth | Globose | Greenish Yellow | Centric | Arranged in straight line | Oblong |
| JMU-Bael (Sel-24) | Smooth | Round | Greenish Yellow | Centric | Arranged in straight line | Oblong |
| JMU-Bael (Sel-25) | Smooth | Round | Russet Yellow | Centric | Arranged in straight line | Oblong |
| JMU-Bael (Sel-26) | Smooth | Round | Russet Yellow | Centric | Arranged in straight line | Oblong |
| JMU-Bael (Sel-27) | Smooth | Round | Greenish Yellow | Centric | Arranged in straight line | Oblong |
| JMU-Bael (Sel-28) | Smooth | Round | Greenish Yellow | Centric | Arranged in straight line | Round |
| JMU-Bael (Sel-29) | Smooth | Round | Creamish Yellow | Centric | Arranged in straight line | Round |
| JMU-Bael (Sel-30) | Smooth | Globose | Greenish Yellow | Scattered | Arranged in straight line | Oblong |
| JMU-Bael (Sel-31) | Smooth | Globose | Greenish Yellow | Centric | Arranged in straight line | Oblong |
| JMU-Bael (Sel-32) | Smooth | Ovate | Greenish | Centric | Arranged in straight line | Oblong |
| JMU-Bael (Sel-33) | Rough | Globose | Greenish Yellow | Centric | Arranged in straight line | Oblong |
| JMU-Bael (Sel-34) | Rough | Globose | Greenish Yellow | Centric | Arranged in straight line | Oblong |
| JMU-Bael (Sel-35) | Rough | Round | Greenish Yellow | Centric | Arranged in straight line | Round |
| JMU-Bael (Sel-36) | Smooth | Globose | Greenish Yellow | Centric | Arranged in straight line | Oblong |
| JMU-Bael (Sel-37) | Smooth | Round | Creamish Yellow | Centric | Arranged in straight line | Oblong |
| JMU-Bael (Sel-38) | Smooth | Round | Greenish Yellow | Centric | Arranged in straight line | Oblong |
| JMU-Bael (Sel-39) | Smooth | Round | Dull white | Highly centric | Arranged in straight line | Oblong |
| JMU-Bael (Sel-40) | Smooth | Round | Creamish Yellow | Centric | Arranged in straight line | Round |
| JMU-Bael (Sel-41) | Smooth | Round | Greenish | Centric | Arranged in straight line | Oblong |
| JMU-Bael (Sel-42) | Smooth | Elliptical | Greenish Yellow | Centric | Arranged in straight line | Oblong |
| JMU-Bael (Sel-43) | Smooth | Elliptical | Greenish Yellow | Centric | Arranged in straight line | Oblong |
| JMU-Bael (Sel-44) | Smooth | Globose | Creamish Yellow | Scattered | Distributed in whole pulp | Oblong |
| JMU-Bael (Sel-45) | Smooth | Round | Greenish Yellow | Centric | Arranged in straight line | Oblong |
| JMU-Bael (Sel-46) | Smooth | Elliptical | Creamish Yellow | Scattered | Arranged in straight line | Oblong |
| JMU-Bael (Sel-47) | Smooth | Round | Greenish | Highly centric | Arranged in straight line | Oblong |
| JMU-Bael (Sel-48) | Rough | Round | Greenish Yellow | Scattered | Arranged in straight line | Round |
| JMU-Bael (Sel-49) | Smooth | Elliptical | Greenish Yellow | Centric | Arranged in straight line | Oblong |
| JMU-Bael (Sel-50) | Rough | Elliptical | Greenish Yellow | Centric | Arranged in straight line | Round |
| JMU-Bael (Sel-51) | Rough | Round | Greenish Yellow | Centric | Arranged in straight line | Oblong |
| JMU-Bael (Sel-52) | Rough | Round | Greenish | Centric | Arranged in straight line | Round |
| JMU-Bael (Sel-53) | Smooth | Elliptical | Greenish | Centric | Arranged in straight line | Oblong |
| JMU-Bael (Sel-54) | Rough | Round | Greenish | Centric | Arranged in straight line | Round |
| JMU-Bael (Sel-55) | Smooth | Globose | Greenish Yellow | Scattered | Distributed in whole pulp | Oblong |
| JMU-Bael (Sel-56) | Smooth | Round | Dull white | Centric | Arranged in straight line | Oblong |
| JMU-Bael (Sel-57) | Smooth | Globose | Greenish | Centric | Arranged in straight line | Oblong |
| JMU-Bael (Sel-58) | Smooth | Globose | Greenish Yellow | Centric | Arranged in straight line | Oblong |
| JMU-Bael (Sel-59) | Smooth | Globose | Creamish Yellow | Centric | Arranged in straight line | Oblong |
| JMU-Bael (Sel-60) | Smooth | Ovate | Greenish Yellow | Centric | Arranged in straight line | Oblong |
| JMU-Bael (Sel-61) | Smooth | Globose | Greenish Yellow | Centric | Arranged in straight line | Oblong |
| JMU-Bael (Sel-62) | Rough | Globose | Greenish Yellow | Centric | Arranged in straight line | Oblong |
| JMU-Bael (Sel-63) | Rough | Round | Greenish | Centric | Arranged in straight line | Oblong |
| JMU-Bael (Sel-64) | Rough | Globose | Greenish | Centric | Arranged in straight line | Oblong |
| JMU-Bael (Sel-65) | Rough | Round | Greenish | Centric | Arranged in straight line | Oblong |
| JMU-Bael (Sel-66) | Rough | Round | Greenish | Centric | Arranged in straight line | Round |
| JMU-Bael (Sel-67) | Smooth | Globose | Creamish Yellow | Centric | Arranged in straight line | Oblong |
| JMU-Bael (Sel-68) | Smooth | Round | Greenish | Centric | Arranged in straight line | Round |
| JMU-Bael (Sel-69) | Smooth | Round | Greenish Yellow | Centric | Arranged in straight line | Round |
| JMU-Bael (Sel-70) | Smooth | Globose | Greenish | Centric | Arranged in straight line | Round |
| JMU-Bael (Sel-71) | Smooth | Round | Greenish | Centric | Arranged in straight line | Round |
| JMU-Bael (Sel-72) | Rough | Round | Creamish Yellow | Centric | Arranged in straight line | Oblong |
| JMU-Bael (Sel-73) | Rough | Globose | Creamish Yellow | Centric | Arranged in straight line | Round |
| JMU-Bael (Sel-74) | Smooth | Round | Greenish Yellow | Centric | Arranged in straight line | Oblong |
| JMU-Bael (Sel-75) | Rough | Round | Greenish Yellow | Centric | Arranged in straight line | Oblong |
| JMU-Bael (Sel-76) | Rough | Round | Greenish Yellow | Centric | Arranged in straight line | Oblong |
| JMU-Bael (Sel-77) | Smooth | Globose | Greenish Yellow | Centric | Arranged in straight line | Oblong |
| JMU-Bael (Sel-78) | Rough | Round | Greenish Yellow | Centric | Arranged in straight line | Oblong |
| JMU-Bael (Sel-79) | Rough | Globose | Greenish Yellow | Centric | Arranged in straight line | Oblong |
| JMU-Bael (Sel-80) | Smooth | Round | Greenish Yellow | Centric | Arranged in straight line | Round |
| NB-5 | Smooth | Round | Creamish Yellow | Centric | Arranged in straight line | Round |
| NB-9 | Rough | Ovate | Greenish | Highly centric | Arranged in straight line | Oblong |
